# Synthesis of a Fluorous-Tagged Hexasaccharide and Interaction with Growth Factors Using Sugar-Coated Microplates

**DOI:** 10.3390/molecules24081591

**Published:** 2019-04-22

**Authors:** Susana Maza, José L. de Paz, Pedro M. Nieto

**Affiliations:** Glycosystems Laboratory, Instituto de Investigaciones Químicas (IIQ), cicCartuja, CSIC and Universidad de Sevilla, Americo Vespucio, 49, 41092 Sevilla, Spain; susmaza@gmail.com

**Keywords:** oligosaccharide synthesis, fluorous tag, glycosaminoglycan mimetics, carbohydrate-protein interactions

## Abstract

Here, we report the synthesis of a sulfated, fully protected hexasaccharide as a glycosaminoglycan mimetic and the study of its interactions with different growth factors: midkine, basic fibroblast growth factor (FGF-2) and nerve growth factor (NGF). Following a fluorous-assisted approach, monosaccharide building blocks were successfully assembled and the target oligosaccharide was prepared in excellent yield. The use of more acid stable 4,6-*O*-silylidene protected glucosamine units was crucial for the efficiency of this strategy because harsh reaction conditions were needed in the glycosylations to avoid the formation of orthoester side products. Fluorescence polarization experiments demonstrated the strong interactions between the synthesized hexamer, and midkine and FGF-2. In addition, we have developed an alternative assay to analyse these molecular recognition events. The prepared oligosaccharide was non-covalently attached to a fluorous-functionalized microplate and the direct binding of the protein to the sugar-immobilized surface was measured, affording the corresponding K_D,surf_ value.

## 1. Introduction

Growth factors are a family of secreted, low molecular weight proteins that regulate cell proliferation and differentiation. Many of these growth factors bind to glycosaminoglycans (GAGs), including heparin and chondroitin sulfate (CS), which are heterogeneous polysaccharides found ubiquitously in mammalian tissues. This binding is essential for the biological activities of growth factors, and usually involves particular GAG sequences with well-defined structures. For instance, midkine is a heparin-binding growth factor that promotes growth, survival and differentiation of target cells [1,2]. This cytokine is implicated in many diseases, such as cancer and multiple sclerosis. It has been demonstrated that midkine interacts with chondroitin sulfate E (CS-E), a subtype of this polysaccharide characterized by the disulfated repeating unit d-glucuronic acid (GlcA)-β(1→3)-*N*-acetyl-d-galactosamine (GalNAc)(4,6-di-OSO_3_)-β(1→4) [3,4]. The interaction of midkine with CS is sensitive to the location of the sulfate groups along the sugar backbone [5]. Basic fibroblast growth factor (FGF-2) is another protein that plays a key role in cell growth, mediating the formation of new blood vessels [6]. FGF-2 also binds with nanomolar affinity to heparin and CS-E [3]. On the other hand, nerve growth factor (NGF) is a neurotrophin involved in the development, maintenance and survival of the vertebrate nervous system [7]. Hsieh-Wilson and coworkers showed for the first time that CS-E is able to modulate NGF signaling pathways [8], although in this case the interaction GAG-protein is weaker, in the micromolar range [9].

The preparation of molecules that mimic the properties of GAGs and bind to growth factors with high affinity is of great interest [10,11,12]. Such compounds could potentially control the biological functions of these proteins and have applications in tissue repair and anticancer therapies. In fact, the synthesis of different types of GAG mimetics has been reported, including GAG multivalent systems [13,14,15,16,17,18,19,20,21,22,23]. In this context, we previously found that sulfated oligosaccharides displaying hydrophobic protecting groups can be considered as GAG mimetics, strongly interacting with growth factors such as midkine and FGF-2 [24]. Interestingly, the binding affinities of these protected oligomers were much higher than those corresponding to the fully deprotected compounds [24]. A tetrasaccharide derivative following the sequence d-glucosamine(GlcN)(4,6-di-OSO_3_)-β(1→4)-d-glucose(Glc)-β(1→3) was synthesized using a fluorous-tag assisted approach and its interaction with midkine and FGF-2 was demonstrated by fluorescence polarization measurements [25]. In this work, we present the synthesis of the analogous hexasaccharide chain in order to study the influence of sugar length on the growth factor binding affinity. The synthetic strategy was optimized for the efficient preparation of this complex oligosaccharide. Moreover, we developed an experimental protocol for the interaction study between this sugar derivative and several growth factors based on the immobilization of the fluorinated carbohydrate on standard microtiter plates. These binding data were compared with the results obtained from fluorescence polarization assays.

## 2. Results and Discussion

**Scheme 1:**
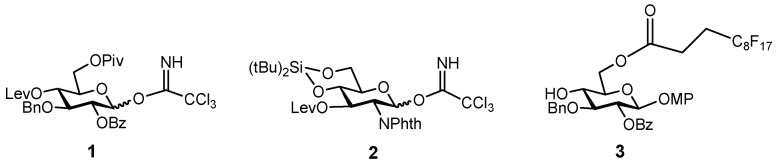
Monosaccharide units employed for the preparation of the target hexasaccharide. Lev = levulinoyl; Piv = pivaloyl; MP = 4-methoxyphenyl; NPhth = *N*-phthaloyl.

**Scheme 2:**
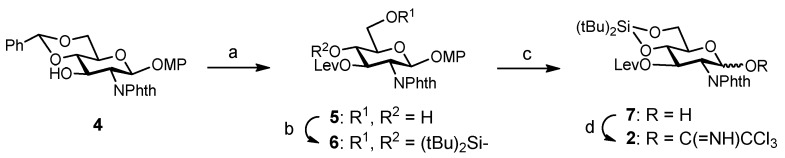
Reagents and conditions: (**a**) Lev_2_O, DMAP, CH_2_Cl_2_; TFA, CH_2_Cl_2_-H_2_O, 0 °C → room temperature, 87%; (**b**) *t*Bu_2_Si(OTf)_2_, Py, 0 °C → room temperature, 93%; (**c**) CAN, CH_2_Cl_2_-CH_3_CN-H_2_O, 0 °C, 91%; (**d**) Cl_3_CCN, DBU, CH_2_Cl_2_, 75%.

The synthesis of the target hexasaccharide was accomplished by assembling the monosaccharide building blocks **1**, **2** and **3** shown in Scheme 1. The trichloroacetimidate method was chosen to construct the glycosidic bonds [26,27]. Participating protecting groups at position 2 in glycosyl donors **1** and **2** (benzoyl and *N*-phthalimido) favoured the selective formation of the desired β linkages. Glucose derivative **3** constituted the reducing end of the oligosaccharide chain. The C_8_F_17_ tail at the 6-OH position allowed the separation of fluorinated intermediates by fluorous solid-phase extraction (F-SPE) [28,29,30,31], avoiding time-consuming chromatographic purifications. Both glucose units **1** and **3** were prepared as previously reported, starting from diacetone d-glucose [25,32]. On the other hand, glucosamine trichloroacetimidate **2** was synthesized from commercially available **4** (Scheme 2). Treatment with levulinic anhydride and catalytic DMAP (4-dimethylamino pyridine) followed by trifluoroacetic acid (TFA)-mediated hydrolysis of the benzylidene acetal afforded diol **5** in excellent yield. Hydroxyl functions at positions 4 and 6 were then masked with a cyclic silylidene group by using di-*tert*-butylsilyl bis(trifluoromethanesulfonate) in dry pyridine. Thus, compound **6** was obtained in 93% yield. This silyl group can be selectively removed at the end of the synthesis in order to install the sulfates at the desired positions. Notably, in our previous synthesis of a tetrasaccharide GAG mimetic, we used 4,6-*O*-benzylidene glucosamine derivatives [25]. However, unwanted cleavage of the benzylidene moiety can occur under the acidic conditions of glycosylation reactions. For this reason, we decided to employ the more acid stable di-*tert*-butylsilylidene group. The advantages of this function over the alternative benzylidene acetals were shown by Codee and coworkers during the synthesis of hyaluronic acid oligomers [33]. Oxidative removal of the 4-methoxyphenyl moiety at the anomeric position using cerium (IV) ammonium nitrate (CAN) and trichloroacetimidate activation with trichloroacetonitrile and catalytic DBU (1,8-diazabicycloundec-7-ene) finally gave glycosyl donor **2**.

**Scheme 3:**
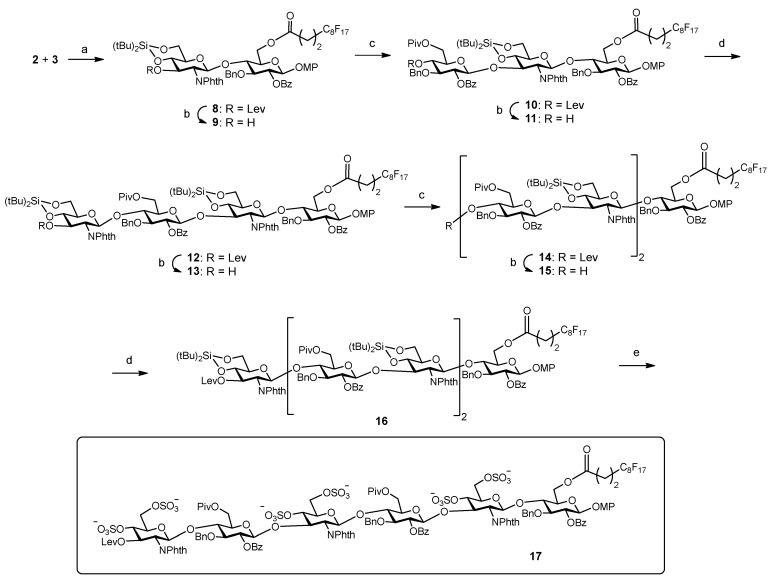
Reagents and conditions: (**a**) TBSOTf, CH_2_Cl_2_, 0 °C; (**b**) NH_2_NH_2_·H_2_O, Py-AcOH, CH_2_Cl_2_; (**c**) **1**, TBSOTf, CH_2_Cl_2_, room temperature; (**d**) **2**, TBSOTf, CH_2_Cl_2_, room temperature; **16**: 39% from **3**, 9 steps; (**e**) (HF)*_n_*·Py, THF, 0 °C; SO_3_·Me_3_N, DMF, 100 °C, MW heating, 56%.

Next, we proceeded with the fluorous-tag assisted coupling of the building blocks to generate the target hexasaccharide sequence (Scheme 3). Glycosylation between fluorinated glucose **3** and glucosamine **2** (2 equiv.) was performed at 0 °C using TBSOTf (*tert*-butyldimethylsilyl trifluoromethanesulfonate) instead of TMSOTf (trimethylsilyl trifluoromethanesulfonate) in order to avoid the formation of the trimethylsilylated acceptor byproduct. After F-SPE purification, NMR, TLC and mass spectrometry analysis indicated the clean formation of disaccharide **8**. Treatment with hydrazine monohydrate in pyridine/acetic acid buffer selectively removed the levulinoyl group providing disaccharide acceptor **9** ready for subsequent chain elongation. Synthesis of trisaccharide **10** was then accomplished. In previous work [25], we observed the formation of an orthoester side product [34] in the condensation between donor **1** and a 3-OH GlcN-Glc derivative, closely related to disaccharide acceptor **9**. Increased amounts of Lewis acid catalyst were required to prevent the formation of the orthoester. Similarly, the first glycosylation trial of building blocks **1** and **9** using 10 mol% of TBSOTf with respect to the donor at 0 °C gave the orthoester trisaccharide as the major product. Taking advantage of the high acid stability of the silylidene, harsher reaction conditions (20 mol% of TBSOTf with respect to the donor at room temperature) were tested. Thus, compound **9** was coupled with 2.5 equiv. of trichloroacetimidate **1** to afford the desired trimer **10**. The crude mixture was quickly purified by F-SPE and the study of the fluorinated fraction by NMR, TLC and mass spectrometry revealed the generation of **10** as the main reaction product (see NMR spectra, Supplementary Materials). A small amount of unreacted **9** (<10%) was also identified. Therefore, conventional acetylation (Ac_2_O, Py, DMAP, overnight) was carried out in order to block the remaining acceptor **9** and prevent deletion sequence problems [35]. Iterative delevulination and glycosylation with glucosamine **2** (2.8 equiv.) smoothly led to tetramer **12**. Subsequent deprotection of the temporary levulinoyl group gave acceptor **13** that was treated with donor **1** (3 equiv.) to obtain pentasaccharide **14**. After this first glycosylation cycle, F-SPE was performed and a significant amount (around 25%) of unreacted tetrasaccharide **13** was detected in the fluorous elution. In order to complete the reaction, a second glycosylation cycle with additional **1** (2 equiv.) was run. Interestingly, our results suggest that the construction of the Glc-GlcN bonds is less efficient in terms of consumption of donor building blocks than the formation of the GlcN-Glc linkages. Then, levulinoyl deprotection and condensation with glucosamine **2** (3 equiv.) afforded the desired compound **16**. After F-SPE and standard silica gel column chromatography, pure hexasaccharide **16** was isolated in an excellent 39% yield, which means a 90% average yield per step. Finally, the three silylidene groups in **16** were selectively removed with (HF)_n_·Py complex in THF at 0 °C and the resulting hexaol was extensively sulfated with SO_3_·NMe_3_ at 100 ºC using microwave heating [36] to give sulfated derivative **17**. Three sulfation cycles were required to complete the reaction and minimize partially sulfated intermediates.

As previously reported [25], treatment of compounds, such as **17**, with ethylene diamine followed by *N*-acetylation and hydrogenolysis could afford a GAG-like deprotected sequence. However, in previous papers [24,25], we found that the binding affinities of these fully deprotected oligosaccharides are much lower than those corresponding to the sulfated, protected intermediates. For this reason, in this work we focus on the binding properties of hexasaccharide **17**.

Thus, we initiated the study of the interactions between **17** and midkine, FGF-2 and NGF growth factors. First, we estimated the solution-phase binding affinities using our previously reported fluorescence polarization (FP) competition experiment (see Supplementary Materials) [37]. Briefly, we measured the ability of hexasaccharide **17** to compete with a fluorescent probe for protein binding. Thus, we calculated IC_50_ values as **17** concentration required for 50% inhibition. As shown in Table 1, our results indicated that derivative **17** interacted with midkine and FGF-2 in the nano- and micromolar range, respectively. These relative binding affinities were similar to those previously found for the analogous tetrasaccharide, although slightly lower (IC_50_ = 270 nM and 2.4 µM for the tetramer/midkine and tetramer/FGF-2 interactions, respectively) [25]. Our results indicated that the increase in oligosaccharide length, from tetra- to hexasaccharide, does not enhance growth factor binding. We also attempted to measure the binding of **17** to NGF. However, we could not apply our FP competition assay to the study of this interaction because NGF did not significantly bind to the probe, a fluorescently labelled heparin sequence, even at 3.7 µM concentration. This fact suggested that NGF displays less affinity for GAGs compared to midkine and FGF-2, in agreement with literature data (see also below) [3,9].

**Figure 1:**
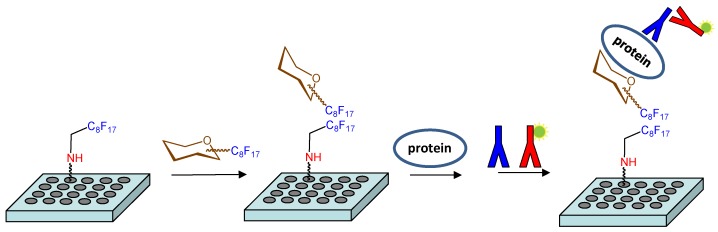
Schematic representation of the strategy used to study the interactions between fluorinated sugars and proteins on microtiter plates.

An alternative strategy was also considered to study these molecular recognition events. This approach involved the non-covalent immobilization of the fluorinated hexamer **17** on microtiter plates (Figure 1). Fluorous-based microarrays for the investigation of carbohydrate-protein interactions have been previously developed. A C_8_F_17_ tail was employed for the noncovalent immobilization of sugar derivatives on fluorous coated glass slides [38,39,40,41]. However, to the best of our knowledge, an experimental protocol to study the interactions between fluorous sugar-coated conventional microplates and proteins has not been published. For this purpose, we first treated an amino reactive 384-well plate (Nunc Immobilizer Amino^TM^) with a 1 mM solution of heptadecafluorononylamine (C_8_F_17_CH_2_NH_2_) in DMSO. Thus, we created a fluorinated surface ready for the immobilization of compound **17** through noncovalent fluorous-fluorous interactions. After quenching with ethanolamine, microplate wells were filled with a 250 µM solution of **17** in water/DMSO 9:1, incubated at room temperature for 6 h, and extensively washed with water. Some wells were not treated with **17** and were used as blank samples. Then, the binding of the attached sugar **17** to growth factors was analyzed. Incubation with different concentrations of midkine, FGF-2 and NGF was carried out and the bound protein was detected by further incubations with the corresponding primary and fluorescently labelled secondary antibodies (see Materials and Methods). Finally, the fluorescence was recorded using a standard microplate reader. The observation of fluorescence signals confirmed the interaction between immobilized **17** and the protein at a particular concentration. Fluorescence was observed neither from wells incubated only with the antibodies, in the absence of the protein, nor from blank wells coated with the fluorous C_8_F_17_CH_2_NH_2_ linker alone, in the absence of sugar **17**, ruling out the non-specific binding of the proteins on the fluorous linker-coated wells.

Importantly, we performed a control experiment in which sugar **3**, instead of hexamer **17**, was immobilized on the fluorous-functionalized plate (see Supplementary Materials). No fluorescence signals above background were detected from these wells, discarding the non-specific interaction between any fluorous-tagged sugar and the growth factors. In addition, wells coated with hexamer **17** were incubated with two fluorescently labelled non-GAG binding proteins, Concanavalin A and bovine serum albumin, at 11 µM concentration (see Supplementary Materials). Again, we observed no fluorescence signals from these wells. These results indicated that compound **17** did not interact with Concanavalin A and bovine serum albumin and confirmed the specificity of the interactions between the growth factors and **17**.

**Figure 2:**
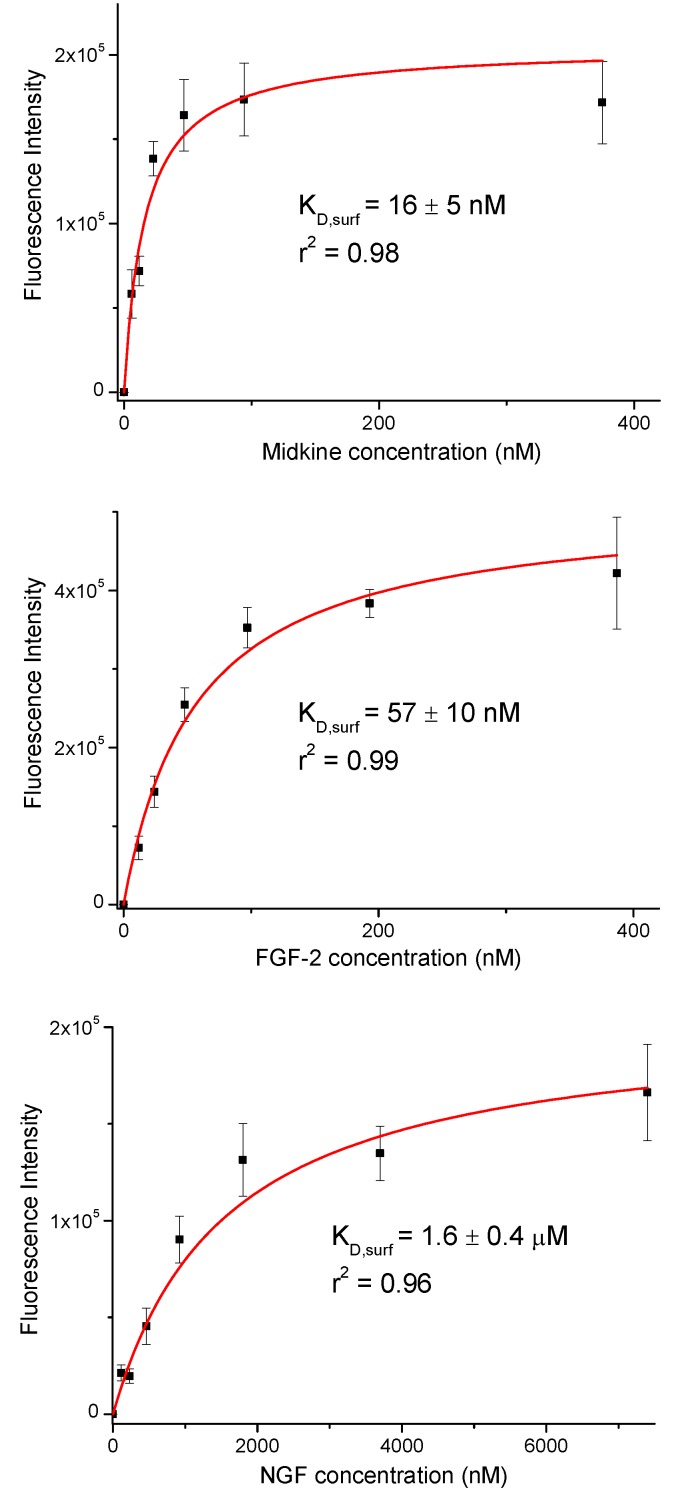
Binding curves for the interactions between immobilized hexasaccharide **17** and midkine, FGF-2 and NGF. The displayed fluorescence intensity values are the averages of at least three replicate wells and the error bars represent the standard deviations.

Fluorescence intensities were plotted against growth factor concentration (Figure 2). We observed a concentration dependent increase in the fluorescence signal. The resulting curves were fitted to the equation for a one-site binding model and surface dissociation constants (K_D,surf_) were obtained (Figure 2 and Table 1) [42,43]. The K_D,surf_ values for the interaction between immobilized **17** and midkine/FGF-2 were 16 and 57 nM, respectively, confirming the strong carbohydrate-protein binding. A K_D,surf_ value of 1.6 µM was determined for the **17**/NGF interaction, indicating that the hexasaccharide recognizes NGF with less affinity. The obtained dissociation constants followed the same trend as literature data for GAG-growth factor interactions (GAG-midkine/FGF-2 and GAG-NGF bindings in the nano- and micromolar ranges, respectively) [3,9]. On the other hand, these results also showed clear differences between the relative binding affinities obtained from the FP experiment and the sugar-coated surface assay (Table 1). This fact can be easily explained by the differences in the two experimental setups. In the FP competition method, we measured the binding of hexasaccharide **17** and the protein in solution, while in the sugar immobilized experiment, we observed the direct interaction between the growth factor and a multivalent functionalized surface. As previously shown, the format of the interaction experiment can strongly influence the calculated affinity values [4,43,44]. Thus, the multivalent presentation of the fluorinated oligosaccharide on the microplate provided much higher binding affinities, in comparison with the solution-phase results.

## 3. Materials and Methods

### 3.1. General Synthetic Procedures

Thin-layer chromatography (TLC) analyses were performed on silica gel 60F-254 precoated aluminium plates from Merck (Darmstadt, Germany). The compounds were detected by UV visualization (λ = 254 nm) and by staining with 5% *v*/*v* anisaldehyde-5% *v*/*v* H_2_SO_4_-0.2% *v*/*v* AcOH in EtOH or 0.2% *w*/*v* cerium (IV) sufate-5% *w*/*v* ammonium molybdate tetrahydrate in 1 M H_2_SO_4_ followed by heating at over 200 °C. Column chromatography was carried out on silica gel 60 (0.2–0.063 mm or 0.040–0.015 mm; Merck). Optical rotations were determined with a Perkin-Elmer 341 polarimeter (Perkin Elmer, Waltham, MA, USA). ^1^H- and ^13^C-NMR spectra were acquired on an Avance III-400 spectrometer (Bruker, Billerica, MA, USA). 2-D COSY and HSQC experiments were routinely carried out to assist in signal assignment. Unit A refers to the reducing end monosaccharide in the NMR data. Electrospray mass spectra (ESI MS) were carried out with an Esquire 6000 ESI-Ion Trap from Bruker Daltonics (Billerica, MA, USA). High resolution mass spectra (HR MS) were carried out by CITIUS (Universidad de Sevilla, Sevilla, Spain). Microwave-based sulfation reactions were performed using a Biotage Initiator Eight synthesizer in sealed reaction vessels (Biotage, Uppsala, Sweden). Compound **4** was purchased from Carbosynth. FluoroFlash silica gel was purchased from Sigma-Aldrich (Merck, Darmstadt, Germany).

### 3.2. General Procedure for F-SPE

We followed our previously reported experimental procedure [25,30,45]. Briefly, FluoroFlash silica gel (5 g., Fluorous Technologies, Inc., Ambridge, PA, USA) was placed in a glass chromatography column (1.7 cm diameter). The F-SPE column was washed with DMF (2 mL) and then preconditioned with MeOH/H_2_O 80:20 (15 mL). Next, the crude sample (50–300 mg) was dissolved in DMF/H_2_O 9:1 (0.8 mL) and loaded on the column. The fluorophobic elution was carried out with 15 mL of MeOH/H_2_O 80:20. The fluorous compounds were then eluted using 100% acetone (20 mL). To regenerate the F-SPE column, we washed with additional acetone (20 mL). The initial DMF washing step can be omitted when reusing the F-SPE column.

### 3.3. Immobilization of Fluorous-Tagged Sugar on Microtiter Plates and Determination of Surface Dissociation Constants (K_D, surf_)

A 1 mM solution of 2,2,3,3,4,4,5,5,6,6,7,7,8,8,9,9,9-heptadecafluorononylamine (C_8_F_17_CH_2_NH_2_) in DMSO was placed in the wells of Nunc Immobilizer Amino^TM^ 384 microtiter plates (from Thermo Scientific, Waltham, MA, USA) (20 µL/well). The plate was shaken overnight at room temperature and the wells were emptied and washed twice with water. Then, the wells were incubated with 100 mM ethanolamine in sodium bicarbonate buffer (50 mM, pH 9.6) (80 µL/well). After shaking for 1 h, the wells were washed twice with water. Next, fluorous-coated wells were filled with a 250 µM solution of **17** in water/DMSO 9:1 (20 µL/well). After 6 h, the plate was washed with water and was ready to perform protein interaction studies. Blank samples were wells not incubated with **17**, and coated with the fluorous linker alone.

Recombinant human FGF-2, midkine and NGF (from Peprotech) were reconstituted in PBS buffer (10 mM, pH 7.4) containing 1% BSA. Wells were incubated with 20 µL of protein solutions at different concentrations. All samples were performed at least in three replicates. After shaking at room temperature for 1 h, the microplate was washed with PBS containing 1% Tween 20 and 0.1% BSA, and water. Rabbit anti-human FGF-2, midkine and NGF antibodies (from Peprotech) were reconstituted in PBS containing 1% BSA. 20 µL of primary antibody solution at 5 µg/mL concentration was added to the wells in order to detect the bound protein. The plate was shaken for 1 h and washed with PBS containing 1% Tween 20 and 0.1% BSA, and water. Finally, the primary rabbit antibodies were detected by using Alexa Fluor 488 labelled anti-rabbit IgG (from Invitrogen, 20 µg/mL in PBS containing 1% BSA, 20 µL/well). The plate was incubated with shaking in the dark for 1 h and then washed with PBS containing 1% Tween 20 and 0.1% BSA, and water.

The fluorescence was read at 535 nm using a TRIAD multimode microplate reader (from Dynex). Blank wells, coated only with heptadecafluorononylamine, and not treated with **17**, were also incubated with each protein and the corresponding antibodies. The residual fluorescence intensities obtained from these blank samples were subtracted from all values. The average fluorescence intensity values of at least three replicates were plotted against protein concentration. The resulting curve was fitted to the equation for a one-site binding model: y = F_max_x/(x + K_D,surf_) where F_max_ is the maximum fluorescence intensity approached with increasing protein concentrations and K_D,surf_ is the dissociation constant of the surface sugar/protein interaction.

### 3.4. Procedure for Synthesis of Hexasaccharide ***17***

#### 3.4.1. Synthesis of 4-Methoxyphenyl 2-deoxy-3-*O*-levulinoyl-2-phthalimido-β-d-glucopyranoside (**5**)

Lev_2_O preparation: LevOH (3.48 mL, 33.4 mmol) was added at 0 °C to a solution of 1,3-dicyclohexylcarbodiimide (3.48 g, 16.7 mmol) in CH_2_Cl_2_ (32 mL). After stirring for 5 min at room temperature, the mixture was cooled and filtered, and the urea precipitate was washed with additional CH_2_Cl_2_ (6 mL) to give 38 mL of a 0.44 M Lev_2_O solution.

Lev_2_O (38 mL of a 0.44 M solution in CH_2_Cl_2_) was added at room temperature to a mixture of **4** (2.8 g, 5.6 mmol) and DMAP (103 mg, 0.834 mmol). The mixture was stirred for 1.5 h, diluted with CH_2_Cl_2_, and washed with saturated aqueous NaHCO_3_, and brine. The organic phase was dried (MgSO_4_), filtered and concentrated to dryness. The residue was suspended in hexane/EtOAc (1:1, 8 mL) and the mixture was filtered. The solid was washed with additional hexane/EtOAc to give the 3-*O*-levulinated monosaccharide as a white amorphous solid (3.21 g, 96%) that was used without further purification. TLC (toluene-EtOAc 4:1) Rf 0.33.

TFA (6.2 mL) and H_2_O (0.93 mL) were added to a solution of the levulinated compound (3.21 g, 5.34 mmol) in CH_2_Cl_2_ (62 mL) at 0 °C. The solution was stirred for 2 h at room temperature, then it was diluted with CH_2_Cl_2_, and washed with saturated NaHCO_3_ aqueous solution and brine. The organic layer was dried (MgSO_4_), filtered, and concentrated in vacuo. The residue was purified by column chromatography (toluene/EtOAc 1:1 → toluene/EtOAc 1:3) to give **5** (2.5 g, 91%) as a white foam. TLC (toluene/EtOAc 1:2): Rf = 0.15. [α]^20^_D_ + 35° (*c* 1.0, CHCl_3_); ^1^H-NMR (400 MHz, CDCl_3_): δ 7.85 (m, 2H, Ar), 7.73 (m, 2H, Ar), 6.84 (m, 2H, Ar), 6.74 (m, 2H, Ar), 5.92 (d, 1H, *J*_1,2_ = 8.4 Hz, H-1), 5.78 (dd, 1H, *J*_2,3_ = 10.8 Hz, *J*_3,4_ = 8.9 Hz, H-3), 4.48 (dd, 1H, H-2), 4.01 (m, 1H, H-6a), 3.93–3.87 (m, 2H, H-6b, H-4), 3.77–3.72 (m, 4H, H-5, Me (OMP)), 3.52 (d, 1H, *J*_4,OH_ = 4.0 Hz, OH), 2.68–2.33 (m, 4H, CH_2_(Lev)), 2.24 (br t, 1H, OH), 2.03 (s, 3H, CH_3_(Lev)); ^13^C-NMR (100 MHz, CDCl_3_): δ 207.5, 172.9, 168.0 (CO), 155.5–114.6 (Ar), 97.2 (C-1), 75.8 (C-5), 73.7 (C-3), 69.8 (C-4), 62.1 (C-6), 55.6 (Me (OMP)), 54.5 (C-2), 38.0 (CH_2_(Lev)), 29.5 (CH_3_(Lev)), 28.0 (CH_2_(Lev)); HR MS: *m*/*z*: calcd for C_26_H_27_NO_10_Na: 536.1527; found: 536.1529 [*M* + Na]^+^.

#### 3.4.2. Synthesis of 4-Methoxyphenyl 4,6-*O*-di-tert-butylsilylidene-2-deoxy-3-*O*-levulinoyl-2-phthalimido-β-d-glucopyranoside (**6**)

Compound **5** (2.0 g, 3.89 mmol) was dissolved in dry Py (60 mL) and cooled (0 °C). di-*tert*-Butylsilyl *bis*(trifluoromethanesulfonate) (1.57 mL, 4.67 mmol) was added and the mixture was stirred at room temperature for 1.5 h. The reaction was quenched with MeOH (17 mL), diluted with EtOAc (300 mL), and washed with 1 M HCl, saturated aqueous NaHCO_3_, and brine. The organic phase was dried (MgSO_4_), filtered and concentrated to dryness. The residue was purified by column chromatography (toluene-EtOAc 10:1) to afford **6** as a white foam (2.37 g, 93%). TLC (toluene-EtOAc 5:1) Rf 0.43; [α]^20^_D_ + 22° (*c* 1.0, CHCl_3_); ^1^H-NMR (400 MHz, CDCl_3_): δ 7.86 (m, 2H, Ar), 7.73 (m, 2H, Ar), 6.82 (m, 2H, Ar), 6.73 (m, 2H, Ar), 5.90 (d, 1H, *J*_1,2_ = 8.4 Hz, H-1), 5.75 (dd, 1H, *J*_2,3_ = 10.8 Hz, *J*_3,4_ = 9.0 Hz, H-3), 4.48 (dd, 1H, H-2), 4.25 (dd, 1H, *J*_5,6a_ = 5.1 Hz, *J*_6a,6b_ = 10.3 Hz, H-6a), 4.03 (t, 1H, *J*_4,5_ = 9.4 Hz, H-4), 4.00 (t, 1H, *J*_5,6b_ = 10.3 Hz, H-6b), 3.74 (td, 1H, H-5), 3.71 (s, 3H, Me (OMP)), 2.64–2.41 (m, 4H, CH_2_(Lev)), 1.96 (s, 3H, CH_3_(Lev)), 1.06, 0.99 (2s, 18H, Si(C(CH_3_)_3_)_2_); ^13^C-NMR (100 MHz, CDCl_3_): δ 205.7, 172.0, 168.3, 167.7 (4 × CO), 155.8-114.6 (Ar), 97.9 (C-1), 75.6 (C-4), 72.5 (C-3), 70.8 (C-5), 66.3 (C-6), 55.6 (Me (OMP)), 54.6 (C-2), 37.9 (CH_2_(Lev)), 29.5 (CH_3_(Lev)), 28.0 (CH_2_(Lev)), 27.5, 27.0 (Si(C(*C*H_3_)_3_)_2_), 22.8, 20.0 (Si(*C*(CH_3_)_3_)_2_); HR MS: *m*/*z*: calcd for C_34_H_43_NO_10_SiNa: 676.2548; found: 676.2555 [*M* + Na]^+^.

#### 3.4.3. Synthesis of 4,6-*O*-di-*tert*-Butylsilylidene-2-deoxy-3-*O*-levulinoyl-2-phthalimido-α,β-d-glucopyranose (**7**)

CAN (20 mL of a 0.73 M solution in H_2_O) was added to a cooled solution of **6** (2.37 g, 3.62 mmol) in CH_2_Cl_2_/MeCN (1:2; 180 mL). After stirring for 50 min at 0 °C, the reaction mixture was diluted with EtOAc, washed with H_2_O, saturated aqueous NaHCO_3_, and brine. The organic phase was dried (MgSO_4_), filtered and concentrated to dryness. The residue was purified by column chromatography (toluene-EtOAc 4:1) to afford **7** as light-yellow foam (1.81 g, 91%, mixture of α/β anomers). TLC (toluene/EtOAc 4:1) Rf 0.23; ^1^H-NMR (400 MHz, CDCl_3_) (data for β anomer): δ 7.85 (m, 2H, Ar), 7.72 (m, 2H, Ar), 5.74 (dd, 1H, *J*_2,3_ = 10.7 Hz, *J*_3,4_ = 8.9 Hz, H-3), 5.62 (br t, 1H, H-1), 4.25 (dd, 1H, *J*_5,6a_ = 5.0 Hz, *J*_6a,6b_ = 10.3 Hz, H-6a), 4.17 (dd, 1H, *J*_1,2_ = 8.4 Hz, H-2), 3.99–3.94 (m, 2H, H-4, H-6b), 3.72 (td, 1H, *J*_4,5_ = *J*_5,6b_ = 9.9 Hz, H-5), 3.24 (d, 1H, *J*_1,OH_ = 7.6 Hz, OH), 2.63–2.39 (m, 4H, CH_2_(Lev)), 1.95 (s, 3H, CH_3_(Lev)), 1.05, 0.99 (2s, 18H, Si(C(CH_3_)_3_)_2_); ^13^C-NMR (100 MHz, CDCl_3_) (data for β anomer): δ 206.0, 172.0, 168.3, 168.1 (4 × CO), 134.6–123.6 (Ar), 93.1 (C-1), 75.8 (C-4), 72.4 (C-3), 70.8 (C-5), 66.3 (C-6), 56.2 (C-2), 37.9 (CH_2_(Lev)), 29.5 (CH_3_(Lev)), 28.0 (CH_2_(Lev)), 27.4, 27.0 (Si(C(*C*H_3_)_3_)_2_), 22.7, 20.0 (Si(*C*(CH_3_)_3_)_2_); HR MS: *m*/*z*: calcd for C_27_H_37_NO_9_SiNa: 570.2130; found: 570.2132 [*M* + Na]^+^.

#### 3.4.4. Synthesis of *O*-(4,6-*O*-di-*tert*-Butylsilylidene-2-deoxy-3-*O*-levulinoyl-2-phthalimido-α,β-d-glucopyranosyl) trichloroacetimidate (**2**)

Trichloroacetonitrile (1.95 mL, 19.2 mmol) and catalytic DBU (19 µL, 0.13 mmol) were added to a solution of **7** (700 mg, 1.28 mmol) in dry CH_2_Cl_2_ (13 mL) under an argon atmosphere. After stirring for 4 h at room temperature, the reaction mixture was concentrated to dryness. The residue was purified by a short silica gel column (toluene-EtOAc 8:1 + 1% Et_3_N) to afford **2** as a white foam (659 mg, 75%, mixture of α/β anomers). TLC (toluene-EtOAc 7:1) Rf 0.41; ^1^H-NMR (400 MHz, CDCl_3_) (data for β anomer): δ 8.62 (s, 1H, NH), 7.83 (m, 2H, Ar), 7.71 (m, 2H, Ar), 6.65 (d, 1H, *J*_1,2_ = 8.8 Hz, H-1), 5.81 (dd, 1H, *J*_2,3_ = 10.8 Hz, *J*_3,4_ = 8.9 Hz, H-3), 4.54 (dd, 1H, H-2), 4.32 (dd, 1H, *J*_5,6a_ = 5.0 Hz, *J*_6a,6b_ = 10.1 Hz, H-6a), 4.05 (t, 1H, H-4), 4.01 (t, 1H, *J*_5,6b_ = 10.1 Hz, H-6b), 3.87 (td, 1H, *J*_4,5_ = 9.8 Hz, H-5), 2.65–2.41 (m, 4H, CH_2_(Lev)), 1.95 (s, 3H, CH_3_(Lev)), 1.05, 1.00 (2s, 18H, Si(C(CH_3_)_3_)_2_); ^13^C-NMR (100 MHz, CDCl_3_) (data for β anomer): δ 205.6, 171.8, 167.6 (4 × CO), 160.7 (C=NH), 134.2-123.6 (Ar), 93.9 (C-1), 90.2 (CCl_3_), 75.4 (C-4), 72.0 (C-3), 71.4 (C-5), 66.2 (C-6), 53.6 (C-2), 37.9 (CH_2_(Lev)), 29.5 (CH_3_(Lev)), 27.9 (CH_2_(Lev)), 27.4, 26.9 (Si(C(*C*H_3_)_3_)_2_), 22.7, 20.0 (Si(*C*(CH_3_)_3_)_2_); ESI MS: *m*/*z*: calcd for C_29_H_37_Cl_3_N_2_O_9_SiNa: 713.1; found: 713.1 [*M* + Na]^+^.

#### 3.4.5. Synthesis of 4-Methoxyphenyl *O*-(4,6-*O*-di-*tert*-butylsilylidene-2-deoxy-3-*O*-levulinoyl-2-phthalimido-β-d-glucopyranosyl)-(1→4)-*O*-(2-*O*-benzoyl-3-*O*-benzyl-6-*O*-pivaloyl-β-d-glucopyranosyl)-(1→3)-*O*-(4,6-*O*-di-*tert*-butylsilylidene-2-deoxy-2-phthalimido-β-d-glucopyranosyl)-(1→4)-*O*-(2-*O*-benzoyl-3-*O*-benzyl-6-*O*-pivaloyl-β-d-glucopyranosyl)-(1→3)-*O*-(4,6-*O*-di-*tert*-butylsilylidene-2-deoxy-2-phthalimido-β-d-glucopyranosyl)-(1→4)-2-*O*-benzoyl-3-*O*-benzyl-6-*O*-4,4,5,5,6,6,7,7,8,8,9,9,10,10,11,11,11-heptadecafluoroundecanoyl-β-d-glucopyranoside (**16**)

Donor **2** (55 mg, 0.08 mmol) and acceptor **3** (38 mg, 0.04 mmol) were dissolved in dry CH_2_Cl_2_ (1.1 mL) in the presence of activated 4Å molecular sieves (MS, 90 mg). The reaction mixture was stirred, under an argon atmosphere, for 10 min at 0 °C and TBSOTf (65 µL of a 0.085 M solution in dry CH_2_Cl_2_) was added. After stirring for 25 min at 0 °C, the reaction mixture was quenched with triethylamine, filtered, and then concentrated under reduced pressure. The crude product was purified using a fluorous solid-phase extraction (F-SPE) column to give disaccharide **8** as a white foam (59 mg). TLC (hexane-EtOAc 2:1) Rf 0.35; ^1^H-NMR (400 MHz, CDCl_3_): δ 7.98 (m, 2H, Ar), 7.85 (m, 2H, Ar), 7.73 (m, 2H, Ar), 7.57 (m, 1H, Ar), 7.43 (m, 2H, Ar), 7.28-7.17 (m, 5H, Ar), 6.79 (m, 2H, Ar), 6.68 (m, 2H, Ar), 5.62 (m, 2H, H-3′, H-1′), 5.41 (t, 1H, *J*_2,3_ = 6.8 Hz, H-2), 5.00 (d, 1H, *J*_1,2_ = 6.6 Hz, H-1), 4.77 (m, 2H, CH_2_(Bn)), 4.43 (dd, 1H, *J*_5,6a_ = 2.1 Hz, *J*_6a,6b_ = 11.8 Hz, H-6a), 4.21 (dd, 1H, *J*_1,2_ = 8.4 Hz, *J*_2,3_ = 10.7 Hz, H-2′), 4.03 (dd, 1H, *J*_3,4_ = 8.0 Hz, *J*_4,5_ = 9.4 Hz, H-4), 3.92 (dd, 1H, *J*_5,6a_ = 5.0 Hz, *J*_6a,6b_ = 10.2 Hz, H-6′a), 3.86 (br t, 1H, H-3), 3.82 (t, 1H, *J*_3,4_ = *J*_4,5_ = 9.3 Hz, H-4′), 3.77 (dd, 1H, *J*_5,6b_ = 4.5 Hz, H-6b), 3.68–3.63 (m, 4H, H-5, Me (OMP)), 3.60 (t, 1H, *J*_5,6b_ = 10.2 Hz, H-6’b), 3.42 (td, 1H, H-5’), 2.63-2.08 (m, 8H, -CH_2_-CH_2_-, CH_2_(Lev)), 1.92 (s, 3H, CH_3_(Lev)), 0.99, 0.89 (2s, 18H, Si(C(CH_3_)_3_)_2_); ^13^C-NMR (100 MHz, CDCl_3_, selected data from HSQC experiment): δ 99.4 (C-1), 98.5 (C-1’), 80.5 (C-3), 76.7 (C-4), 75.1 (C-4’), 73.4 (CH_2_(Bn)), 72.2 (C-2), 72.1 (C-5), 72.0 (C-3’), 70.3 (C-5’), 65.6 (C-6’), 62.3 (C-6), 55.0 (C-2’, Me (OMP)); ESI MS: *m*/*z*: calcd for C_65_H_66_F_17_NO_17_SiNa: 1506.4; found: 1506.3 [*M* + Na]^+^.

Compound **8** (59 mg, 0.04 mmol) was dissolved in CH_2_Cl_2_ (1.0 mL) and hydrazine monohydrate (160 µL of a 0.5 M solution in Py/AcOH 3:2) was added. After stirring at room temperature for 1 h, the reaction mixture was quenched with acetone (0.2 mL). The mixture was diluted with CH_2_Cl_2_ and washed with 1 M HCl aqueous solution, saturated NaHCO_3_ aqueous solution and H_2_O. The organic layer was dried (MgSO_4_), filtered and concentrated in vacuo. The crude product was purified using a F-SPE column to give disaccharide **9** as a white foam (51 mg). TLC (hexane-EtOAc 2:1) Rf 0.54; ^1^H-NMR (400 MHz, CDCl_3_): δ 7.99–7.73 (m, 6H, Ar), 7.57 (m, 1H, Ar), 7.43 (m, 2H, Ar), 7.28–7.16 (m, 5H, Ar), 6.79 (m, 2H, Ar), 6.68 (m, 2H, Ar), 5.50 (d, 1H, *J*_1,2_ = 8.3 Hz, H-1′), 5.41 (br t, 1H, H-2), 5.00 (d, 1H, *J*_1,2_ = 6.6 Hz, H-1), 4.77 (m, 2H, CH_2_(Bn)), 4.45 (dd, 1H, *J*_5,6a_ = 2.1 Hz, *J*_6a,6b_ = 11.8 Hz, H-6a), 4.30 (m, 1H, H-3′), 4.17 (dd, 1H, *J*_2,3_ = 10.8 Hz, H-2′), 4.03 (dd, 1H, *J*_3,4_ = 8.0 Hz, *J*_4,5_ = 9.4 Hz, H-4), 3.93 (dd, 1H, *J*_5,6a_ = 5.1 Hz, *J*_6a,6b_ = 10.2 Hz, H-6′a), 3.87 (t, 1H, *J*_2,3_ = 7.4 Hz, H-3), 3.82 (dd, 1H, *J*_5,6b_ = 4.7 Hz, H-6b), 3.68–3.63 (m, 5H, H-5, H-4′, Me (OMP)), 3.61 (t, 1H, *J*_5,6b_ = 10.2 Hz, H-6′b), 3.35 (td, 1H, *J*_4,5_ = 9.8 Hz, H-5′), 2.51 (br d, 1H, OH), 2.29–2.12 (m, 4H, -CH_2_-CH_2_-), 1.01, 0.92 (2s, 18H, Si(C(CH_3_)_3_)_2_); ^13^C-NMR (100 MHz, CDCl_3_, selected data from HSQC experiment): δ 99.5 (C-1), 98.8 (C-1′), 80.6 (C-3), 77.9 (C-4′), 76.8 (C-4), 73.5 (CH_2_(Bn)), 72.5 (C-2, C-5), 71.0 (C-3′), 70.3 (C-5′), 65.9 (C-6′), 62.7 (C-6), 56.3 (C-2′), 55.3 (Me (OMP)); ESI MS: *m*/*z*: calcd for C_60_H_60_F_17_NO_15_SiNa: 1408.3; found: 1408.1 [*M* + Na]^+^.

Donor **1** (65 mg, 0.093 mmol) and acceptor **9** (51 mg, 0.037 mmol) were dissolved in dry CH_2_Cl_2_ (1.0 mL) in the presence of activated 4Å MS (90 mg). The reaction mixture was stirred, under an argon atmosphere, for 10 min at rt and TBSOTf (143 µL of a 0.13 M solution in dry CH_2_Cl_2_) was added. After stirring for 25 min at rt, the reaction mixture was quenched with triethylamine, filtered, and then concentrated under reduced pressure. The crude product was purified using a F-SPE column to give a fluorophilic fraction (70 mg) containing trisaccharide **10** as main product together with a small amount (<10%) of unreacted acceptor **9** that was capped by standard acetylation. Data for trisaccharide **10:** TLC (hexane-EtOAc 2:1) Rf 0.27; ^1^H-NMR (400 MHz, CDCl_3_): δ 7.95 (m, 2H, Ar), 7.72–7.54 (m, 8H, Ar), 7.44–7.36 (m, 4H, Ar), 7.24–6.92 (m, 10H, Ar), 6.74 (m, 2H, Ar), 6.65 (m, 2H, Ar), 5.36 (t, 1H, *J*_1,2_ = *J*_2,3_ = 6.7 Hz, H-2A), 5.27 (d, 1H, *J*_1,2_ = 8.4 Hz, H-1B), 5.10 (t, 1H, *J*_3,4_ = *J*_4,5_ = 9.4 Hz, H-4C), 4.99–4.94 (m, 3H, H-1C, H-2C, H-1A), 4.71 (m, 2H, CH_2_(Bn)), 4.54 (dd, 1H, *J*_2,3_ = 10.5, *J*_3,4_ = 8.6 Hz, H-3B), 4.34 (m, 2H, CH_2_(Bn)), 4.28 (dd, 1H, *J*_5,6a_ = 2.0 Hz, *J*_6a,6b_ = 11.8 Hz, H-6aA), 4.23 (dd, 1H, H-2B), 4.20 (dd, 1H, *J*_5,6a_ = 2.1 Hz, *J*_6a,6b_ = 12.2 Hz, H-6aC), 4.14 (dd, 1H, *J*_5,6b_ = 4.6 Hz, H-6bC), 3.97–3.90 (m, 3H, H-4B, H-4A, H-6aB), 3.78 (t, 1H, *J*_3,4_ = 7.5 Hz, H-3A), 3.69-3.51 (m, 8H, Me (OMP), H-6bA, H-6bB, H-3C, H-5C, H-5A), 3.35 (td, 1H, *J*_5,6_ = 4.9 Hz, *J*_5,6_ = *J*_4,5_ = 9.7 Hz, H-5B), 2.65-2.13 (m, 8H, -CH_2_-CH_2_-, CH_2_(Lev)), 2.11 (s, 3H, CH_3_(Lev)), 1.23 (s, 9H, C(CH_3_)_3_), 1.05, 0.95 (2s, 18H, Si(C(CH_3_)_3_)_2_); ^13^C-NMR (100 MHz, CDCl_3_, selected data from HSQC experiment): δ 99.3 (C-1C, C-1A), 98.7 (C-1B), 80.4 (C-3A), 80.1 (C-3C), 76.7 (C-3B), 76.6 (C-4B, C-4A), 74.2 (C-2C), 73.4 (2 × CH_2_(Bn)), 72.5 (C-5A, C-5C), 72.4 (C-2A), 70.4 (C-5B), 69.3 (C-4C), 65.9 (C-6B), 62.5 (C-6A), 62.2 (C-6C), 55.8 (C-2B), 55.3 (Me (OMP)); ESI MS: *m*/*z*: calcd for C_90_H_94_F_17_NO_24_SiNa: 1946.6; found: 1946.5 [*M* + Na]^+^.

Compound **10** (70 mg, 0.036 mmol) was dissolved in CH_2_Cl_2_ (1.0 mL) and hydrazine monohydrate (144 µL of a 0.5 M solution in Py/AcOH 3:2) was added. After stirring at room temperature for 45 min, the reaction mixture was quenched with acetone (0.2 mL). The mixture was diluted with CH_2_Cl_2_ and washed with 1 M HCl aqueous solution, saturated NaHCO_3_ aqueous solution and H_2_O. The organic layer was dried (MgSO_4_), filtered and concentrated in vacuo. The crude product was purified by F-SPE to give trisaccharide **11** as a white amorphous solid (63 mg). TLC (hexane-EtOAc 2:1) Rf 0.39; ^1^H-NMR (400 MHz, CDCl_3_): δ 7.95 (m, 2H, Ar), 7.71–7.54 (m, 8H, Ar), 7.44–7.36 (m, 4H, Ar), 7.24–6.97 (m, 10H, Ar), 6.74 (m, 2H, Ar), 6.65 (m, 2H, Ar), 5.36 (t, 1H, *J*_1,2_ = *J*_2,3_ = 6.8 Hz, H-2A), 5.28 (d, 1H, *J*_1,2_ = 8.5 Hz, H-1B), 4.97 (d, 1H, *J*_1,2_ = 7.6 Hz, H-1C), 4.94 (d, 1H, *J*_1,2_ = 6.6 Hz, H-1A), 4.90 (br t, 1H, H-2C), 4.71 (m, 2H, CH_2_(Bn)), 4.55–4.50 (m, 3H, CH_2_(Bn), H-3B, H-6aC), 4.41 (m, 1H, CH_2_(Bn)), 4.29–4.21 (m, 3H, H-6aA, H-6bC, H-2B), 4.00-3.90 (m, 3H, H-4B, H-4A, H-6aB), 3.77 (t, 1H, *J*_3,4_ = 7.5 Hz, H-3A), 3.67–3.57 (m, 5H, Me (OMP)), H-6bA, H-6bB), 3.53 (m, 1H, H-5A), 3.49 (m, 1H, H-4C), 3.44–3.32 (m, 3H, H-3C, H-5C, H-5B), 2.95 (br s, 1H, OH), 2.23–2.02 (m, 4H, -CH_2_-CH_2_-), 1.24 (s, 9H, C(CH_3_)_3_), 1.06, 0.96 (2s, 18H, Si(C(CH_3_)_3_)_2_); ^13^C-NMR (100 MHz, CDCl_3_, selected data from HSQC experiment): δ 99.4 (C-1C, C-1A), 98.8 (C-1B), 82.2 (C-3C), 80.5 (C-3A), 77.0 (C-3B), 76.8 (C-4B, C-4A), 74.6 (CH_2_(Bn), C-5C), 74.4 (C-2C), 73.4 (CH_2_(Bn)), 72.4 (C-2A, C-5A), 70.4 (C-5B), 69.9 (C-4C), 65.9 (C-6B), 63.2 (C-6C), 62.6 (C-6A), 56.0 (C-2B), 55.4 (Me (OMP)); ESI MS: *m*/*z*: calcd for C_85_H_88_F_17_NO_22_SiNa: 1848.5; found: 1848.2 [*M* + Na]^+^.

Donor **2** (67 mg, 0.097 mmol) and acceptor **11** (63 mg, 0.034 mmol) were dissolved in dry CH_2_Cl_2_ (1.0 mL) in the presence of activated 4Å MS (75 mg). The reaction mixture was stirred, under an argon atmosphere, for 10 min at rt and TBSOTf (114 µL of a 0.085 M solution in dry CH_2_Cl_2_) was added. After stirring for 40 min at rt, the reaction mixture was quenched with triethylamine, filtered, and then concentrated under reduced pressure. The crude product was purified by F-SPE to give a fluorophilic fraction (77 mg) containing tetrasaccharide **12** as main product. A small amount (<10%) of unreacted acceptor **11** was also detected and capped by standard acetylation. Data for tetrasaccharide **12:** TLC (hexane-EtOAc 2:1) Rf 0.22; ^1^H-NMR (400 MHz, CDCl_3_): δ 7.95–7.33 (m, 18H, Ar), 7.20–6.98 (m, 10H, Ar), 6.73 (m, 2H, Ar), 6.64 (m, 2H, Ar), 5.59 (dd, 1H, *J*_2,3_ = 10.6 Hz, *J*_3,4_ = 9.0 Hz, H-3D), 5.35–5.32 (m, 2H, H-2A, H-1D), 5.22 (d, 1H, *J*_1,2_ = 8.5 Hz, H-1B), 4.92 (d, 1H, *J*_1,2_ = 6.6 Hz, H-1A), 4.85 (t, 1H, *J*_2,3_ = 7.9 Hz, H-2C), 4.72 (d, 1H, *J*_1,2_ = 7.2 Hz, H-1C), 4.68 (m, 2H, CH_2_(Bn)), 4.62 (m, 1H, CH_2_(Bn)), 4.38 (dd, 1H, *J*_2,3_ = 10.5 Hz, *J*_3,4_ = 8.5 Hz, H-3B), 4.34 (m, 1H, CH_2_(Bn)), 4.29–4.22 (m, 2H, H-6aC, H-6aA), 4.16–4.09 (m, 2H, H-2B, H-2D), 3.90–3.81 (m, 5H, H-4A, H-4B, H-4C, H-6aB, H-6aD), 3.75 (t, 1H, *J*_3,4_ = 7.5 Hz, H-3A), 3.69–3.66 (m, 4H, H-4D, Me (OMP)), 3.63–3.56 (m, 2H, H-6bA, H-6bC), 3.53–3.46 (m, 3H, H-6bB, H-3C, H-5A), 3.40 (td, 1H, *J*_5,6_ = 5.0 Hz, *J*_5,6_ = *J*_4,5_ = 9.9 Hz, H-5D), 3.31–3.23 (m, 3H, H-5B, H-6bD, H-5C), 2.60–1.99 (m, 8H, -CH_2_-CH_2_-, CH_2_(Lev)), 1.92 (s, 3H, CH_3_(Lev)), 1.17 (s, 9H, C(CH_3_)_3_), 0.95, 0.91, 0.90, 0.86 (4s, 36H, Si(C(CH_3_)_3_)_2_); ^13^C-NMR (100 MHz, CDCl_3_, selected data from HSQC experiment): δ 99.4 (C-1C), 99.3 (C-1A), 98.4 (C-1B), 97.9 (C-1D), 80.2 (C-3A), 79.6 (C-3C), 77.3 (C-3B), 76.3 (C-4A, C-4B, C-4C), 75.4 (C-4D), 73.9 (C-2C), 73.8 (CH_2_(Bn)), 73.6 (C-5C), 73.3 (CH_2_(Bn)), 72.1 (C-3D, C-2A, C-5A), 70.2 (C-5B), 70.1 (C-5D), 65.5 (C-6B, C-6D), 62.3 (C-6A, C-6C), 55.1 (C-2B, C-2D, Me (OMP)); ESI MS: *m*/*z*: calcd for C_112_H_123_F_17_N_2_O_30_Si_2_Na: 2377.7; found: 2377.4 [*M* + Na]^+^.

Compound **12** (77 mg, 0.033 mmol) was dissolved in CH_2_Cl_2_ (1.0 mL) and hydrazine monohydrate (132 µL of a 0.5 M solution in Py/AcOH 3:2) was added. After stirring at room temperature for 50 min, the reaction mixture was quenched with acetone (0.2 mL). The mixture was diluted with CH_2_Cl_2_ and washed with 1 M HCl aqueous solution, saturated NaHCO_3_ aqueous solution and H_2_O. The organic layer was dried (MgSO_4_), filtered and concentrated in vacuo. The crude product was purified by F-SPE to give tetrasaccharide **13** as a white amorphous solid (64 mg). TLC (hexane-EtOAc 2:1) Rf 0.43; ^1^H-NMR (400 MHz, CDCl_3_): δ 7.95–7.74 (m, 6H, Ar), 7.64–7.33 (m, 12H, Ar), 7.21–6.97 (m, 10H, Ar), 6.73 (m, 2H, Ar), 6.64 (m, 2H, Ar), 5.33 (t, 1H, *J*_2,3_ = 6.9 Hz, H-2A), 5.22 (d, 1H, *J*_1,2_ = 8.5 Hz, H-1B), 5.21 (d, 1H, *J*_1,2_ = 8.3 Hz, H-1D), 4.91 (d, 1H, *J*_1,2_ = 6.6 Hz, H-1A), 4.86 (br t, 1H, H-2C), 4.72 (d, 1H, *J*_1,2_ = 7.3 Hz, H-1C), 4.68 (m, 2H, CH_2_(Bn)), 4.63 (m, 1H, CH_2_(Bn)), 4.39 (dd, 1H, *J*_2,3_ = 10.5 Hz, *J*_3,4_ = 8.4 Hz, H-3B), 4.34 (m, 1H, CH_2_(Bn)), 4.30–4.22 (m, 3H, H-6aC, H-3D, H-6aA), 4.13 (dd, 1H, H-2B), 4.08 (dd, 1H, *J*_2,3_ = 10.8 Hz, H-2D), 3.90–3.81 (m, 5H, H-4A, H-4B, H-4C, H-6aB, H-6aD), 3.75 (m, 1H, H-3A), 3.66–3.58 (m, 5H, Me (OMP), H-6bA, H-6bC), 3.56–3.46 (m, 4H, H-6bB, H-5A, H-4D, H-3C), 3.37–3.23 (m, 4H, H-5D, H-6bD, H-5B, H-5C), 2.46 (br s, 1H, OH), 2.22–1.99 (m, 4H, -CH_2_-CH_2_-), 1.17 (s, 9H, C(CH_3_)_3_), 0.97, 0.94, 0.91, 0.86 (4s, 36H, Si(C(CH_3_)_3_)_2_); ^13^C-NMR (100 MHz, CDCl_3_, selected data from HSQC experiment): *δ* 99.5 (C-1C), 99.3 (C-1A), 98.2 (C-1B, C-1D), 80.4 (C-3A), 80.1 (C-3C), 78.0 (C-4D), 77.2 (C-3B), 76.3 (C-4A, C-4B, C-4C), 74.0 (C-2C), 73.9 (CH_2_(Bn)), 73.7 (C-5C), 73.5 (CH_2_(Bn)), 72.5 (C-2A), 72.3 (C-5A), 71.3 (C-3D), 70.7 (C-5B), 70.1 (C-5D), 65.7 (C-6B, C-6D), 62.5 (C-6A, C-6C), 56.2 (C-2D), 55.8 (C-2B), 55.3 (Me (OMP)); ESI MS: *m*/*z*: calcd for C_107_H_117_F_17_N_2_O_28_Si_2_Na: 2279.7; found: 2279.4 [*M* + Na]^+^.

Donor **1** (60 mg, 0.085 mmol) and acceptor **13** (64 mg, 0.028 mmol) were dissolved in dry CH_2_Cl_2_ (1.2 mL) in the presence of activated 4Å MS (90 mg). The reaction mixture was stirred, under an argon atmosphere, for 10 min at rt and TBSOTf (132 µL of a 0.13 M solution in dry CH_2_Cl_2_) was added. After stirring for 25 min at rt, the reaction mixture was quenched with triethylamine, filtered, and then concentrated under reduced pressure. The crude product was purified by F-SPE to give pentasaccharide **14** (75 mg) as the main product. A significant amount (around 25%) of unreacted acceptor **13** was also detected in the fluorous fraction. For this reason, we ran a second glycosylation cycle with additional donor **1** (38 mg, 0.054 mmol). F-SPE purification then gave a fluorous fraction (71 mg) containing pentasaccharide **14** as the main product and a small amount (<10%) of unreacted acceptor **13** that was capped by standard acetylation. Data for pentasaccharide **14:** TLC (hexane-EtOAc 3:2) Rf 0.43; ^1^H-NMR (400 MHz, CDCl_3_): δ 7.94–7.30 (m, 23H, Ar), 7.19–6.91 (m, 15H, Ar), 6.74–6.62 (m, 4H, Ar), 5.32 (t, 1H, *J*_1,2_ = *J*_2,3_ = 6.8 Hz, H-2A), 5.19 (d, 1H, *J*_1,2_ = 8.5 Hz, H-1B or D), 5.10 (t, 1H, *J*_3,4_ = *J*_4,5_ = 9.4 Hz, H-4E), 5.00 (br t, 1H, H-2E), 4.98 (d, 1H, *J*_1,2_ = 8.3 Hz, H-1B or D), 4.90 (d, 1H, *J*_1,2_ = 6.6 Hz, H-1A), 4.84 (d, 1H, *J*_1,2_ = 7.4 Hz, H-1E), 4.80 (br t, 1H, H-2C), 4.67–4.64 (m, 3H, CH_2_(Bn), H-1C), 4.57 (d, 1H, CH_2_(Bn)), 4.51 (dd, 1H, *J*_2,3_ = 10.4 Hz, *J*_3,4_ = 8.5 Hz, H-3B or D), 4.38-4.29 (m, 3H, CH_2_(Bn), H-3B or D), 4.25–4.20 (m, 2H, CH_2_(Bn), H-6aA), 4.16–4.06 (m, 5H, H-6aE, H-6bE, H-6aC, H-2B, H-2D), 3.88–3.76 (m, 6H, H-6aB, H-6aD, H-4A, H-4B, H-4C, H-4D), 3.73 (t, 1H, *J*_3,4_ = 7.4 Hz, H-3A), 3.65 (s, 3H, Me (OMP)), 3.63–3.57 (m, 2H, H-3E, H-6bA), 3.55–3.45 (m, 3H, H-5E, H-6bB or D, H-5A), 3.37–3.22 (m, 5H, H-6bC, H-6bB or D, H-3C, H-5B, H-5D), 3.03 (m, 1H, H-5C), 2.64-2.11 (m, 8H, -CH_2_-CH_2_-, CH_2_(Lev)), 2.10 (s, 3H, CH_3_(Lev)), 1.21, 1.15 (2s, 18H, C(CH_3_)_3_), 1.01, 0.98, 0.83, 0.82 (4s, 36H, Si(C(CH_3_)_3_)_2_); ^13^C-NMR (100 MHz, CDCl_3_, selected data from HSQC experiment): δ 99.8 (C-1E), 99.6 (C-1C), 99.4 (C-1A), 98.6, 97.6 (C-1B, C-1D), 80.5 (C-3C), 80.4 (C-3A), 80.3 (C-3E), 77.5, 77.4 (C-3B, C-3D), 77.4–75.7 (C-4A, C-4B, C-4C, C-4D), 74.5 (C-2E, CH_2_(Bn)), 74.1 (C-2C), 73.6 (2 × CH_2_(Bn)), 73.5 (C-5C), 72.8 (C-5E), 72.5 (C-2A, C-5A), 70.5 (C-5B, C-5D), 69.6 (C-4E), 66.0, 65.8 (C-6B, C-6D), 62.7 (C-6A), 62.4 (C-6C, C-6E), 55.9 (C-2B, C-2D), 55.6 (Me (OMP)); ESI MS: *m*/*z*: calcd for C_137_H_151_F_17_N_2_O_37_Si_2_Na: 2817.9; found: 2817.5 [*M* + Na]^+^.

Compound **14** (71 mg, 0.025 mmol) was dissolved in CH_2_Cl_2_ (1.0 mL) and hydrazine monohydrate (100 µL of a 0.5 M solution in Py/AcOH 3:2) was added. After stirring at room temperature for 1 h, the reaction mixture was quenched with acetone (0.15 mL). The mixture was diluted with CH_2_Cl_2_ and washed with 1 M HCl aqueous solution, saturated NaHCO_3_ aqueous solution and H_2_O. The organic layer was dried (MgSO_4_), filtered and concentrated in vacuo. The crude product was purified by F-SPE to give pentasaccharide **15** as a white amorphous solid (61 mg). TLC (hexane-EtOAc 3:2) Rf 0.35; ^1^H-NMR (400 MHz, CDCl_3_): δ 7.94-7.30 (m, 23H, Ar), 7.19–6.92 (m, 15H, Ar), 6.74–6.62 (m, 4H, Ar), 5.32 (t, 1H, *J*_1,2_ = *J*_2,3_ = 6.8 Hz, H-2A), 5.19 (d, 1H, *J*_1,2_ = 8.4 Hz, H-1B or D), 4.99 (d, 1H, *J*_1,2_ = 8.5 Hz, H-1B or D), 4.92 (br t, 1H, H-2E), 4.90 (d, 1H, *J*_1,2_ = 6.6 Hz, H-1A), 4.85 (d, 1H, *J*_1,2_ = 7.6 Hz, H-1E), 4.80 (br t, 1H, H-2C), 4.67–4.63 (m, 3H, CH_2_(Bn), H-1C), 4.59–4.47 (m, 4H, CH_2_(Bn), H-6aE, H-3B or D), 4.40 (d, 1H, CH_2_(Bn)), 4.33 (dd, 1H, *J*_2,3_ = 10.5 Hz, *J*_3,4_ = 8.4 Hz, H-3B or D), 4.25–4.17 (m, 3H, CH_2_(Bn), H-6aA, H-6bE), 4.12–4.07 (m, 3H, H-6aC, H-2B, H-2D), 3.88–3.78 (m, 6H, H-6aB, H-6aD, H-4A, H-4B, H-4C, H-4D), 3.73 (t, 1H, *J*_3,4_ = 7.4 Hz, H-3A), 3.65 (s, 3H, Me (OMP)), 3.59 (dd, 1H, *J*_5,6b_ = 4.6 Hz, *J*_6a,6b_ = 11.8 Hz, H-6bA), 3.52-3.44 (m, 3H, H-6bB or D, H-5A, H-4E), 3.40 (t, 1H, *J*_2,3_ = *J*_3,4_ = 8.8 Hz, H-3E), 3.37–3.22 (m, 6H, H-6bC, H-5E, H-3C, H-6bB or D, H-5B, H-5D), 3.03 (m, 1H, H-5C), 2.98 (br d, 1H, OH), 2.23–1.89 (m, 4H, -CH_2_-CH_2_-), 1.23, 1.15 (2s, 18H, C(CH_3_)_3_), 1.02, 0.99, 0.83 (4s, 36H, Si(C(CH_3_)_3_)_2_); ^13^C-NMR (100 MHz, CDCl_3_, selected data from HSQC experiment): δ 99.8 (C-1E), 99.5 (C-1C), 99.4 (C-1A), 98.5, 97.4 (C-1B, C-1D), 81.9 (C-3E), 80.3 (C-3A), 80.2 (C-3C), 77.4 (C-3B, C-3D), 77.4–75.4 (C-4A, C-4B, C-4C, C-4D), 74.6 (C-5E), 74.5 (CH_2_(Bn)), 74.4 (C-2E, CH_2_(Bn)), 74.0 (C-2C), 73.5 (CH_2_(Bn)), 73.3 (C-5C), 72.3 (C-5A), 72.2 (C-2A), 70.4 (C-5B, C-5D), 69.9 (C-4E), 65.9, 65.7 (C-6B, C-6D), 63.1 (C-6E), 62.6 (C-6A), 62.3 (C-6C), 55.7 (C-2B, C-2D), 55.4 (Me (OMP)); ESI MS: *m*/*z*: calcd for C_132_H_145_F_17_N_2_O_35_Si_2_Na: 2719.9; found: 2719.7 [*M* + Na]^+^.

Donor **2** (48 mg, 0.069 mmol) and acceptor **15** (61 mg, 0.023 mmol) were dissolved in dry CH_2_Cl_2_ (1.0 mL) in the presence of activated 4Å MS (75 mg). The reaction mixture was stirred, under an argon atmosphere, for 10 min at rt and TBSOTf (108 µL of a 0.064 M solution in dry CH_2_Cl_2_) was added. After stirring for 25 min at rt, the reaction mixture was quenched with triethylamine, filtered, and then concentrated under reduced pressure. The crude product was first purified by F-SPE. The fluorous fraction was then purified by standard silica gel column chromatography (hexane-EtOAc 2:1) to afford pure **16** (50 mg, 39% from **3**; 9 steps) as a white amorphous solid. TLC (toluene-EtOAc 4:1) Rf 0.59; [α]^20^_D_ −5° (*c* 1.0, CHCl_3_); ^1^H-NMR (400 MHz, CDCl_3_): δ 7.93-6.88 (m, 42H, Ar), 6.73–6.61 (m, 4H, Ar), 5.59 (dd, 1H, *J*_2,3_ = 10.6 Hz, *J*_3,4_ = 9.0 Hz, H-3F), 5.33–5.30 (m, 2H, H-1F, H-2A), 5.18 (d, 1H, *J*_1,2_ = 8.4 Hz, H-1B or D), 4.93–4.86 (m, 3H, H-1B or D, H-1A, H-2C or E), 4.77 (t, 1H, *J*_1,2_ = *J*_2,3_ = 8.1 Hz, H-2C or E), 4.66–4.52 (m, 6H, H-1C, H-1E, CH_2_(Bn)), 4.38–4.19 (m, 6H, H-3B, H-3D, CH_2_(Bn), H-6aA or C or E), 4.13–4.06 (m, 3H, H-2F, H-2B or D, H-6aA or C or E), 4.00 (dd, 1H, *J*_1,2_ = 8.4 Hz, *J*_2,3_ = 10.3 Hz, H-2B or D), 3.87–3.66 (m, 10H, H-6aB, H-6aD, H-6aF, H-4A, H-4C, H-4E, H-4F, H-4B, H-4D, H-3A), 3.65 (s, 3H, Me (OMP)), 3.60-3.53 (m, 2H, H-6bA or C or E), 3.51–3.37 (m, 4H, H-6bB or D or F, H-5A, H-3C or E, H-5B or D or F), 3.33-3.20 (m, 7H, H-6bA or C or E, H-3C or E, H-6bB or D or F, H-5B or D or F, H-5C or E), 2.99 (m, 1H, H-5C or E), 2.63–2.01 (m, 8H, -CH_2_-CH_2_-, CH_2_(Lev)), 1.92 (s, 3H, CH_3_(Lev)), 1.16, 1.13 (2s, 18H, C(CH_3_)_3_), 0.95, 0.91, 0.88, 0.81 (5s, 54H, Si(C(CH_3_)_3_)_2_); ^13^C-NMR (100 MHz, CDCl_3_): δ 205.7, 177.4, 177.2, 172.0, 170.2 (5 x CO), 168.5-167.0 (6 × CO), 165.1, 165.0, 164.9 (3 × CO), 155.5-114.4 (Ar), 100.1, 99.7 (C-1C, C-1E), 99.5 (C-1A), 98.6 (C-1B or D), 97.8 (C-1F), 97.5 (C-1B or D), 80.4, 80.3, 80.1 (C-3A, C-3C, C-3E), 77.8 (C-3B, C-3D), 77.0-75.3 (C-4A, C-4C, C-4E, C-4B, C-4D, C-4F), 74.5 (CH_2_(Bn)), 74.2, 74.1 (CH_2_(Bn), C-2C, C-2E), 73.8, 73.6, 73.5 (CH_2_(Bn), C-5C, C-5E), 72.5 (C-2A, C-5A, C-3F), 70.4 (C-5B, C-5D, C-5F), 65.9, 65.7 (C-6B, C-6D, C-6F), 62.7, 62.6, 62.4 (C-6A, C-6C, C-6E), 56.0, 55.9 (C-2B, C-2D), 55.5 (Me (OMP)), 55.2 (C-2F), 38.8 (*C*(CH_3_)_3_), 37.9 (CH_2_(Lev)), 29.6 (CH_3_(Lev)), 27.9 (CH_2_(Lev)), 27.4, 27.3, 27.1, 27.0, 26.9 (C(*C*H_3_)_3_, Si(C(*C*H_3_)_3_)_2_), 26.2 (t, *J*_C,F_ = 21.1 Hz, -*C*H_2_-CF_2_-), 24.7 (-CH_2_-), 22.6, 22.5, 20.0, 19.9, 19.8 (Si(*C*(CH_3_)_3_)_2_); HR MS: *m*/*z*: calcd for C_159_H_180_F_17_N_3_O_43_Si_3_Na: 3249.0919; found: 3249.0895 [*M* + Na]^+^.

#### 3.4.6. Synthesis of 4-Methoxyphenyl *O*-(2-deoxy-3-*O*-levulinoyl-2-phthalimido-4,6-di-*O*-sulfo-β-d-glucopyranosyl)-(1→4)-*O*-(2-*O*-benzoyl-3-*O*-benzyl-6-*O*-pivaloyl-β-d-glucopyranosyl)-(1→3)-*O*-(2-deoxy-2-phthalimido-4,6-di-*O*-sulfo-β-d-glucopyranosyl)-(1→4)-*O*-(2-*O*-benzoyl-3-*O*-benzyl-6-*O*-pivaloyl-β-d-glucopyranosyl)-(1→3)-*O*-(2-deoxy-2-phthalimido-4,6-di-*O*-sulfo-β-d-glucopyranosyl)-(1→4)-2-*O*-benzoyl-3-*O*-benzyl-6-*O*-4,4,5,5,6,6,7,7,8,8,9,9,10,10,11,11,11-heptadecafluoroundecanoyl-β-d-glucopyranoside (**17**)

An excess of (HF)_n_·Py (79 µL, 3.0 mmol) was added at 0 °C under an argon atmosphere to a solution of **16** (49 mg, 15 µmol) in dry THF (1.6 mL). After 18 h at 0 °C the mixture was diluted with CH_2_Cl_2_ and washed with H_2_O, saturated NaHCO_3_ solution and H_2_O. The organic layers were dried (MgSO_4_), filtered and concentrated in vacuo to give the corresponding hexaol (42 mg) as a white amorphous solid that was used without further purification. TLC (toluene-EtOAc 1:3) Rf 0.35; ESI MS: *m*/*z*: calcd for C_135_H_132_F_17_N_3_O_43_Cl: 2840.8; found: 2841.2 [*M* + Cl]^−^.

The desilylated compound (15 µmol) and sulfur trioxide–trimethylamine complex (125 mg, 0.9 mmol) were dissolved in dry DMF (4.0 mL) and heated at 100 °C for 30 min using microwave radiation (40 W average power). The reaction vessel was cooled and Et_3_N (350 µL), MeOH (2.0 mL) and CH_2_Cl_2_ (2.0 mL) were added. The solution was purified by Sephadex LH 20 chromatography (CH_2_Cl_2_-MeOH 1:1). Partially sulfated intermediates were still detected by TLC and, for this reason, the residue was submitted to a second and third sulfation cycle with new sulfating reagent using the same reaction conditions. After the third sulfation cycle, the reaction was completed and the crude mixture was purified by Sephadex LH 20 chromatography (CH_2_Cl_2_-MeOH 1:1) and silica gel column chromatography (EtOAc-MeOH-H_2_O 24:5:3 → EtOAc-MeOH-H_2_O 16:5:3). The residue was finally eluted from a Dowex 50WX2-Na^+^ column (MeOH as eluent) to obtain **17** as sodium salt (29 mg, 56%, 2 steps, white amorphous solid). TLC (EtOAc-MeOH-H_2_O 16:5:3) Rf 0.34; ^1^H-NMR (400 MHz, CD_3_OD): δ 7.96–6.86 (m, 42H, Ar), 6.78–6.66 (m, 4H, Ar), 5.83 (dd, 1H, *J*_2,3_ = 10.8 Hz, *J*_3,4_ = 8.8 Hz, H-3F), 5.44 (d, 1H, *J*_1,2_ = 8.3 Hz, H-1F), 5.33 (d, 1H, *J*_1,2_ = 8.3 Hz, H-1B or D), 5.25 (t, 1H, *J*_2,3_ = 8.3 Hz, H-2A), 5.16 (d, 1H, *J*_1,2_ = 7.7 Hz, H-1A), 5.12 (d, 1H, *J*_1,2_ = 8.4 Hz, H-1B or D), 4.90 (t, 1H, *J*_1,2_ = *J*_2,3_ = 8.3 Hz, H-2C or E), 4.83–4.78 (m, 2H, H-2C or E, CH_2_(Bn)), 4.76–4.54 (m, 7H, H-3B, H-3D, H-6aB, H-6aD, H-6aF, CH_2_(Bn), H-1C or E), 4.52–4.47 (m, 4H, H-1C or E, CH_2_(Bn)), 4.42 (d, 1H, CH_2_(Bn)), 4.36–4.22 (m, 5H, H-6aA, H-6aC or E, H-4F, H-4B, H-4D), 4.19–3.98 (m, 11H, H-2F, H-3A, H-2B, H-2D, H-6aC or E, H-6bA, H-6bB, H-6bD, H-6bF, H-5A, H-4C or E), 3.97–3.89 (m, 3H, H-4C or E, H-4A, H-5B or D), 3.83–3.73 (m, 3H, H-5B or D, H-5F, H-6bC or E), 3.65 (m, 4H, Me (OMP), H-6bC or E), 3.55 (t, 1H, *J*_2,3_ = *J*_3,4_ = 8.7 Hz, H-3C or E), 3.44–3.39 (m, 2H, H-3C or E, H-5C or E), 3.28 (m, 1H, H-5C or E), 2.59-2.32 (m, 8H, -CH_2_-CH_2_-, CH_2_(Lev)), 1.87 (s, 3H, CH_3_(Lev)), 1.19, 1.16 (2s, 18H, C(CH_3_)_3_); ^13^C-NMR (100 MHz, CD_3_OD): δ 208.8, 179.7, 179.6, 173.9, 172.2 (5 x CO), 169.9–168.8 (6 × CO), 167.2, 166.9 (3 × CO), 156.7–115.4 (Ar), 100.6, 100.5, 100.4 (C-1A C-1C, C-1E), 98.3 (C-1F), 98.0, 96.7 (C-1B, C-1D), 81.0, 80.8 (C-3C, C-3E), 78.9 (C-3A), 77.7, 77.4 (C-4B, C-4D), 77.1, 76.7, 76.4, 76.0 (C-4A, C-4C, C-4E, C-4F, C-3B, C-3D), 75.1 (C-2C, C-2E, C-5B, C-5D, C-5F, 2 x CH_2_(Bn)), 74.6 (C-2A), 74.3 (C-5C, C-5E), 73.9 (C-5A), 73.2 (CH_2_(Bn)), 71.8 (C-3F), 68.8, 68.3, 68.0 (C-6B, C-6D, C-6F), 64.5 (C-6A), 63.9 (C-6C, C-6E), 57.4, 57.3 (C-2B, C-2D), 56.4 (C-2F), 55.8 (Me (OMP)), 39.9, 39.8 (*C*(CH_3_)_3_), 38.5 (CH_2_(Lev)), 29.2 (CH_3_(Lev)), 29.1 (CH_2_(Lev)), 27.8 (C(*C*H_3_)_3_), 27.1 (t, *J*_C,F_ = 22.0 Hz, -*C*H_2_-CF_2_-), 25.9 (-CH_2_-); ESI MS: *m*/*z*: calcd for C_135_H_126_F_17_N_3_O_61_S_6_Na_3_^3−^: 1116.2; found: 1116.2 [*M* + 3Na]^3−^; HR MS: *m*/*z*: calcd for C_135_H_126_F_17_N_3_O_61_S_6_Na_4_^2−^: 1685.7252; found: 1685.7295 [*M* + 4Na]^2−^.

## 4. Conclusions

The synthesis of a hexasaccharide with the sequence GlcN(4,6-di-OSO_3_)-β(1→4)-Glc-β(1→3), related to the CS-E structure, was successfully accomplished. Starting monosaccharide building blocks **1**, **2** and **3** were efficiently assembled for the preparation of the target oligosaccharide and only two standard chromatographic purifications were required due to the assistance of a fluorinated tag. Using solution-phase FP competition experiments, we showed that the synthesized hexasaccharide **17** strongly interacted with midkine and FGF-2, although the IC_50_ values were slightly higher than those corresponding to the analogous tetramer. In addition, we also measured the direct binding of the growth factors to a microplate surface that was functionalized with the fluorous-tagged sugar. The calculated surface dissociation constants indicated a nanomolar interaction for midkine and FGF-2 (K_D,surf_ = 16 and 57 nM, respectively). On the contrary, NGF bound to the sugar-coated surface with less affinity (K_D,surf_ = 1.6 µM), suggesting that derivative **17** is more selective (≥28 fold) for midkine and FGF-2.

## Figures and Tables

**Table 1:** IC_50_ and K_D,surf_ values for the interaction between fluorinated sugar **17** and midkine, basic fibroblast growth factor (FGF-2) and nerve growth factor (NGF) proteins. The IC_50_ values were obtained from solution fluorescence polarization (FP) competition experiments while the K_D,surf_ values were calculated from assays using fluorous sugar-coated microplates.

	Midkine	FGF-2	NGF
IC_50_ (nM)	450 ± 30	6300 ± 1600	not determined
K_D,surf_ (nM)	16 ± 5	57 ± 10	1600 ± 400

## Supplementary Materials

The supplementary materials are available online.
