# Supplementary material for: Synthesis of a Fluorous-Tagged Hexasaccharide and Interaction with Growth Factors Using Sugar-Coated Microplates
Source: Molecules. 2019 Apr 22;24(8):1591. doi: 10.3390/molecules24081591 (PMC6515340; doi:10.3390/molecules24081591)
Supplement: Supplementary file 1 [file molecules-24-01591-s001.pdf]

Supplementary Materials

Synthesis of a fluorous-tagged hexasaccharide and interaction with growth factors using sugar coated microplates

Susana Maza, José L. de Paz, Pedro M. Nieto**

Instituto de Investigaciones Químicas (IIQ), Centro de Investigaciones Científicas Isla de La Cartuja, CSIC and Universidad de Sevilla, Américo Vespucio, 49, 41092 Sevilla, Spain.

	Page
**Fluorescence polarization assays**	S-3
**Figure S1**	S-4
**Figure S2**	S-5
**Interaction studies using sugar-coated microtiter plates**	
**Control experiments using additional (irrelevant) sugars (Figure S3)**	S-6
**Control experiments using additional (irrelevant) proteins (Figure S4)**	S-8
**NMR spectra**	
**Compound 5**	S-9
**Compound 6**	S-11
**Compound 7**	S-13
**Compound 2**	S-15
**Compound 8**	S-17

**Compound 9**	S-19
**Compound 10**	S-21
**Compound 11**	S-23
**Compound 12**	S-25
**Compound 13**	S-27
**Compound 14**	S-29
**Compound 15**	S-31
**Compound 16**	S-33
**Compound 17**	S-37

Fluorescence polarization assays

In order to calculate IC_{50} values, fluorescence polarization competition experiments were performed following our previously reported protocol.[1] Briefly, we recorded the fluorescence polarization from wells containing 20 μL of protein solution (midkine or FGF-2) and 10 μL of probe solution (a fluorescein labelled heparin-like hexasaccharide) in the presence of 10 μL of **17** solutions with different concentrations. 384-well microplates from Corning were used in these assays. After shaking in the dark for 5 min, the fluorescence polarization was measured using a TRIAD multimode microplate reader (from Dynex), with excitation and emission wavelengths of 485 and 535 nm, respectively. The average polarization values of three replicates were plotted against the logarithm of **17** concentration. The resulting curve was fitted to the equation for a one-site competition: $y = A_2 + (A_1-A_2)/[1+10^{(x-\log IC_{50})}]$ where A_1 and A_2 are the maximal and minimal values of polarization, respectively, and IC_{50} is the **17** concentration that results in 50% inhibition. Three independent experiments were carried out for each IC_{50} calculation.

[1] a) S. Maza, N. Gandia-Aguado, J. L. de Paz and P. M. Nieto, *Bioorg. Med. Chem.* **2018**, *26*, 1076-1085; b) J. L. de Paz, P. M. Nieto, *Org Biomol Chem.* **2016**, *14*, 3506-3509.

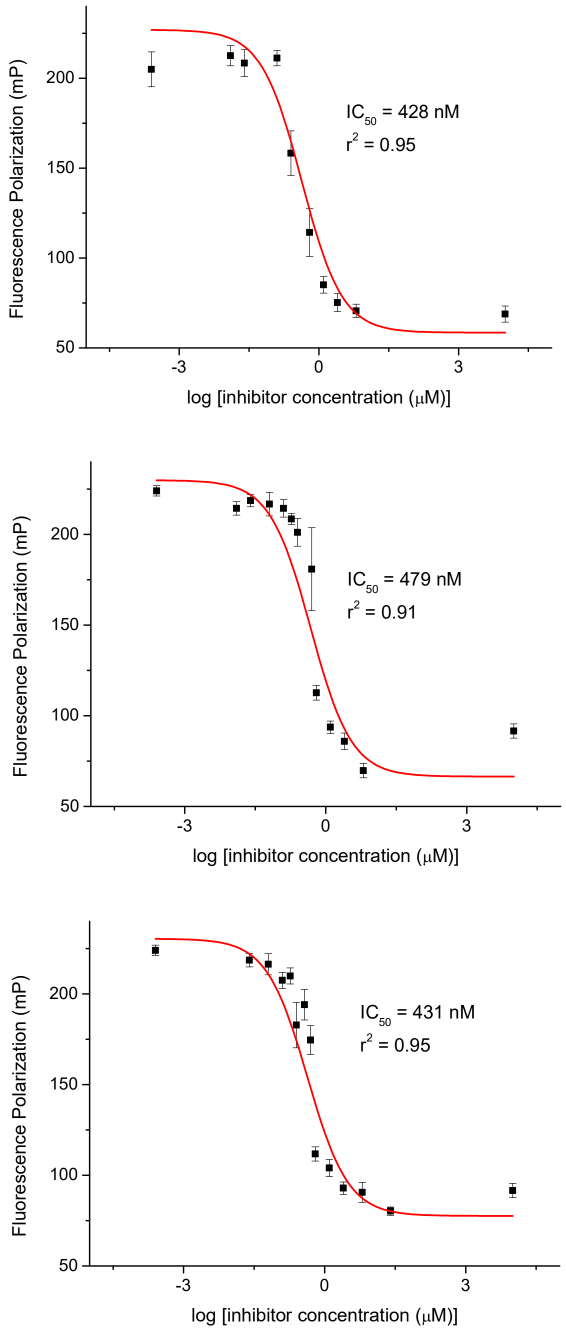

Figure S1. Competition curves displaying the ability of compound **17** to inhibit the interaction between midkine (63 nM) and fluorescent probe (10 nM). All the FP values are the average of three replicate wells, with error bars showing the standard deviations for these measurements. The reported IC_{50} value and the error (450 ± 30 nM, Table 1, main text) represent the average and the standard deviation from these three independent experiments.

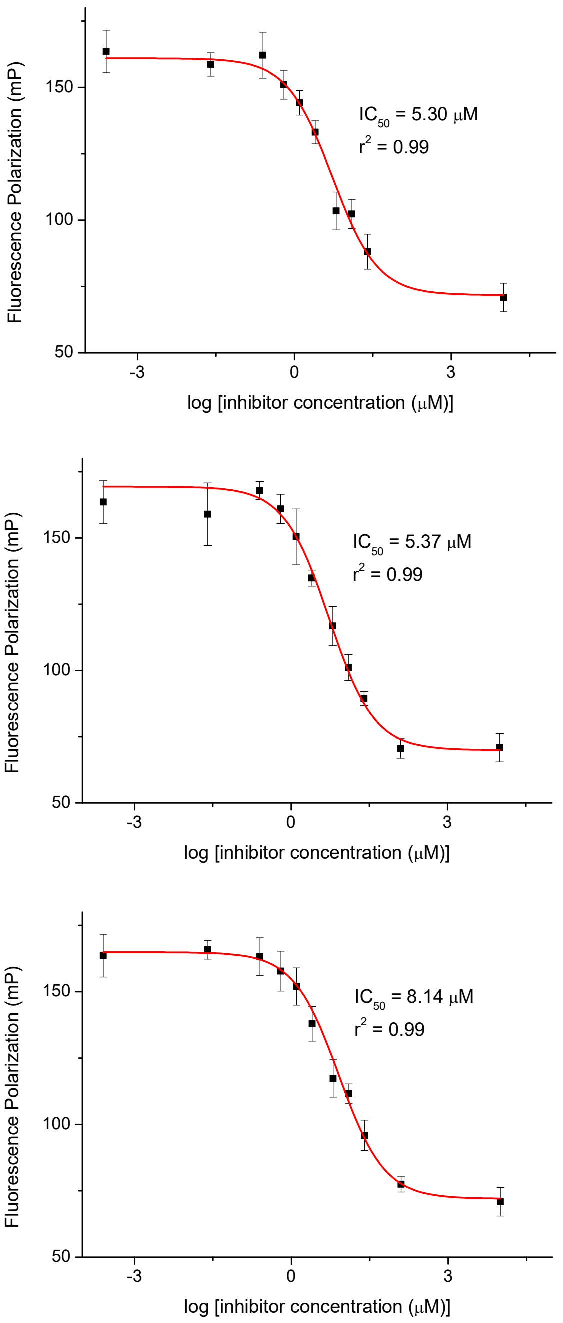

Figure S2. Competition curves displaying the ability of compound **17** to inhibit the interaction between FGF-2 (97 nM) and fluorescent probe (10 nM). All the FP values are the average of three replicate wells, with error bars showing the standard deviations for these measurements. The reported IC_{50} value and the error (6.3 ± 1.6 μM, Table 1, main text) represent the average and the standard deviation from these three independent experiments.

Interaction studies using sugar-coated microtiter plates.

1. Control experiments using additional (irrelevant) sugars.

The wells of Nunc Immobilizer Amino^{TM} 384 microtiter plates (from Thermo Scientific) were functionalized with heptadecafluorononylamine ($C_8F_{17}CH_2NH_2$) as described in the Materials and Methods section of the main text. The resulting fluorous linker-coated wells were filled with a 250 μM solution of monosaccharide **3** in DMSO (20 μL/well). For comparison purposes, we also included in this assay wells filled with a 250 μM solution of hexamer **17** in water/DMSO 9:1. All samples were performed in three replicates. Blank wells were not treated with any sugar. Thus, blank wells were coated with the fluorous linker alone. After shaking for 6 h, the plate was extensively washed with water and was ready to perform protein interaction studies.

Sugar-coated wells and blank wells were incubated with: a 375 nM solution of midkine in PBS buffer (10 mM, pH 7.4) containing 1% BSA; a 387 nM solution of FGF-2 in PBS buffer (10 mM, pH 7.4) containing 1% BSA; or a 1.8 μM solution of NGF in PBS buffer (10 mM, pH 7.4) containing 1% BSA (20 μL/well). After shaking at room temperature for 1 h, the microplate was extensively washed with PBS containing 1% Tween 20 and 0.1 % BSA, and water. Then, the microplate wells were incubated with the corresponding primary and secondary antibodies to detect any bound protein, as described in the Materials and Methods section of the main text.

Finally, the fluorescence was read at 535 nm using a TRIAD multimode microplate reader (from Dynex). For each protein, the residual fluorescence intensities obtained from blank samples were subtracted from the sugar-coated well values. The average fluorescence intensity values of three replicates are shown in Figure S3. Our results indicated that the three growth factors did not significantly interact with fluorous-tagged monosaccharide **3**.

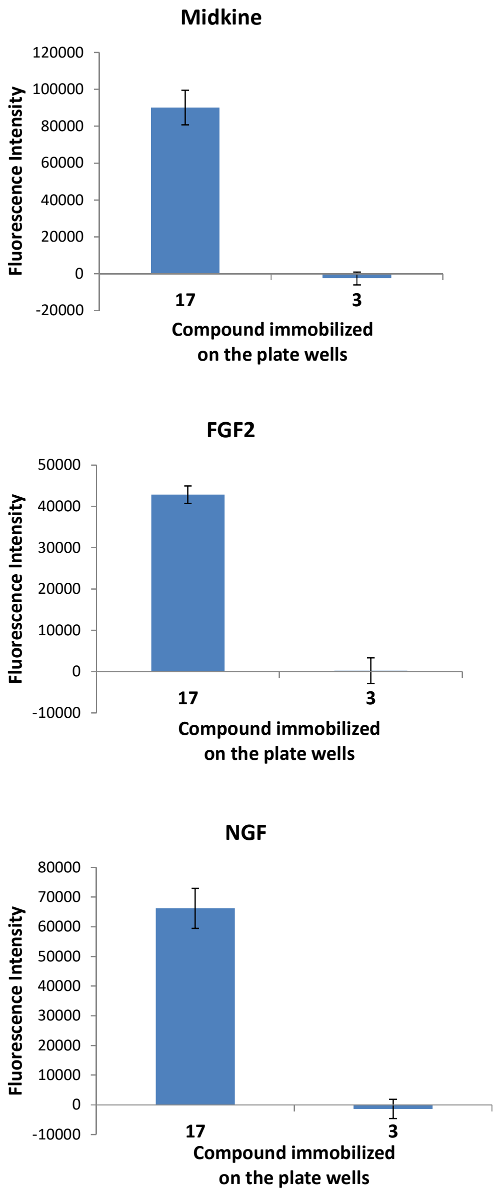

Figure S3. Fluorescence intensity values obtained from wells coated with **17** or **3** after incubation with midkine, FGF-2 and NGF. The displayed values are the average of three replicate wells and the error bars show the standard deviations for these measurements.

2. Control experiments using additional (irrelevant) proteins.

The wells of Nunc Immobilizer Amino™ 384 microtiter plates were functionalized with heptadecafluorononylamine linker and then filled with a solution of hexamer **17** as described in the Materials and Methods section of the main text. The resulting hexasaccharide **17**-coated wells were ready to perform protein interaction studies. Blank wells were coated with the fluorous linker alone.

On the other hand, bovine serum albumin (BSA, from Sigma-Aldrich) and concanavalin A (ConA, from Sigma) were fluorescently labelled with fluorescein isothiocyanate (FITC) following a standard protocol.[2] Next, **17**-coated wells and blank wells were both incubated with a 11 µM solution of FITC-labelled BSA or ConA in PBS buffer (10 mM, pH 7.4) containing 1% BSA (20 µL/well). All samples were performed in three replicates. After shaking in the dark at room temperature for 1 h, the microplate was extensively washed with PBS containing 1% Tween 20 and 0.1 % BSA, and water. Finally, the fluorescence was read at 535 nm using a TRIAD multimode microplate reader (from Dynex). For each protein, the residual fluorescence intensities obtained from blank samples were subtracted from the sugar-coated well values. In Figure S4, we show the average fluorescence intensities of three replicates after incubation with BSA and ConA. For comparison purposes, we also display the fluorescence values obtained after incubation with a 375 nM solution of midkine (detected by antibody incubations).

[2] G. T. Hermanson, *Bioconjugate Techniques*, 2nd Edition, Academic Press, Elsevier **2008**.

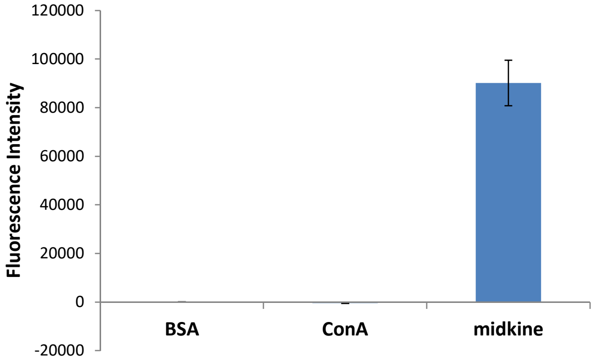

Figure S4. Fluorescence intensities from **17**-coated wells after incubation with BSA, ConA and midkine. The displayed data are the average of three replicates and the error bars show the standard deviations for these measurements.

Compound 5, ^{1}H-NMR

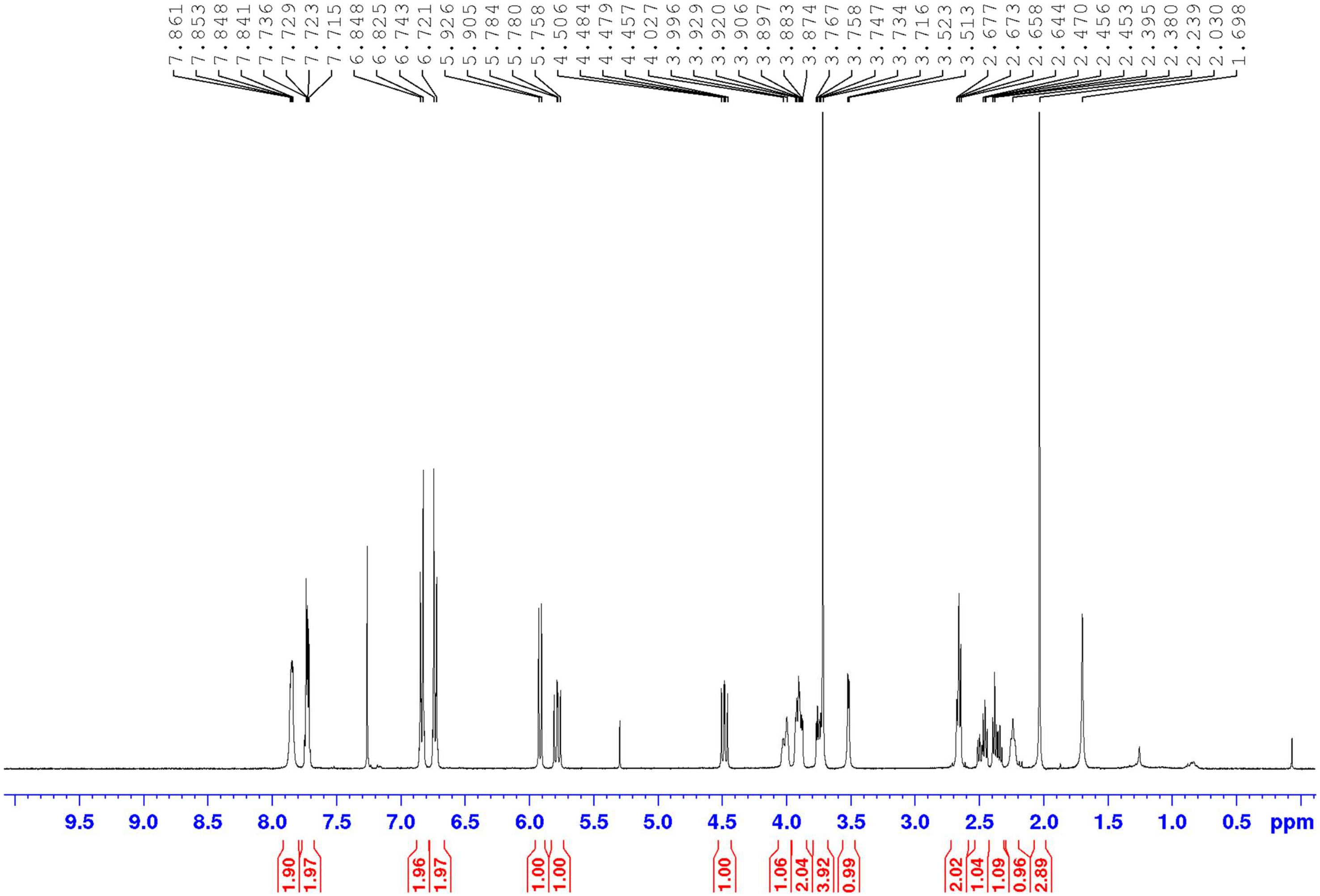

^{13}C-NMR

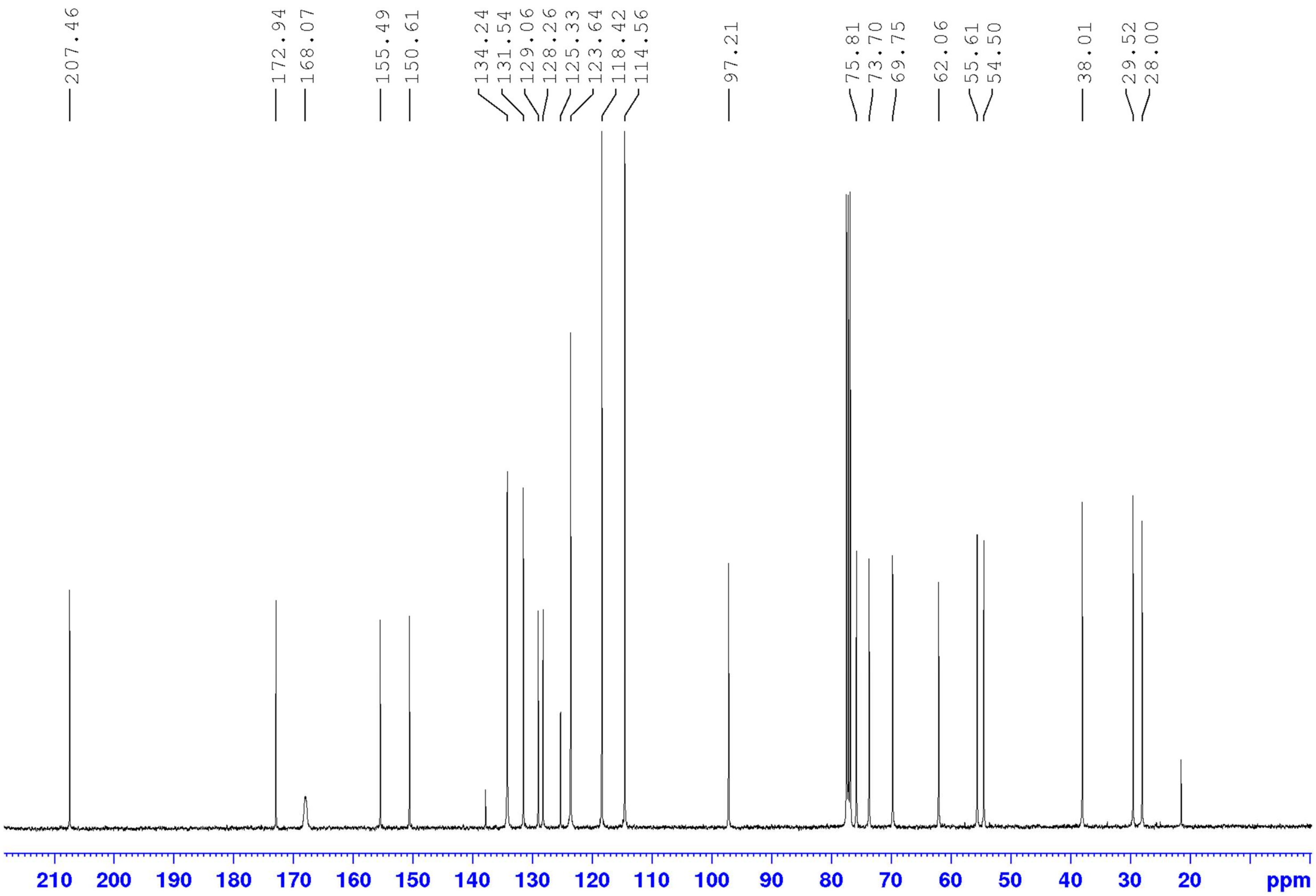
207.46
172.94
168.07
155.49
150.61
134.24
131.54
129.06
128.26
125.33
123.64
118.42
114.56
97.21
75.81
73.70
69.75
62.06
55.61
54.50
38.01
29.52
28.00
210 200 190 180 170 160 150 140 130 120 110 100 90 80 70 60 50 40 30 20 ppm

Compound 6, ^{1}H-NMR

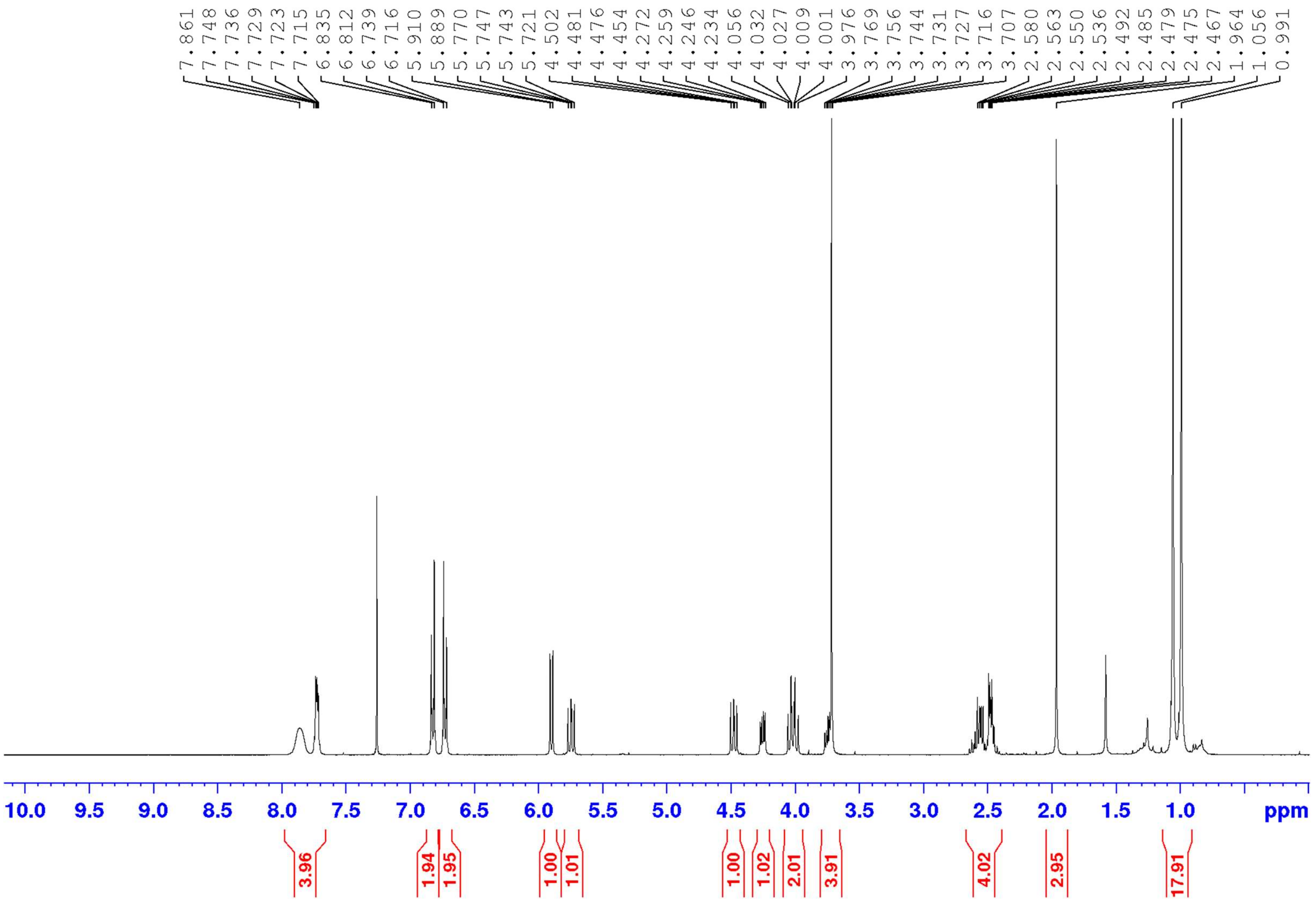

^{13}C-NMR

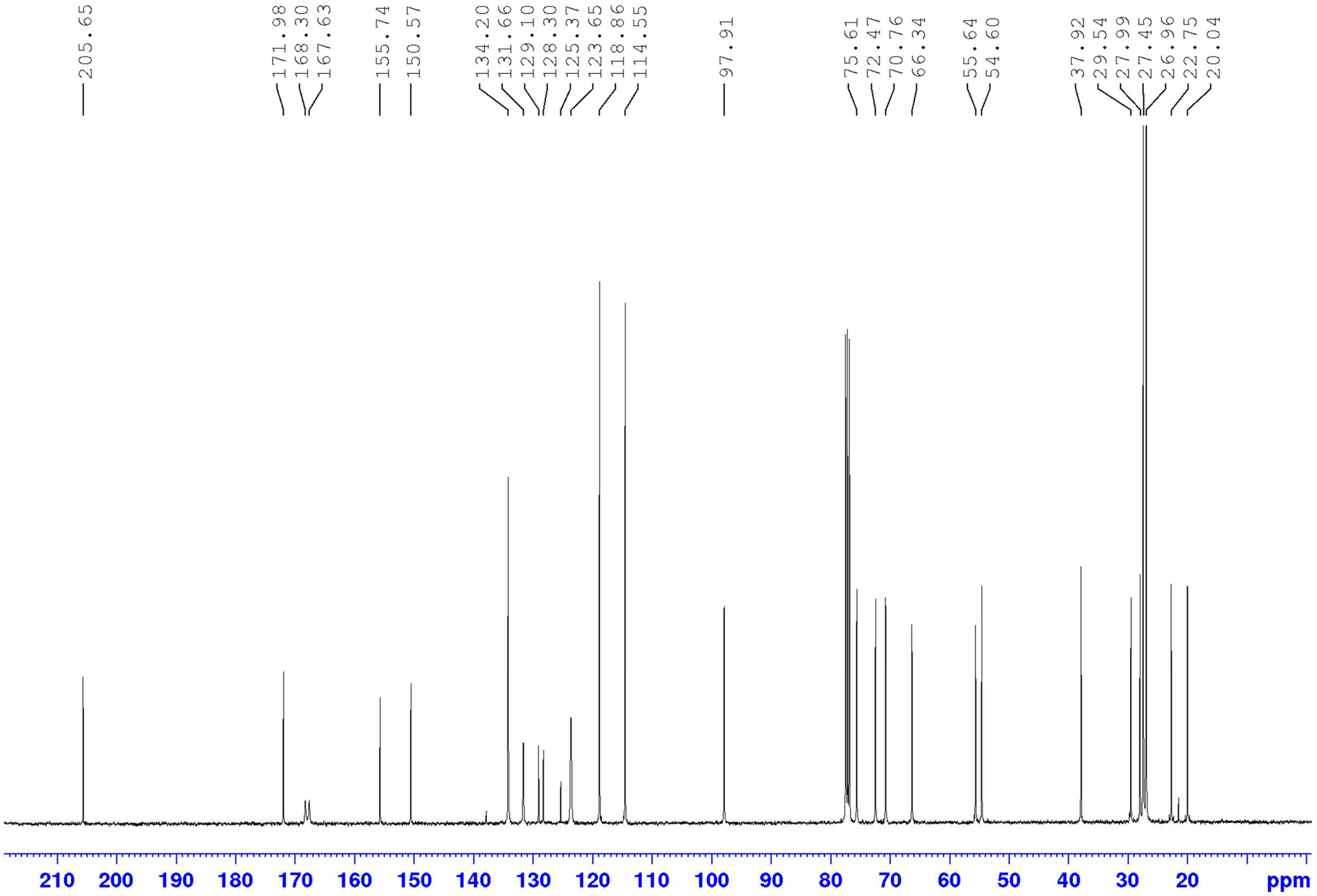
205.65
171.98
168.30
167.63
155.74
150.57
134.20
131.66
129.10
128.30
125.37
123.65
118.86
114.55
97.91
75.61
72.47
70.76
66.34
55.64
54.60
37.92
29.54
27.99
27.45
26.96
22.75
20.04
210 200 190 180 170 160 150 140 130 120 110 100 90 80 70 60 50 40 30 20
ppm

Compound **7**, ^{1}H-NMR

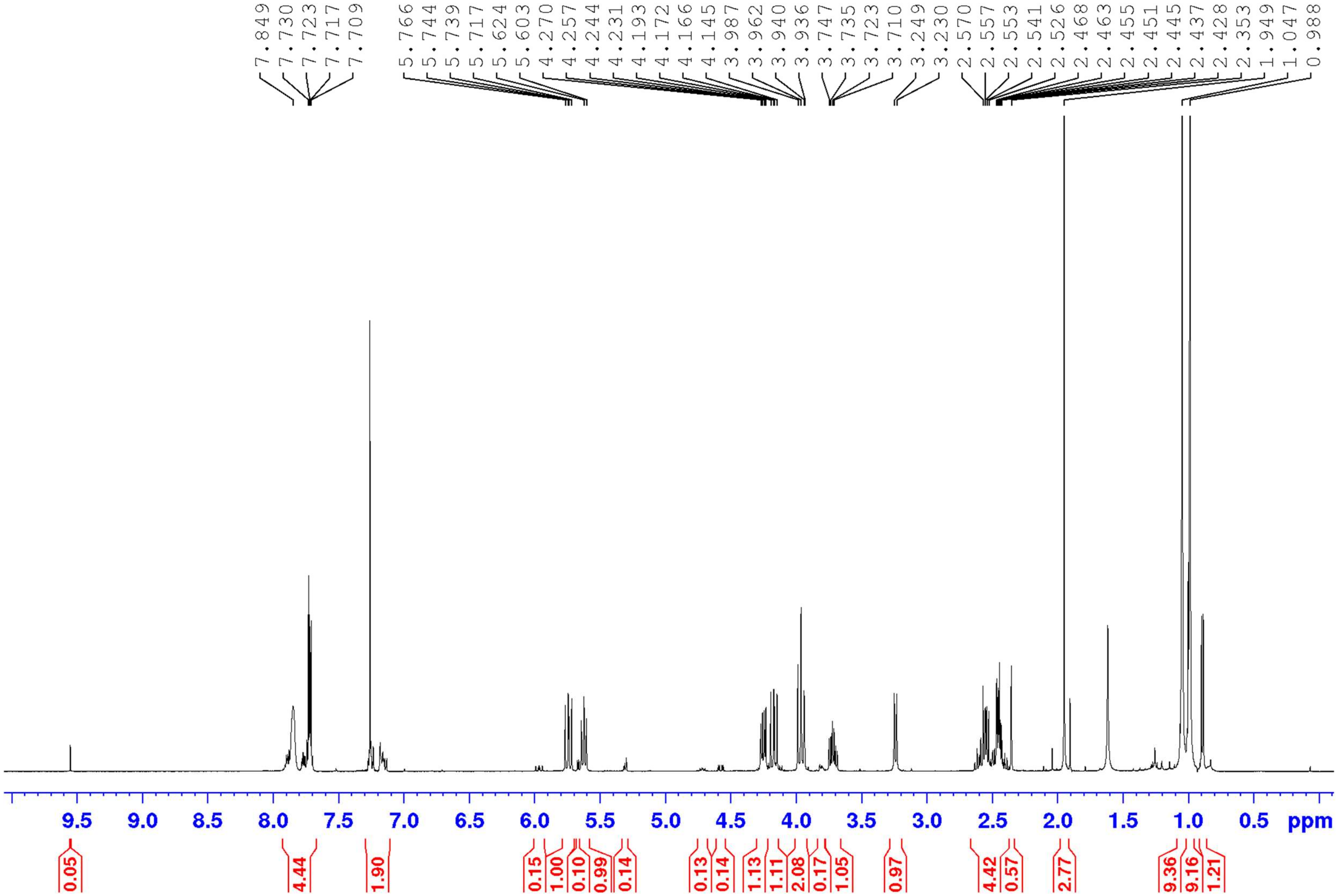

^{13}C-NMR

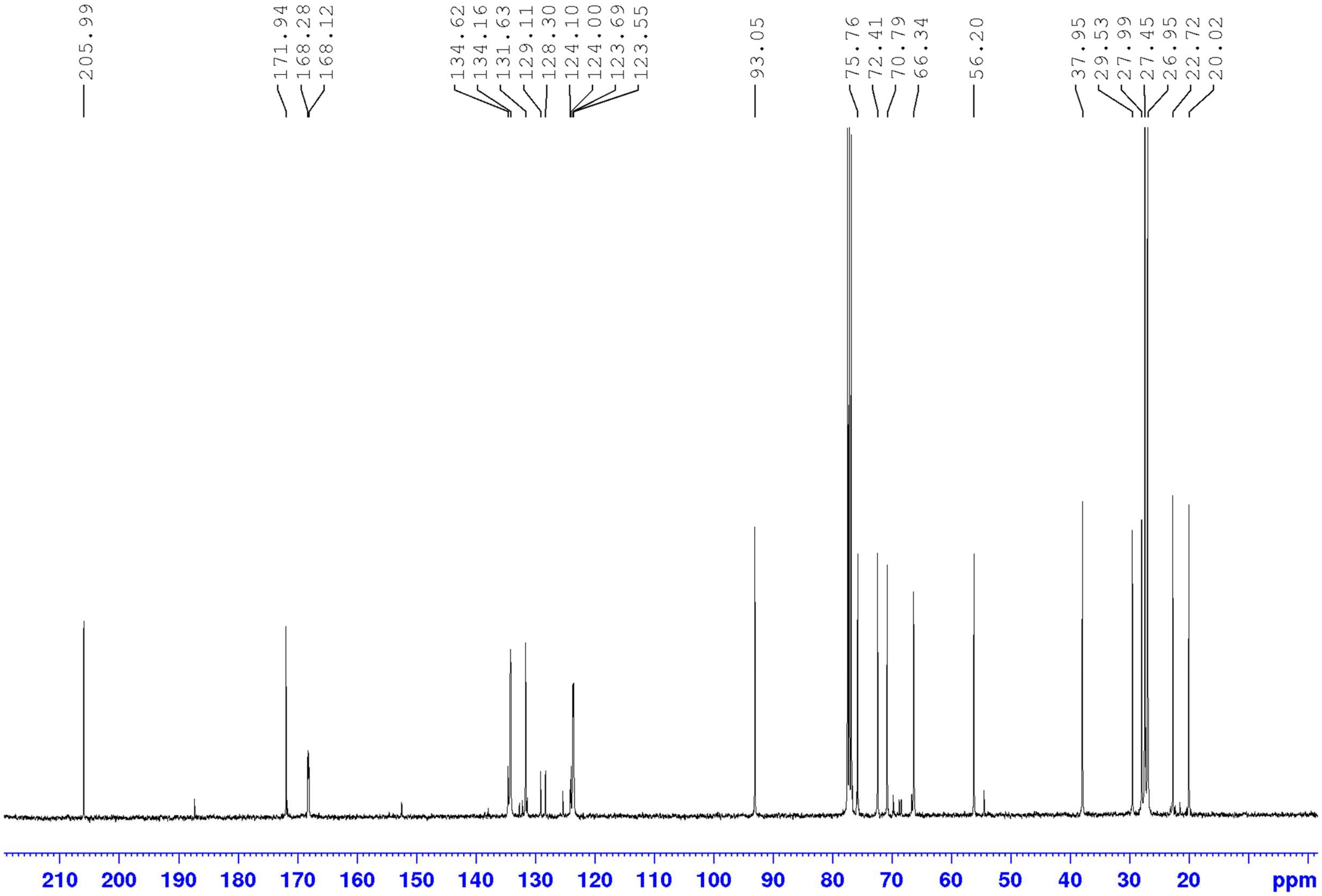

205.99
171.94
168.28
168.12
134.62
134.16
131.63
129.11
128.30
124.10
124.00
123.69
123.55
93.05
75.76
72.41
70.79
66.34
56.20
37.95
29.53
27.99
27.45
26.95
22.72
20.02
210 200 190 180 170 160 150 140 130 120 110 100 90 80 70 60 50 40 30 20 ppm

Compound **2**, ^{1}H-NMR

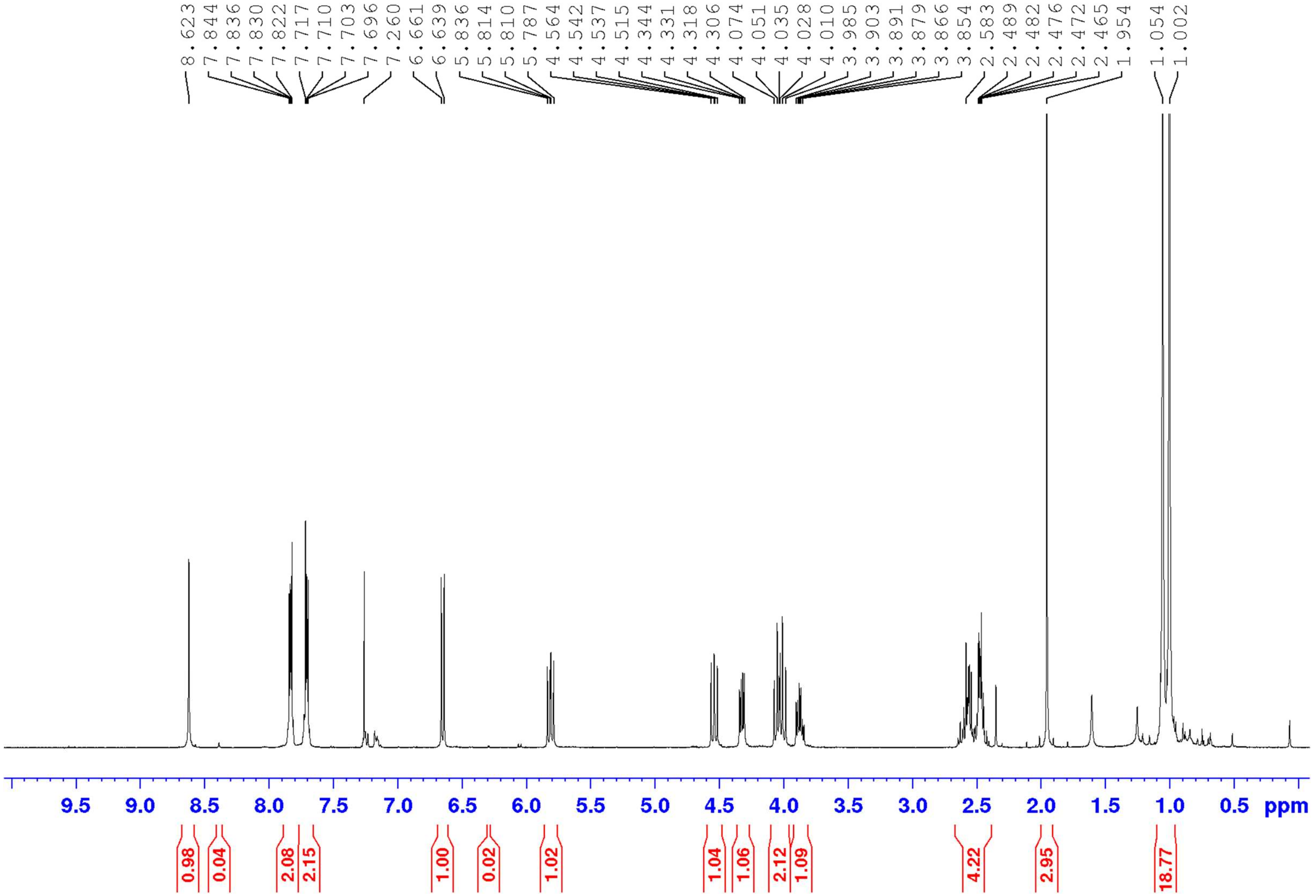

^{13}C-NMR

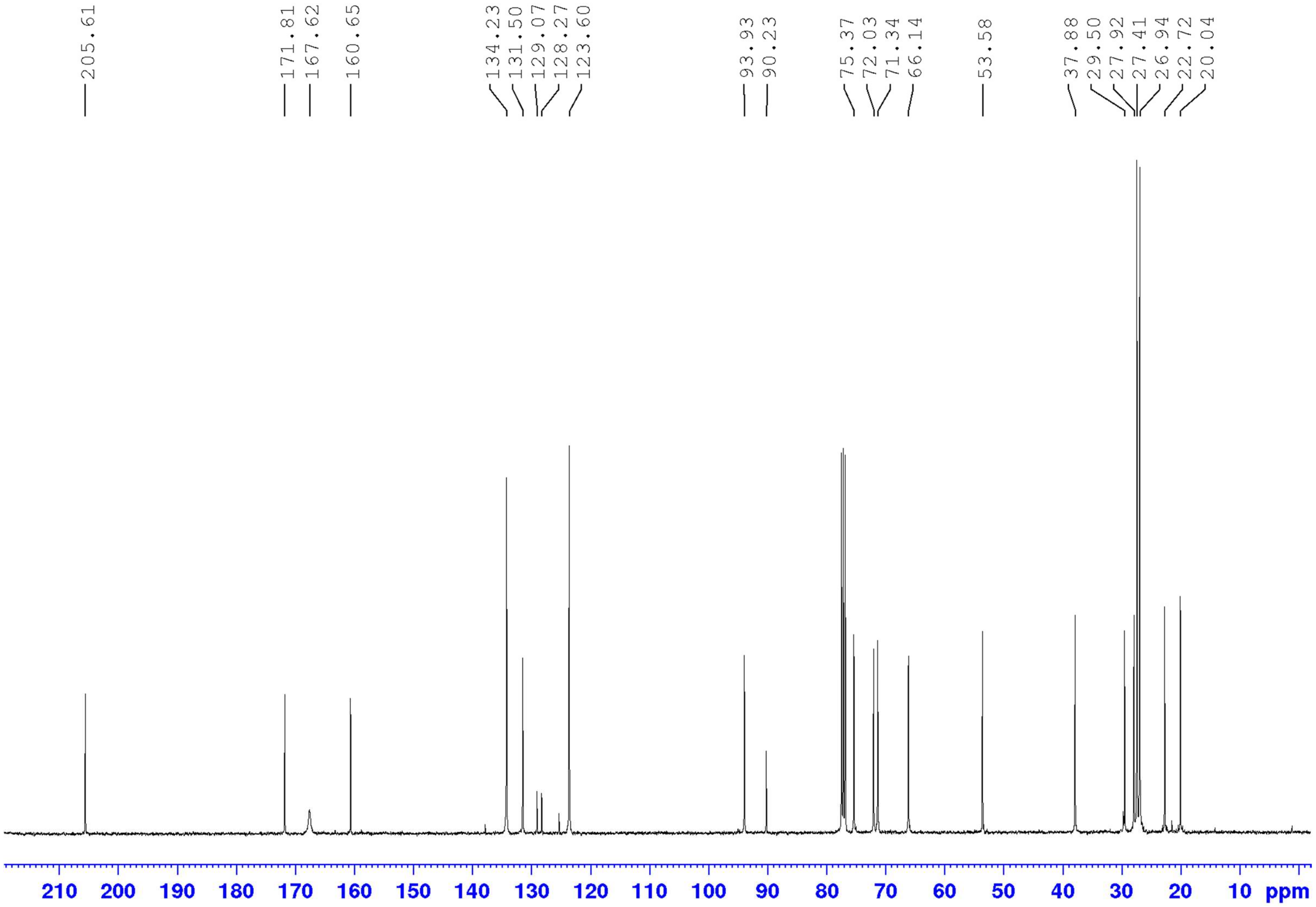
205.61
171.81
167.62
160.65
134.23
131.50
129.07
128.27
123.60
93.93
90.23
75.37
72.03
71.34
66.14
53.58
37.88
29.50
27.92
27.41
26.94
22.72
20.04
210 200 190 180 170 160 150 140 130 120 110 100 90 80 70 60 50 40 30 20 10 ppm

Compound **8**, ^{1}H-NMR

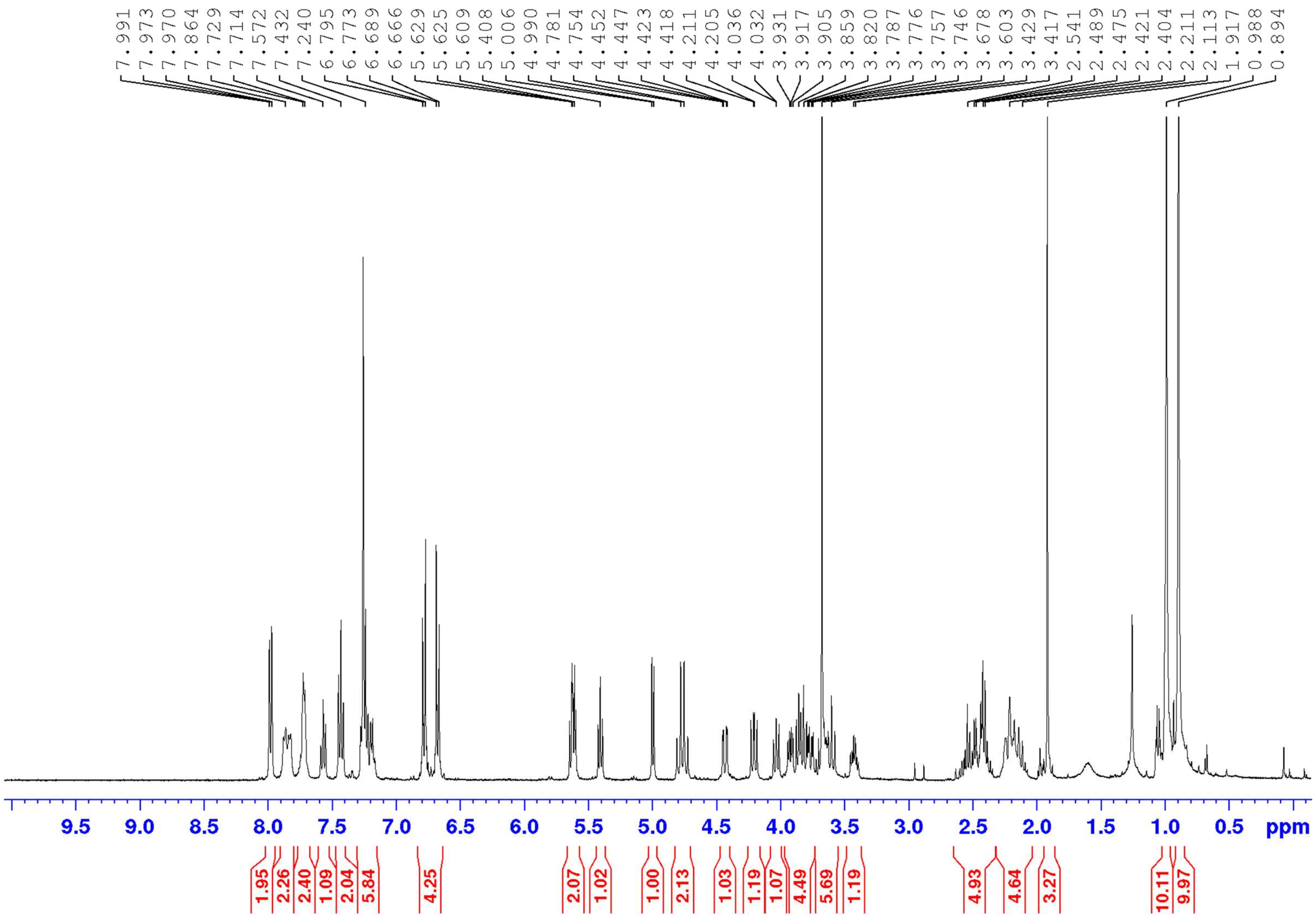

HSQC

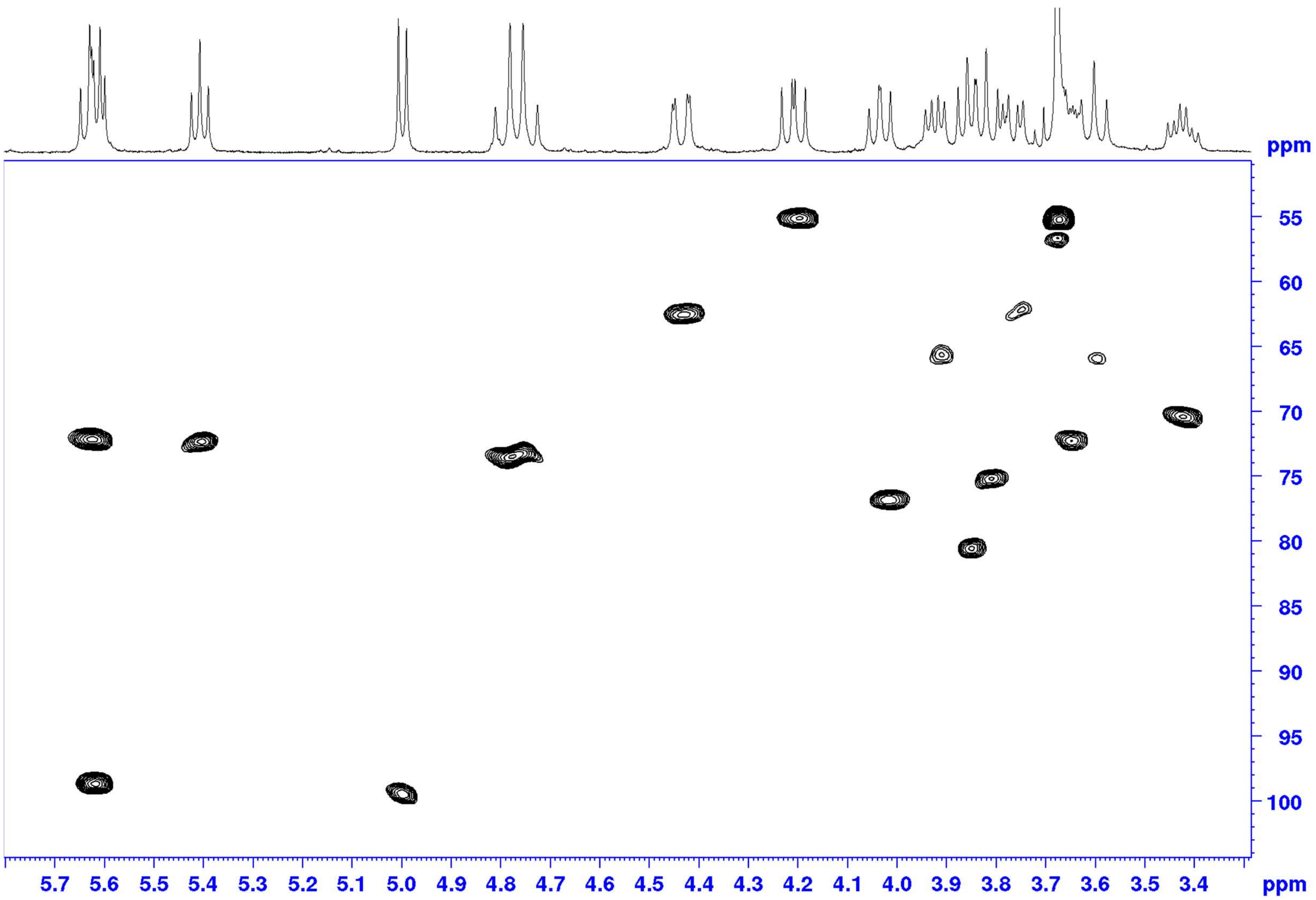

ppm
55
60
65
70
75
80
85
90
95
100
5.7 5.6 5.5 5.4 5.3 5.2 5.1 5.0 4.9 4.8 4.7 4.6 4.5 4.4 4.3 4.2 4.1 4.0 3.9 3.8 3.7 3.6 3.5 3.4
ppm

Compound **9**, ^{1}H-NMR

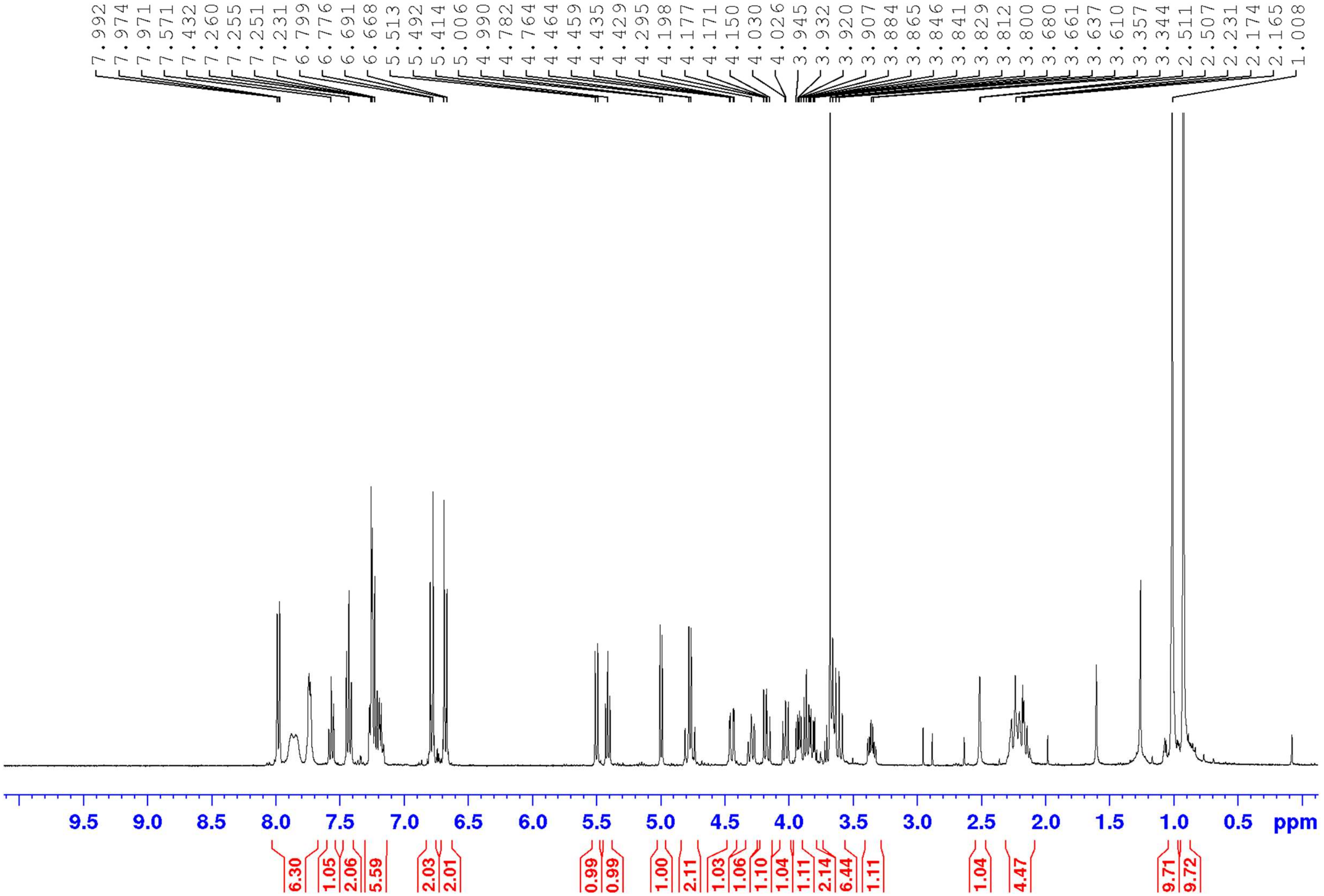

HSQC

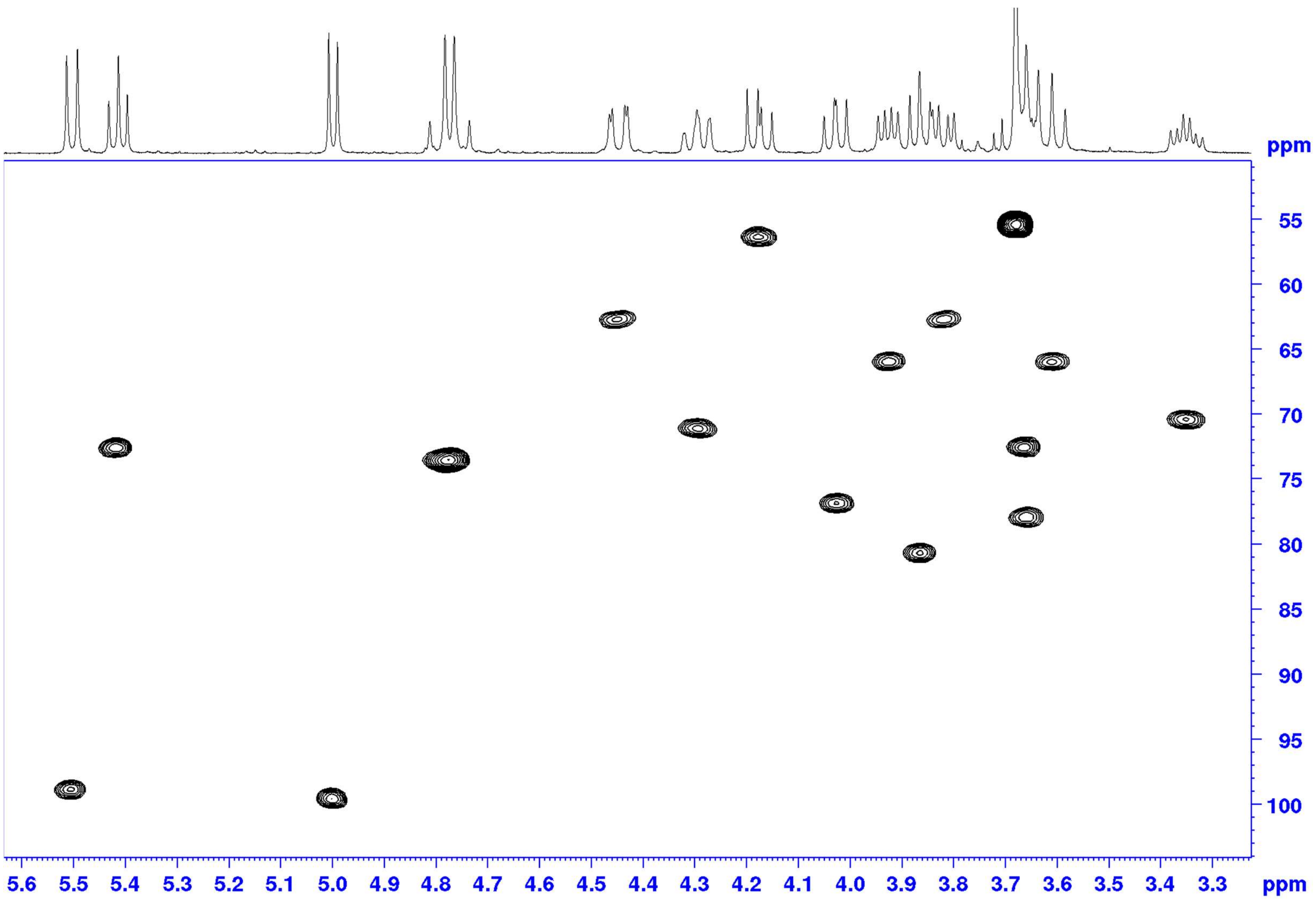

ppm
55
60
65
70
75
80
85
90
95
100
5.6 5.5 5.4 5.3 5.2 5.1 5.0 4.9 4.8 4.7 4.6 4.5 4.4 4.3 4.2 4.1 4.0 3.9 3.8 3.7 3.6 3.5 3.4 3.3
ppm

Compound **10**, ^{1}H-NMR

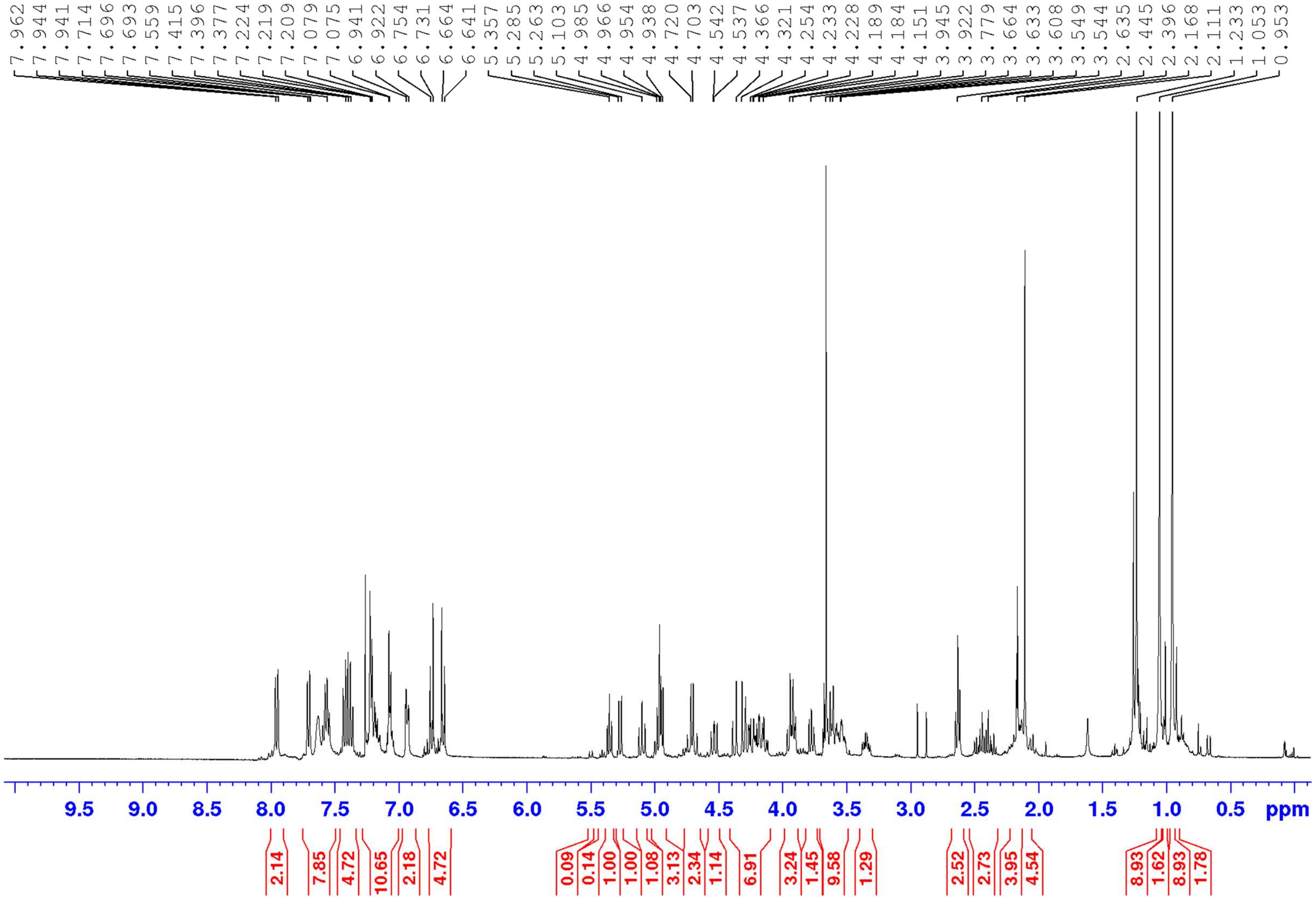

HSQC

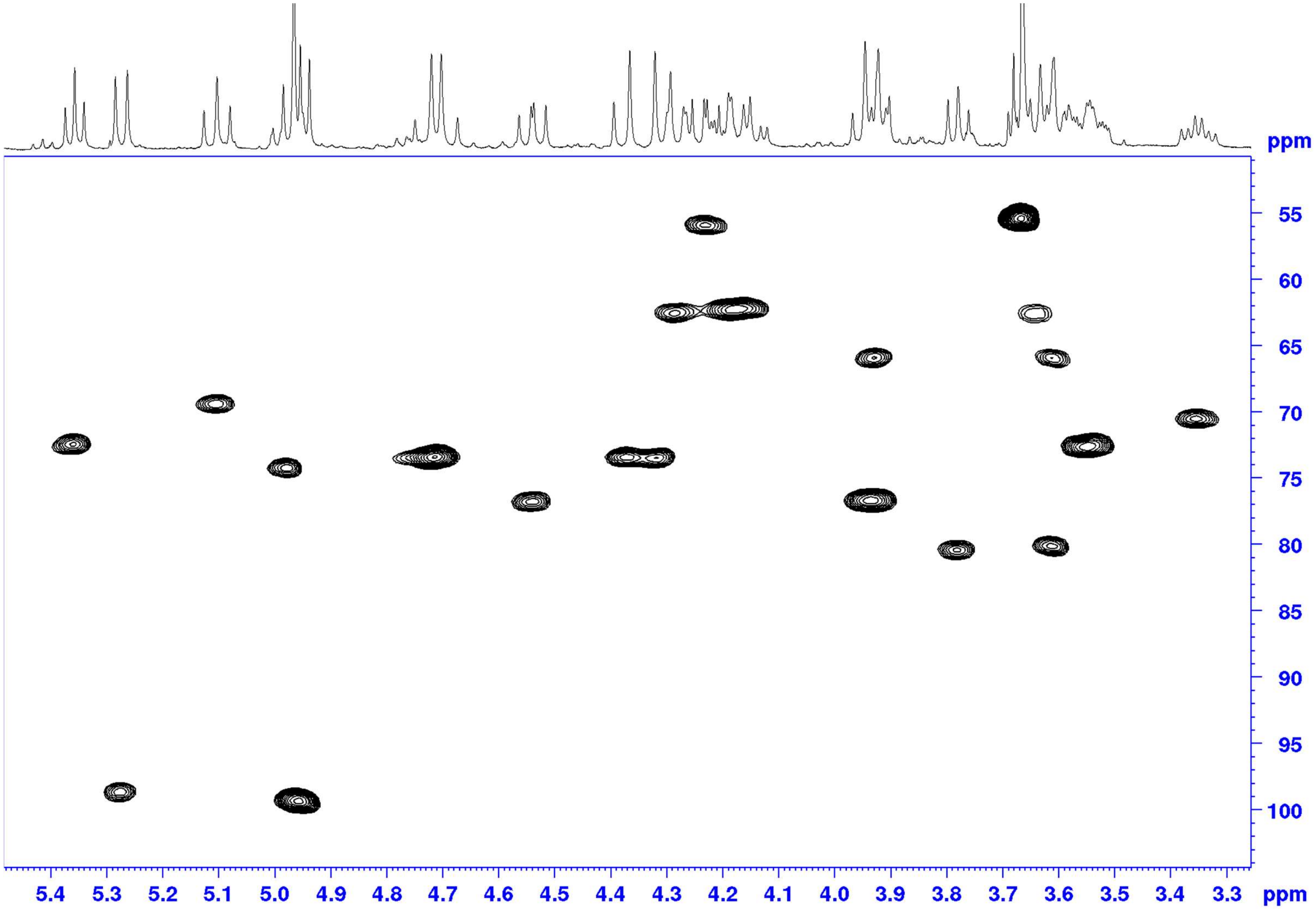

ppm
55
60
65
70
75
80
85
90
95
100
5.4 5.3 5.2 5.1 5.0 4.9 4.8 4.7 4.6 4.5 4.4 4.3 4.2 4.1 4.0 3.9 3.8 3.7 3.6 3.5 3.4 3.3
ppm

Compound **11**, ^{1}H-NMR

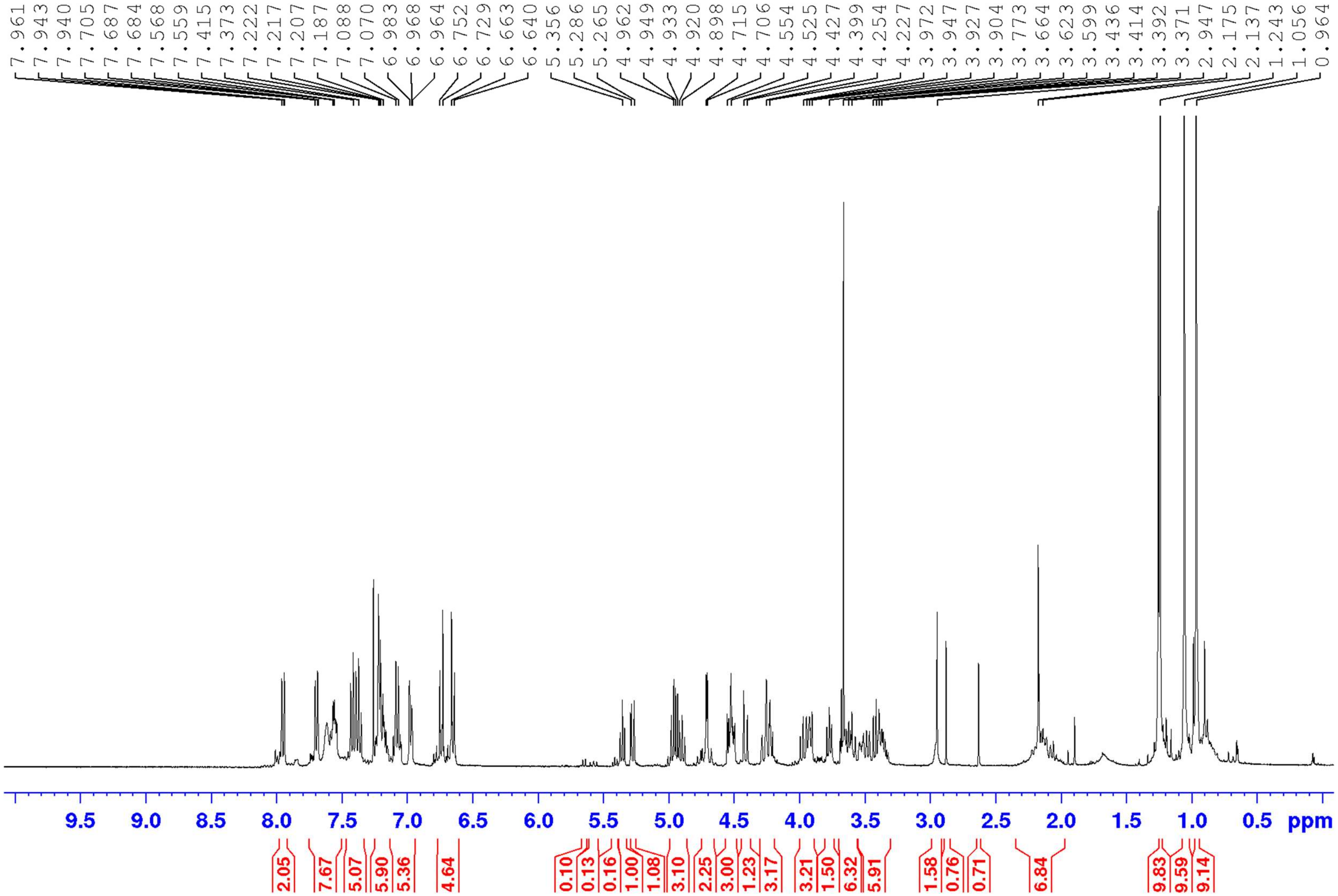

HSQC

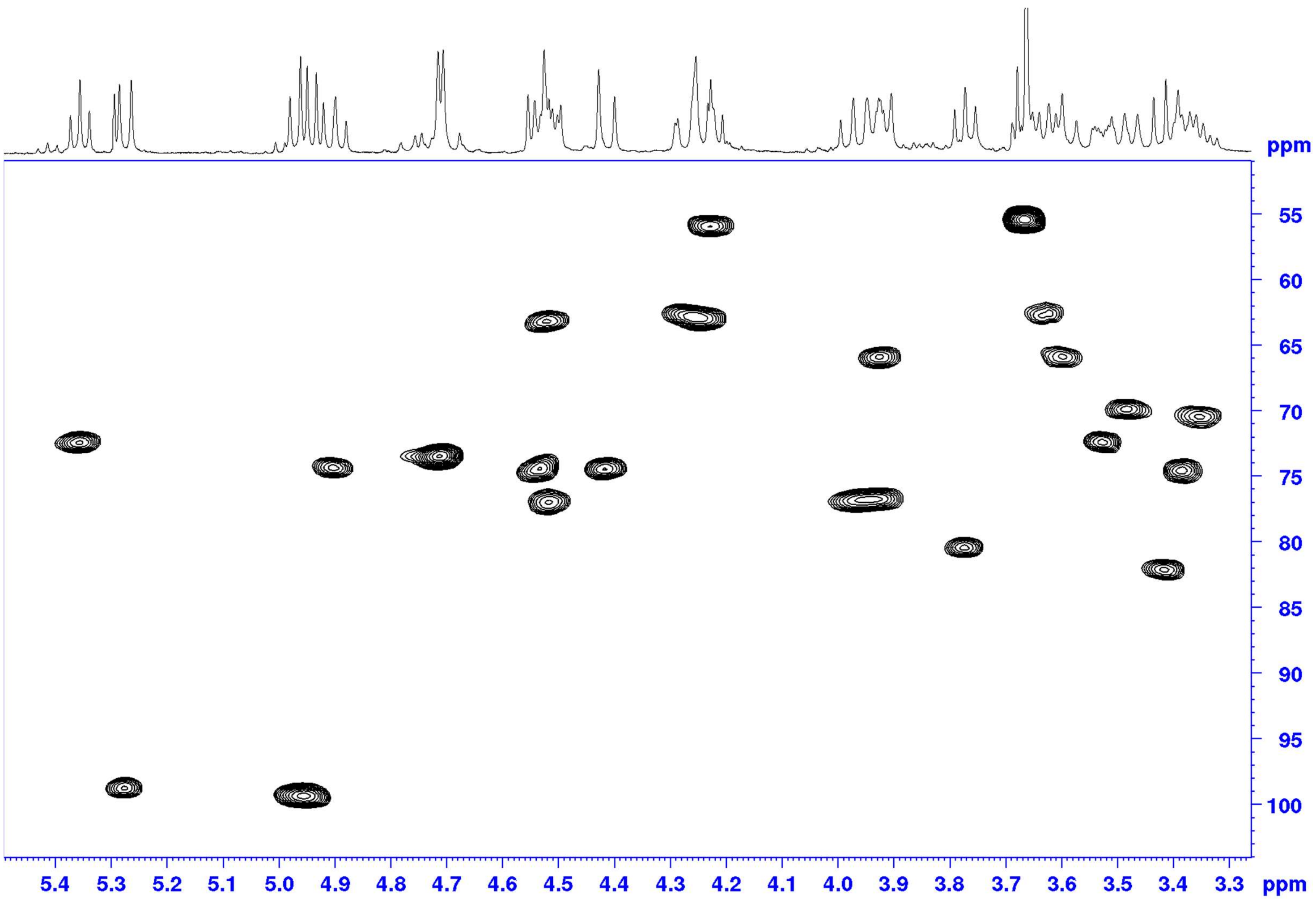
ppm
55
60
65
70
75
80
85
90
95
100
5.4 5.3 5.2 5.1 5.0 4.9 4.8 4.7 4.6 4.5 4.4 4.3 4.2 4.1 4.0 3.9 3.8 3.7 3.6 3.5 3.4 3.3 ppm

Compound **12,** ^{1}H-NMR

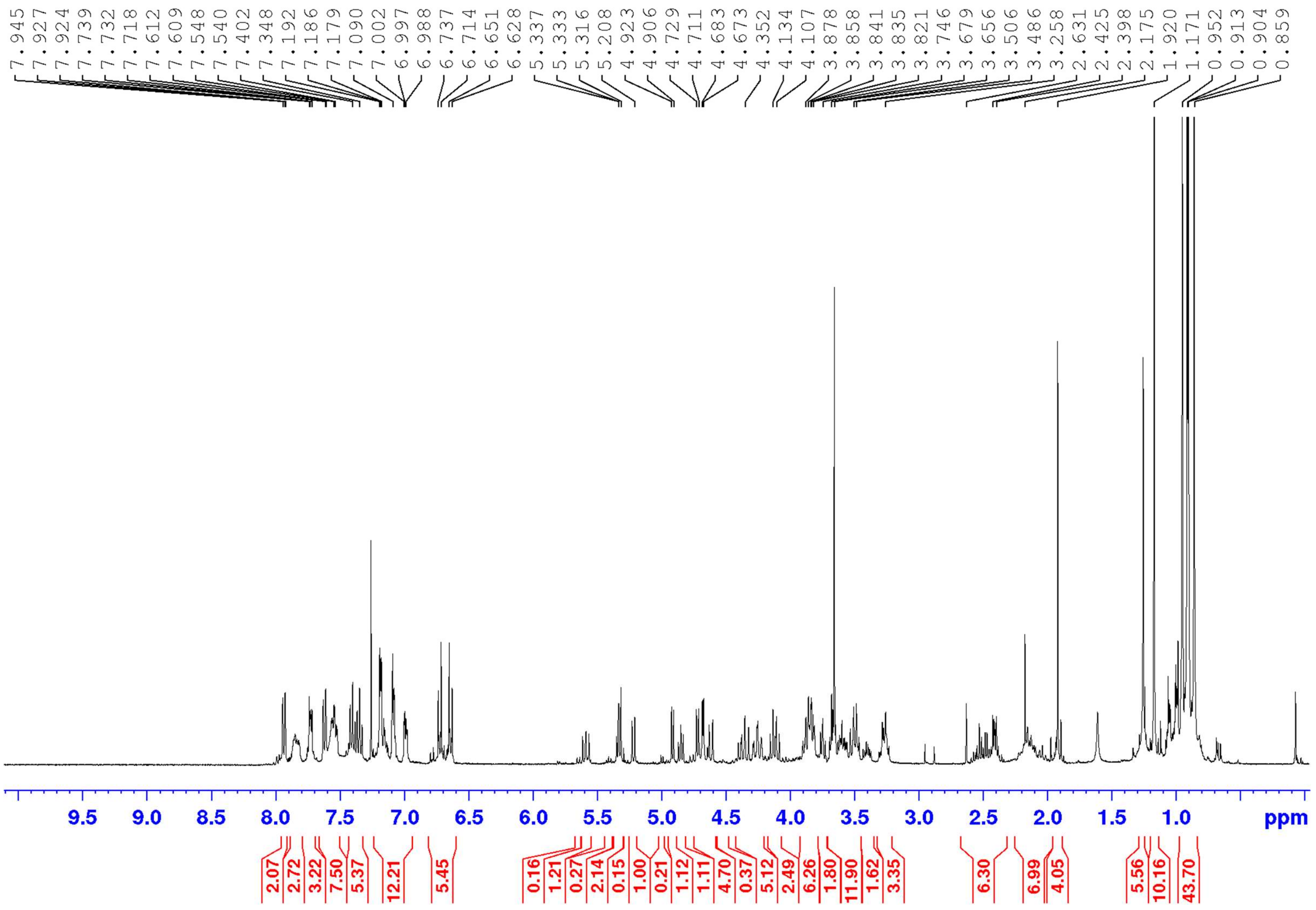

HSQC

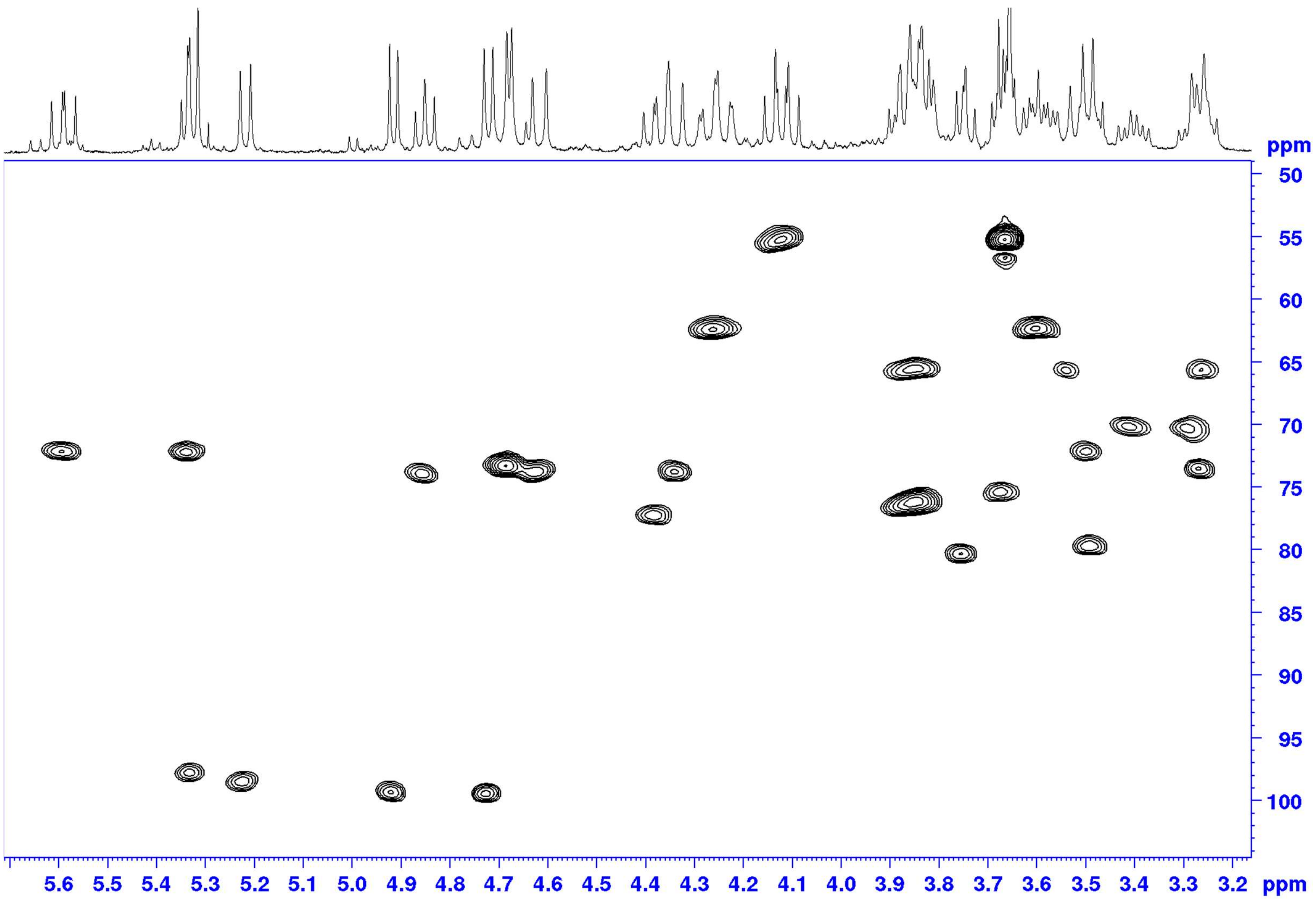

ppm
50
55
60
65
70
75
80
85
90
95
100
5.6 5.5 5.4 5.3 5.2 5.1 5.0 4.9 4.8 4.7 4.6 4.5 4.4 4.3 4.2 4.1 4.0 3.9 3.8 3.7 3.6 3.5 3.4 3.3 3.2 ppm

Compound **13,** ^{1}H-NMR

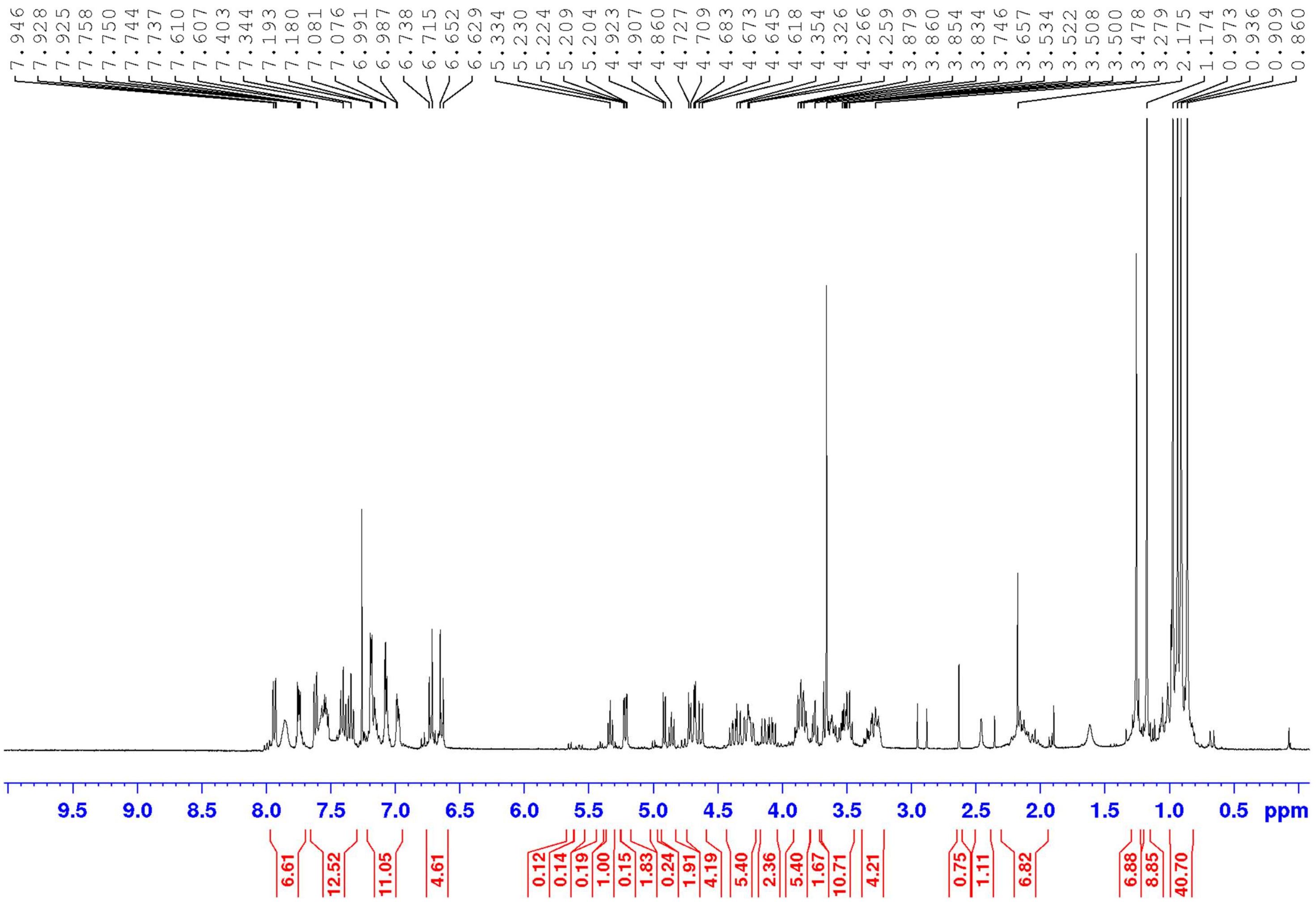

HSQC

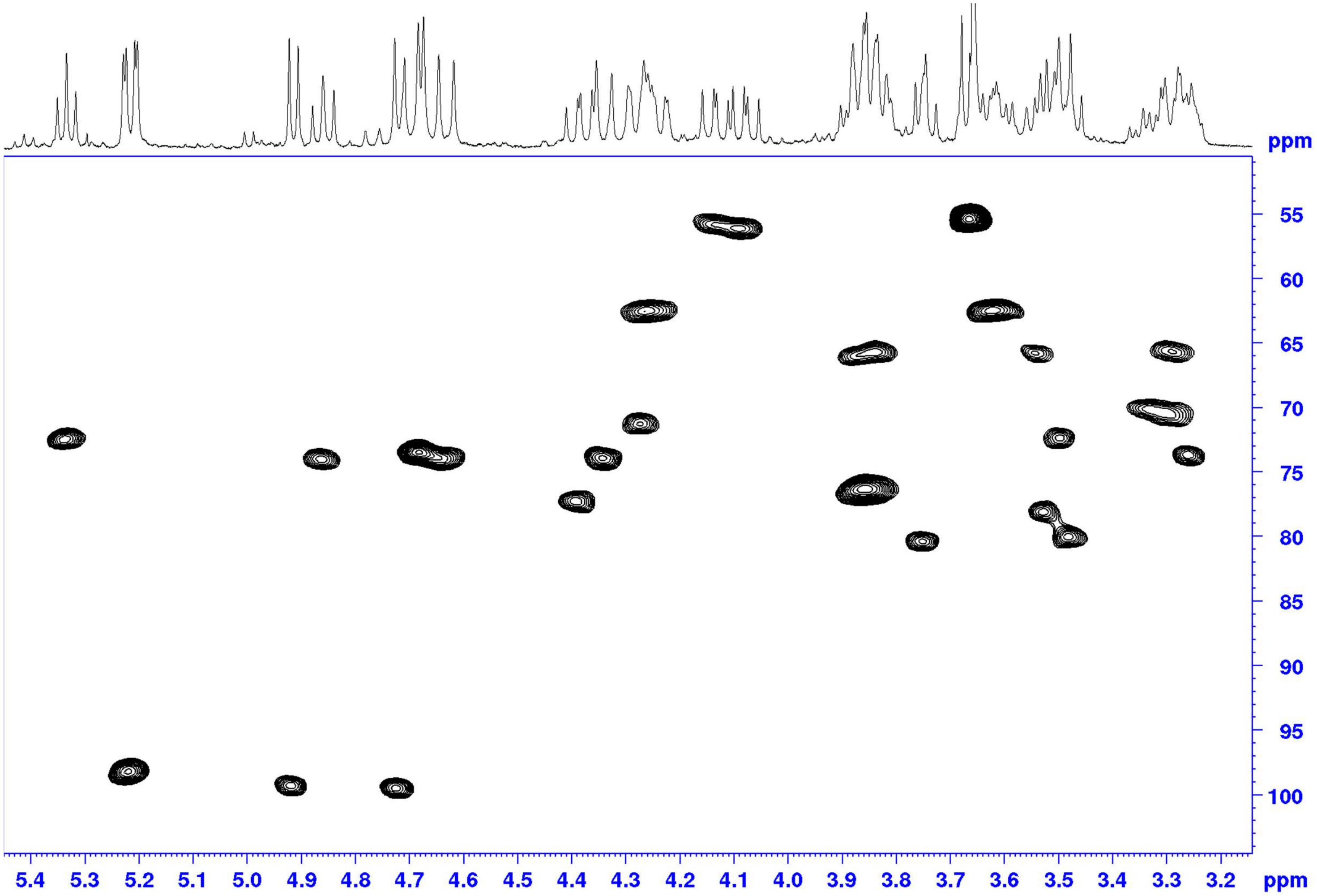

ppm
55
60
65
70
75
80
85
90
95
100
5.4 5.3 5.2 5.1 5.0 4.9 4.8 4.7 4.6 4.5 4.4 4.3 4.2 4.1 4.0 3.9 3.8 3.7 3.6 3.5 3.4 3.3 3.2
ppm

Compound **14**, ^{1}H-NMR

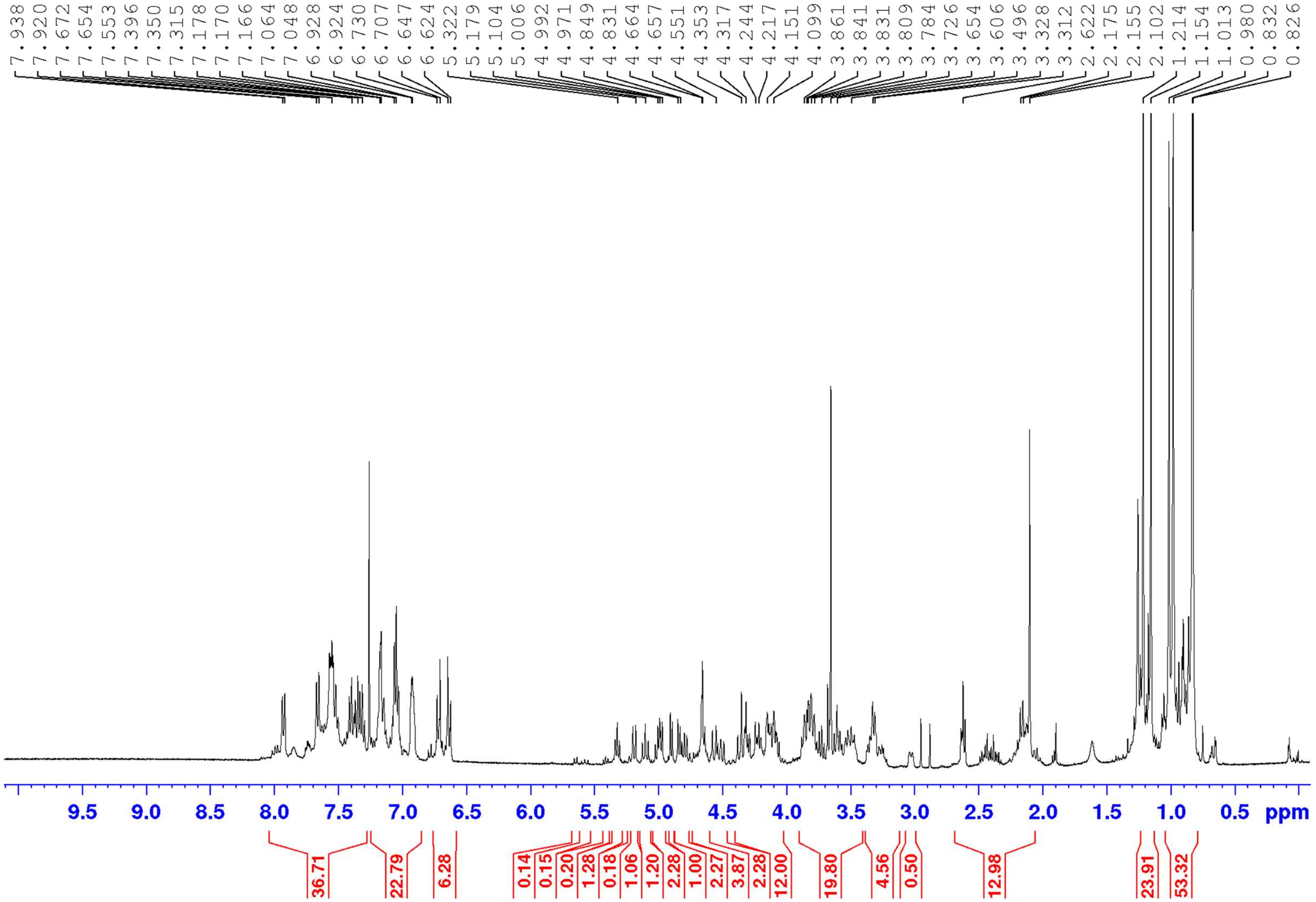

HSQC

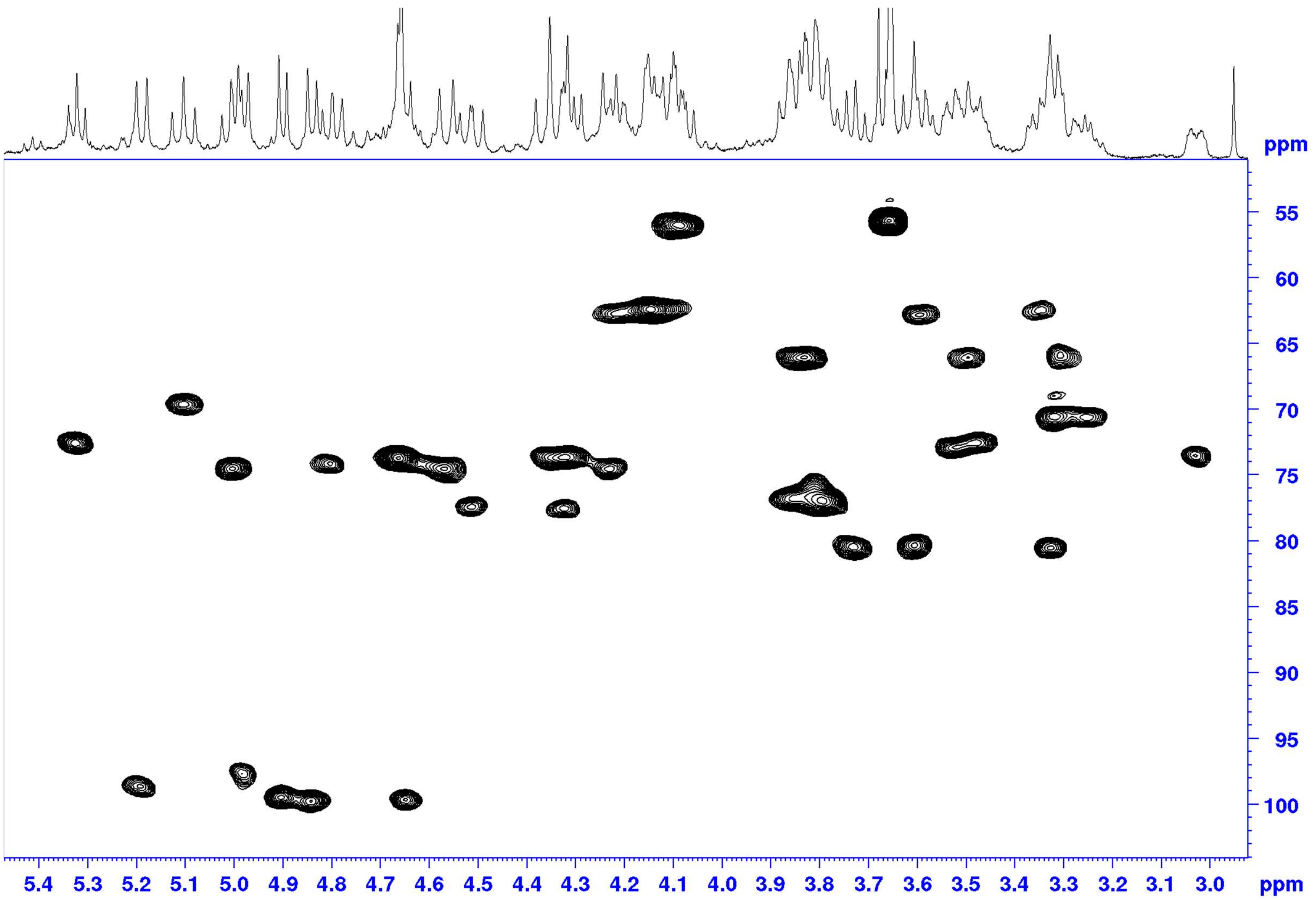

ppm
55
60
65
70
75
80
85
90
95
100
5.4 5.3 5.2 5.1 5.0 4.9 4.8 4.7 4.6 4.5 4.4 4.3 4.2 4.1 4.0 3.9 3.8 3.7 3.6 3.5 3.4 3.3 3.2 3.1 3.0
ppm

Compound **15**, ^{1}H-NMR

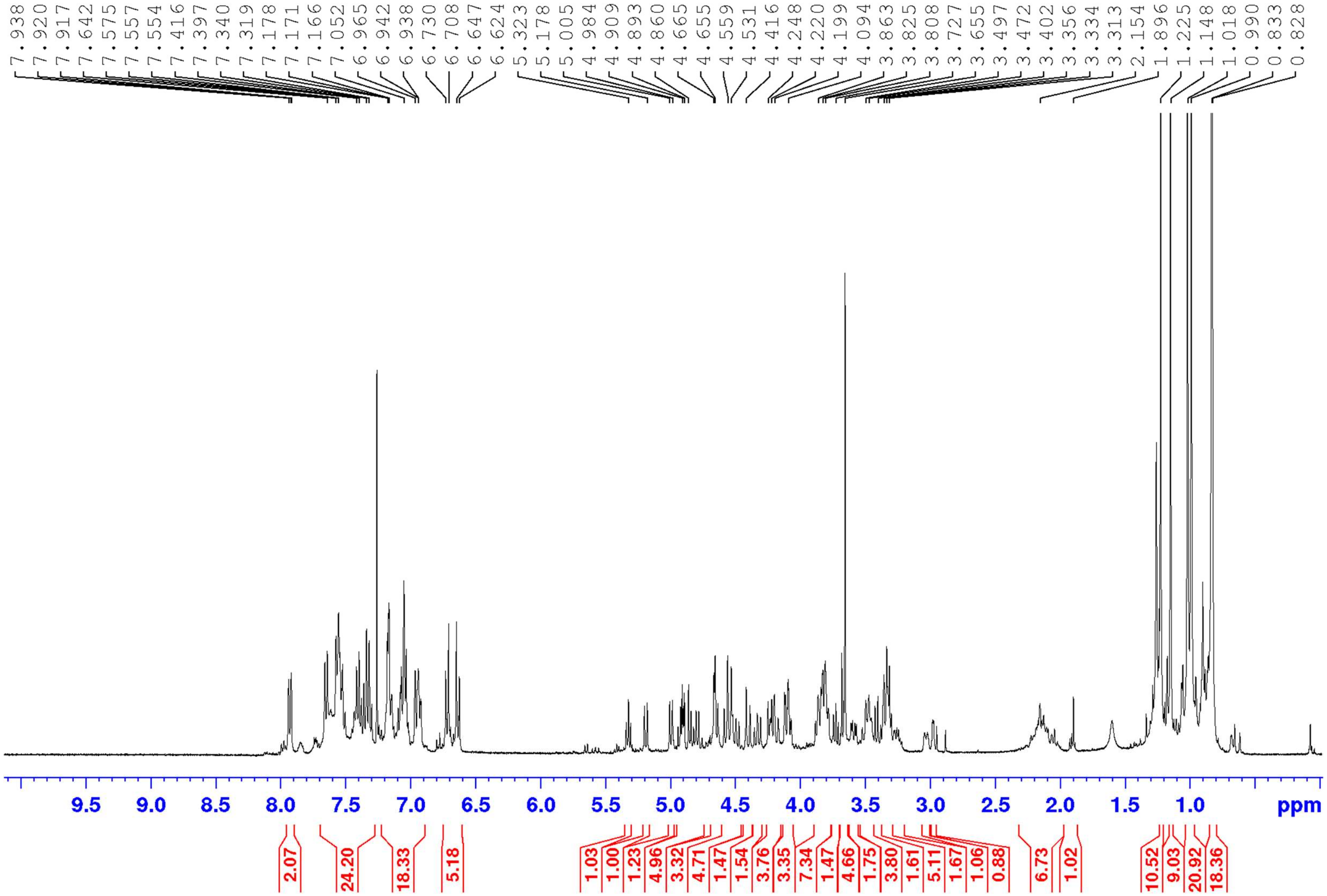

HSQC

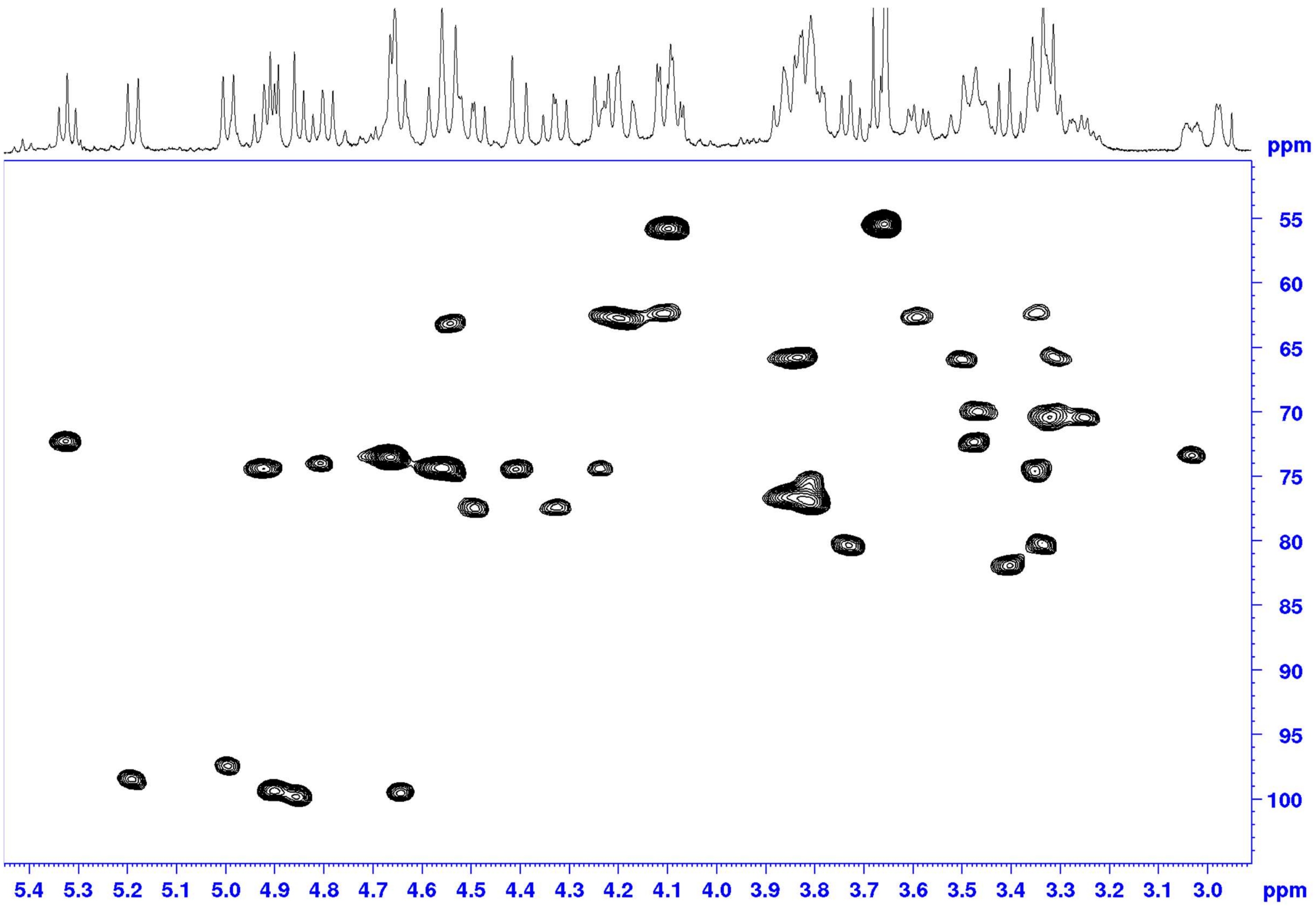

ppm
55
60
65
70
75
80
85
90
95
100
5.4
5.3
5.2
5.1
5.0
4.9
4.8
4.7
4.6
4.5
4.4
4.3
4.2
4.1
4.0
3.9
3.8
3.7
3.6
3.5
3.4
3.3
3.2
3.1
3.0
ppm

Compound **16,** ^{1}H-NMR

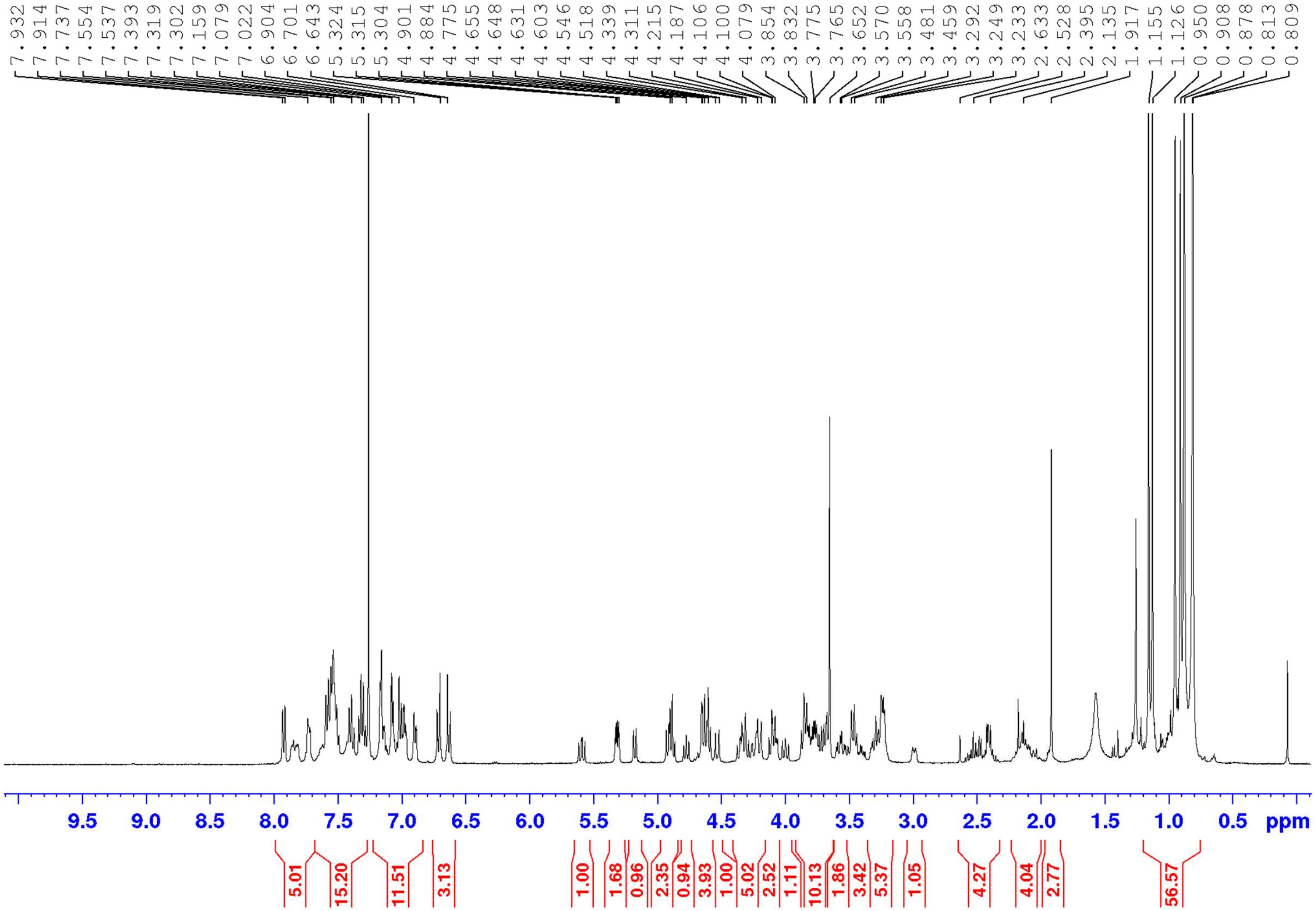

^{1}H-NMR

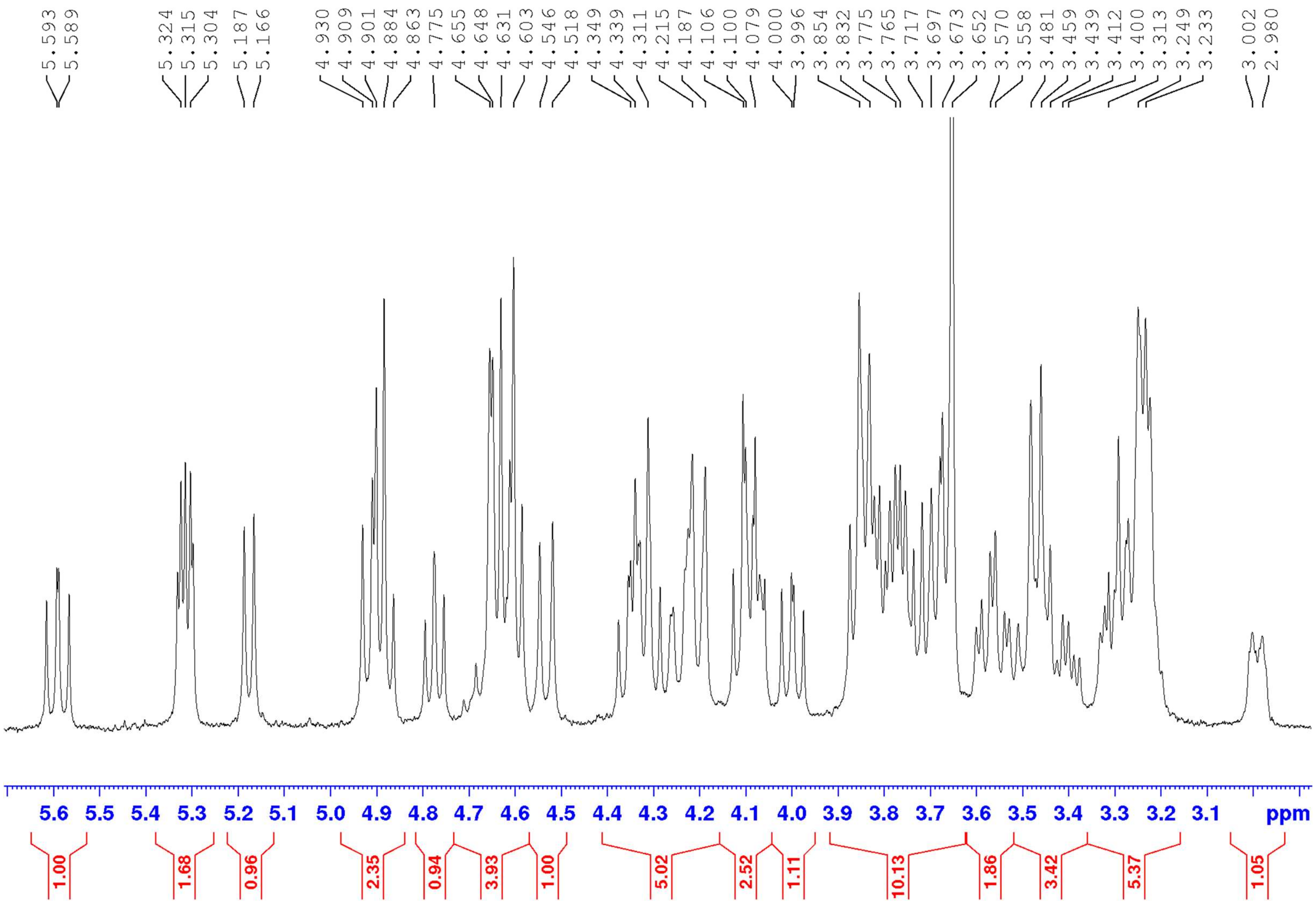

HSQC

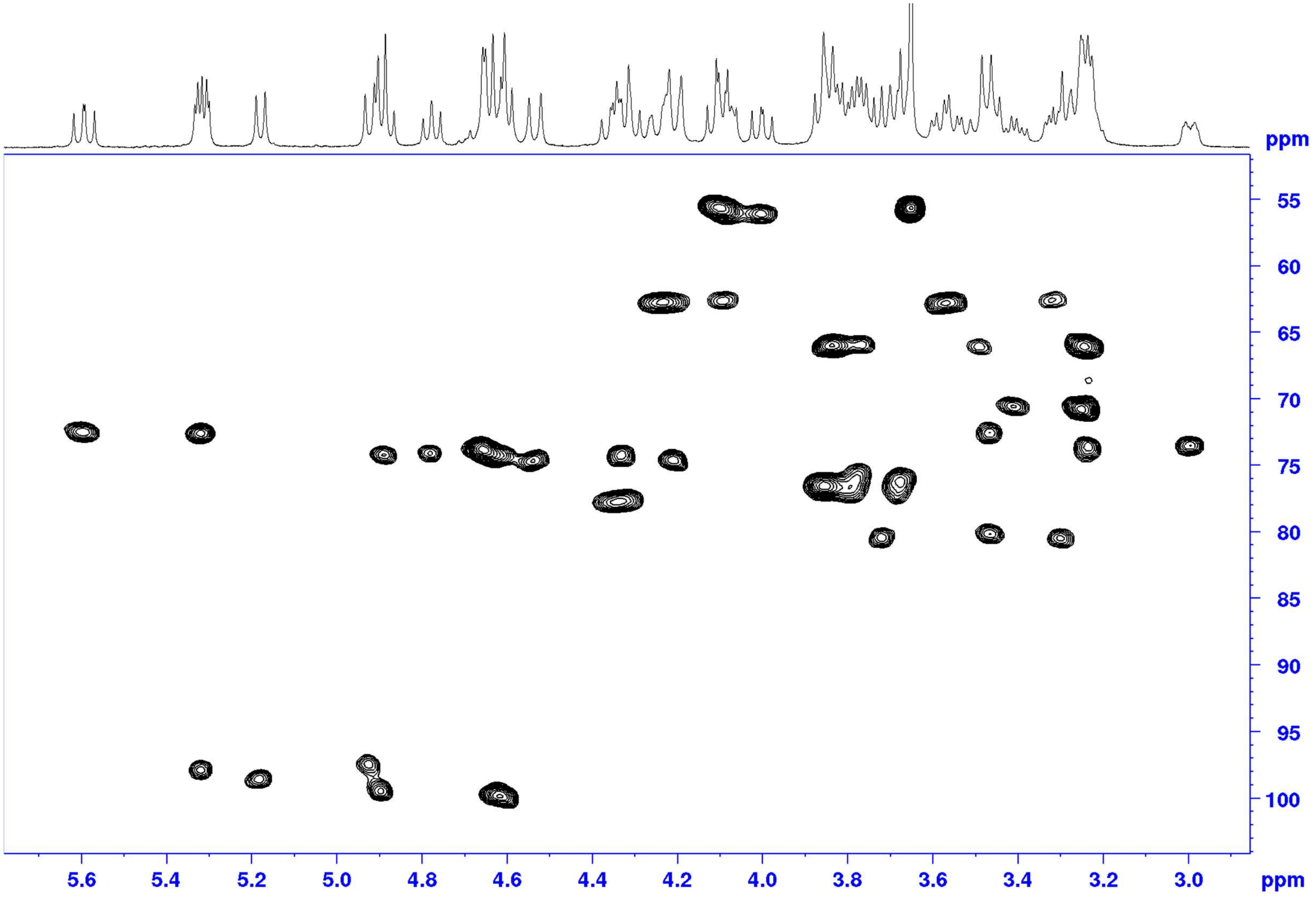
ppm
55
60
65
70
75
80
85
90
95
100
5.6
5.4
5.2
5.0
4.8
4.6
4.4
4.2
4.0
3.8
3.6
3.4
3.2
3.0
ppm

^{13}C-NMR

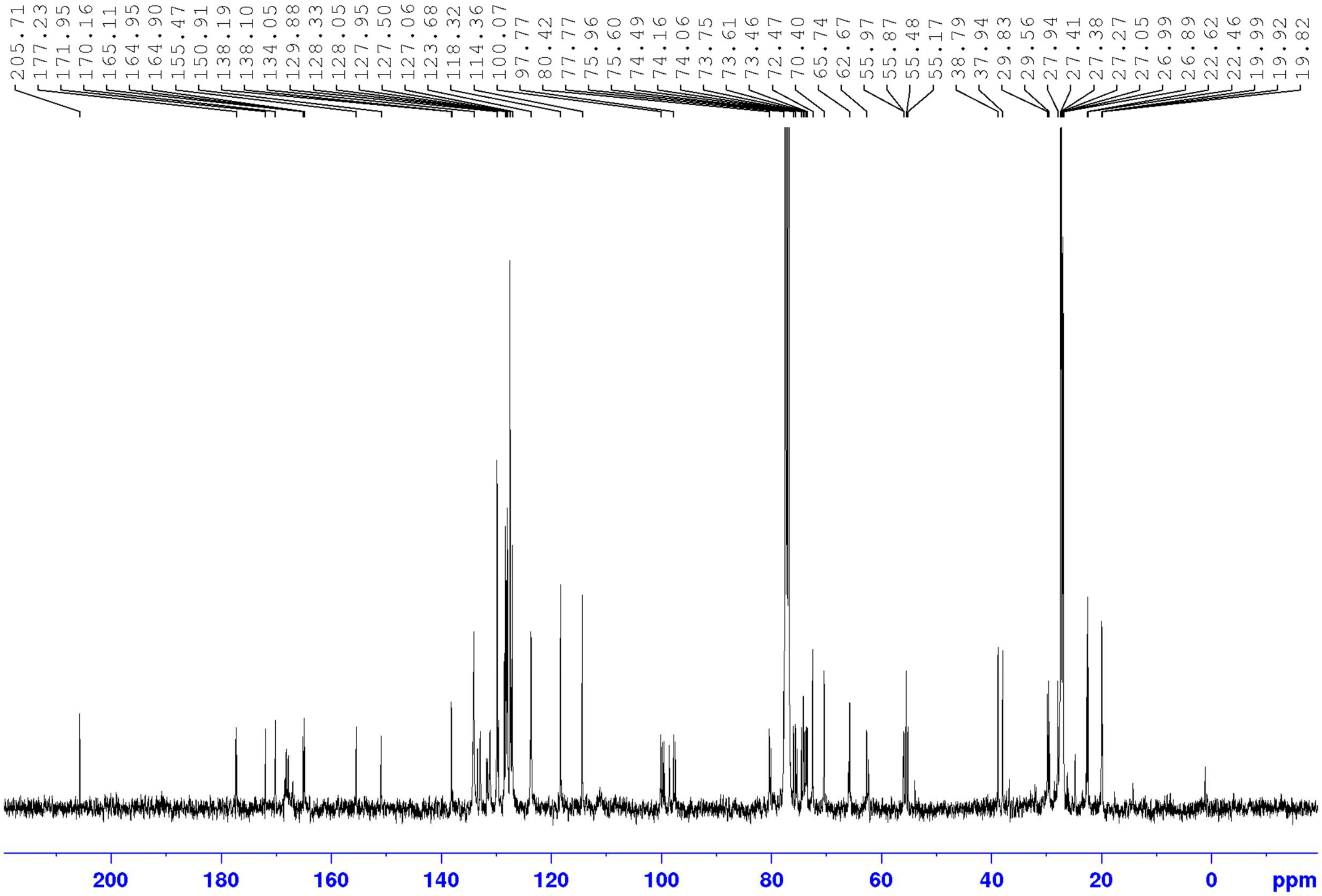
205.71
177.23
171.95
170.16
165.11
164.95
164.90
155.47
150.91
138.19
138.10
134.05
129.88
128.33
128.05
127.95
127.50
127.06
123.68
118.32
114.36
100.07
97.77
80.42
77.77
75.96
75.60
74.49
74.16
74.06
73.75
73.61
73.46
72.47
70.40
65.74
62.67
55.97
55.87
55.48
55.17
38.79
37.94
29.83
29.56
27.94
27.41
27.38
27.27
27.05
26.99
26.89
22.62
22.46
19.99
19.92
19.82
200
180
160
140
120
100
80
60
40
20
0
ppm

Compound **17**, ^{1}H-NMR

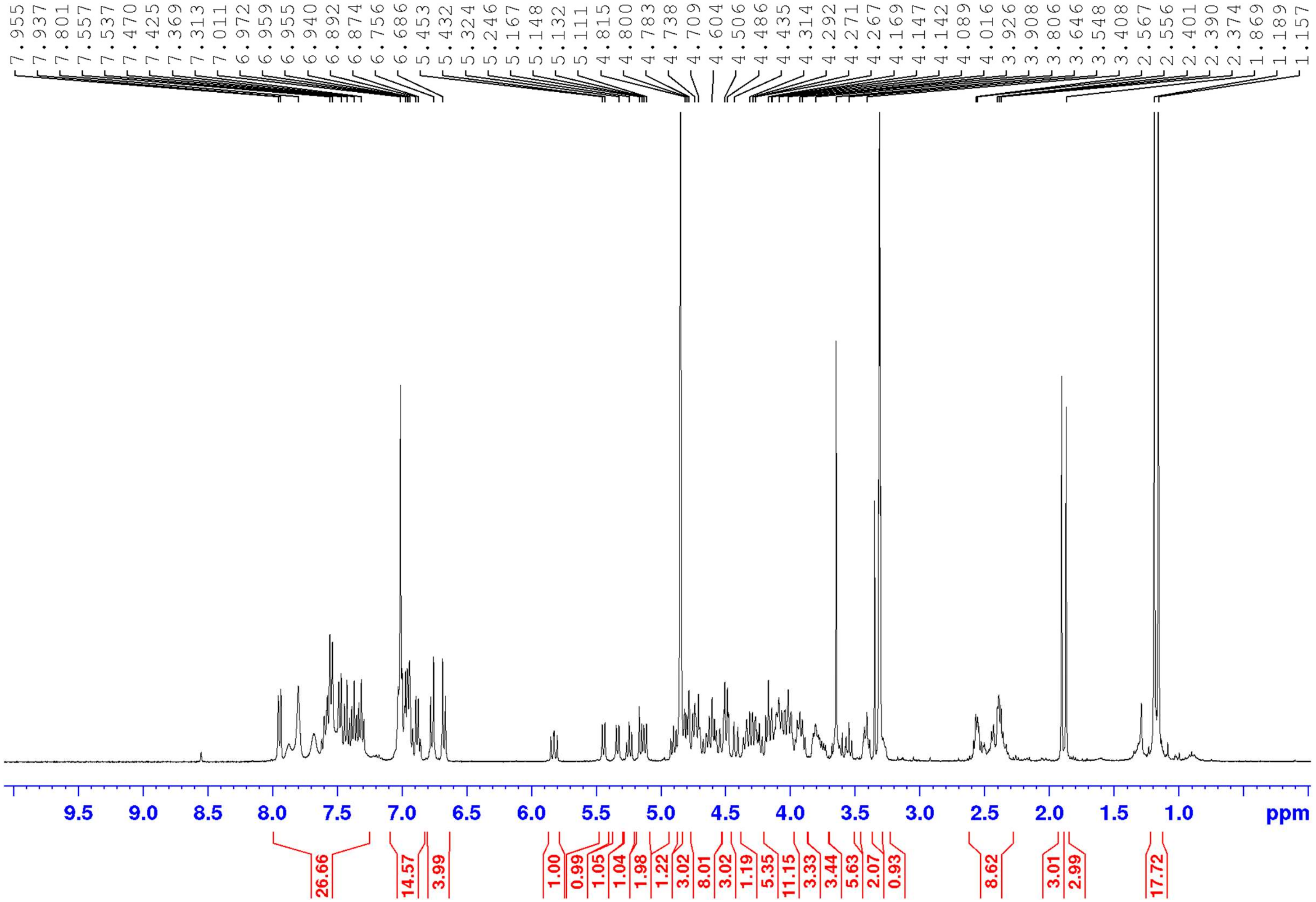

^{1}H-NMR

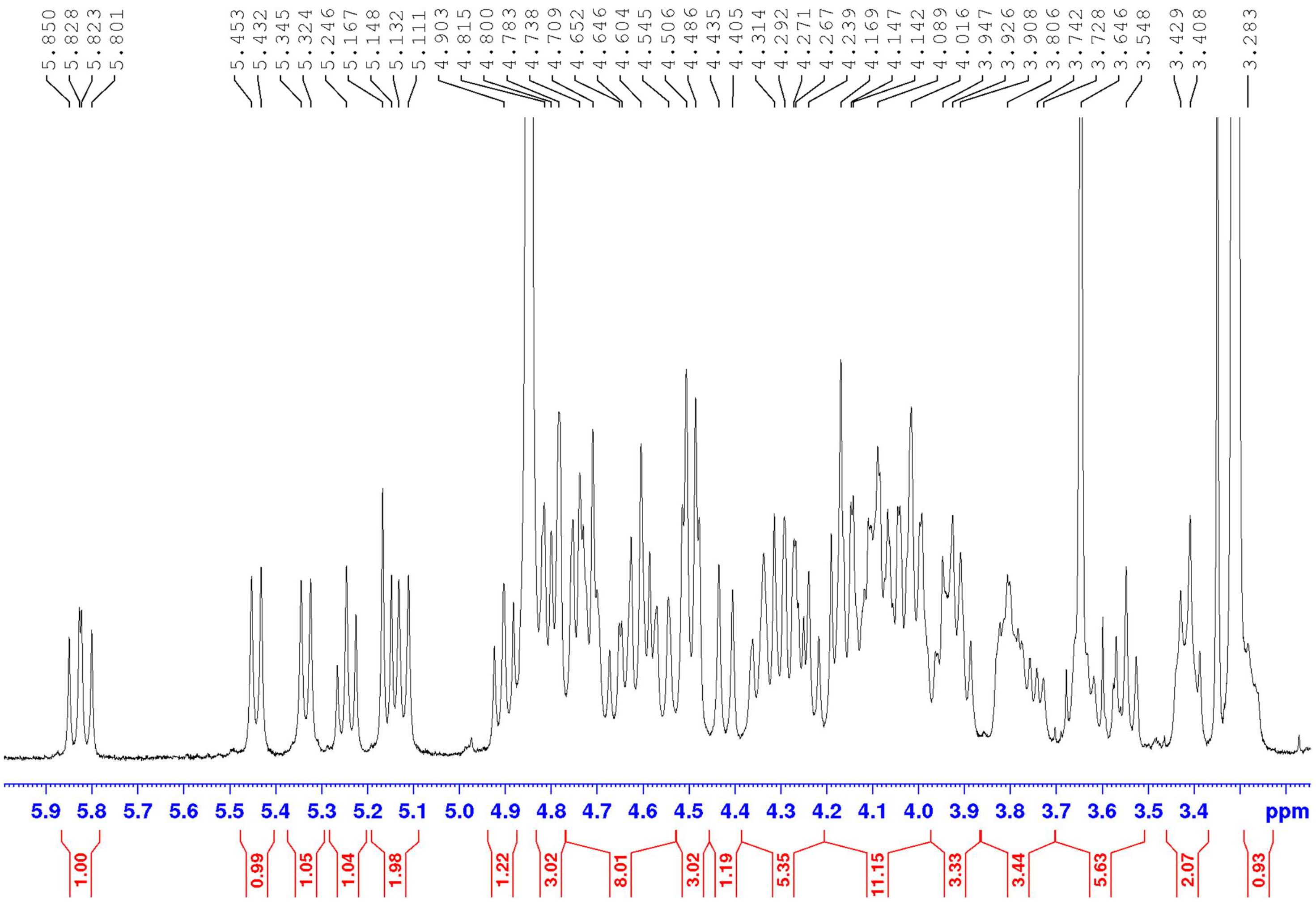

HSQC

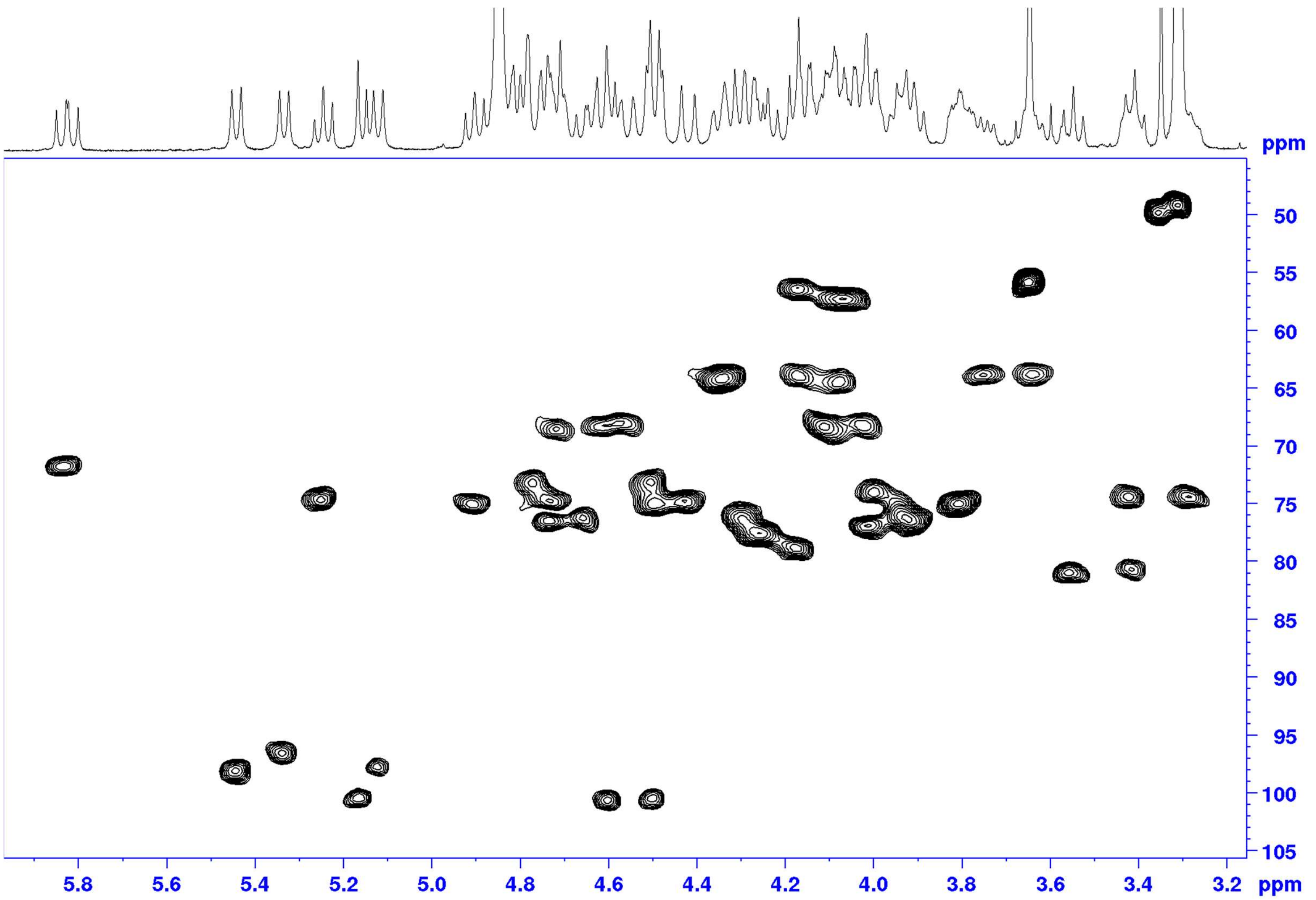
ppm
50
55
60
65
70
75
80
85
90
95
100
105
5.8
5.6
5.4
5.2
5.0
4.8
4.6
4.4
4.2
4.0
3.8
3.6
3.4
3.2
ppm

^{13}C-NMR

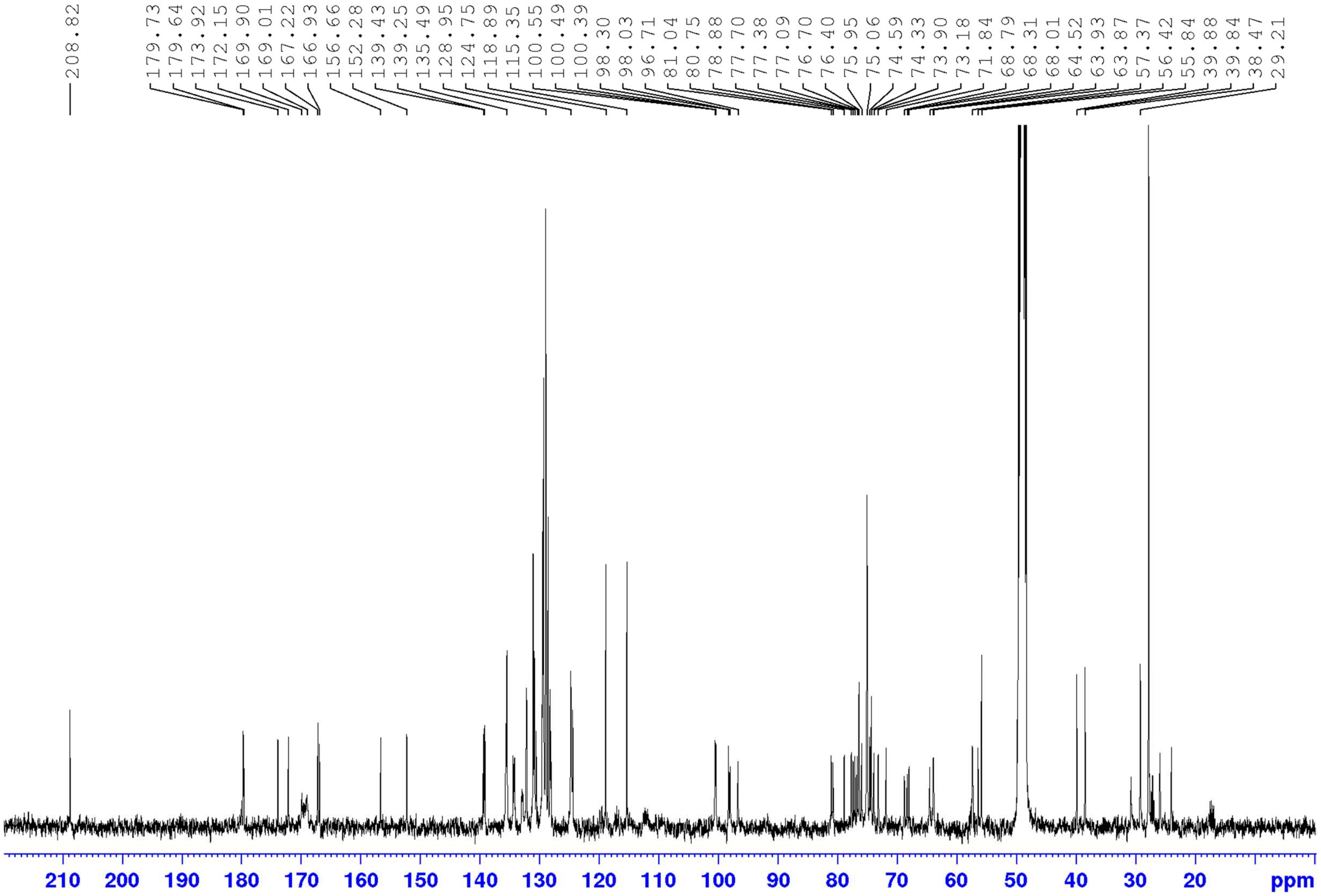
208.82
179.73
179.64
173.92
172.15
169.90
169.01
167.22
166.93
156.66
152.28
139.43
139.25
135.49
128.95
124.75
118.89
115.35
100.55
100.49
100.39
98.30
98.03
96.71
81.04
80.75
78.88
77.70
77.38
77.09
76.70
76.40
75.95
75.06
74.59
74.33
73.90
73.18
71.84
68.79
68.31
68.01
64.52
63.93
63.87
57.37
56.42
55.84
39.88
39.84
38.47
29.21
210
200
190
180
170
160
150
140
130
120
110
100
90
80
70
60
50
40
30
20
ppm
